# The common cuckoo is an effective indicator of high bird species richness in Asia and Europe

**DOI:** 10.1038/s41598-017-04794-3

**Published:** 2017-06-29

**Authors:** Federico Morelli, Anders Pape Møller, Emma Nelson, Yanina Benedetti, Wei Liang, Petra Šímová, Marco Moretti, Piotr Tryjanowski

**Affiliations:** 1 Czech University of Life Sciences Prague, Faculty of Environmental Sciences, Department of Applied Geoinformatics and Spatial Planning, Kamýcká 129, CZ-165 00 Prague 6, Czech Republic; 2 Laboratoire d’Ecologie, Systématique et Evolution, CNRS UMR 8079, Université Paris-Sud, Bâtiment 362, F-91405 Orsay, Cedex France; 3 School of Medicine, Faculty of Health and Life Sciences, University of Liverpool, Whelan Building, Brownlow Hill, Liverpool, L69 3GB UK; 4 Ministry of Education Key Laboratory for Tropical Plant and Animal Ecology, College of Life Sciences, Hainan Normal University, Haikou, 571158 P.R. China; 5 Swiss Federal Research Institute WSL, Biodiversity and Conservation Biology, Zürcherstrasse 111, CH-8903 Birmensdorf, Switzerland; 6 Institute of Zoology, Poznan University of Life Sciences, Wojska Polskiego 71C, PL-60-625 Poznań, Poland

## Abstract

Common cuckoo *Cuculus canorus* is a charismatic bird species with a dominant presence in human culture: from folklore legends to nowadays there is evidence of cuckoos being a prime candidate as a surrogate of bird diversity. Recent studies demonstrated that the cuckoo can predict hotspots of taxonomic diversity and functional diversity of bird communities in European countries. In this study, we demonstrated that the cuckoo is an excellent bioindicator at multi-spatial scale, extending cuckoo surrogacy from Europe to Asia. Even using three different survey methods (transect, square, point counts), comparing the new findings with results of our research in Europe, sites where the cuckoo is present were characterized by greater species richness, while the cuckoo was absent from sites with low species richness. The goodness of fit of models based on point counts ranged between 71 and 92%. Furthermore, the cuckoo population trend mirrors the average population trend and climate suitability of overall bird communities in Europe. The common cuckoo is therefore a suitable intercontinental bioindicator of hotspots of bird richness, even under climate change scenarios or in areas where the species co-occurs with other cuckoo species, opening a new avenue for standardized citizen science on bird biodiversity surveys worldwide.

## Introduction

Why is the common cuckoo *Cuculus canorus* a fascinating bird species for humans? What are the main reasons for the species being known as “a messenger of spring and morality”^1^, and why is it so conspicuous in human culture? A review of folklore shows clearly that the enigmatic cuckoo has driven the collective imagination of people throughout the world for thousands of years. First and foremost the cuckoo-call is associated with seasonal change. The timing of arrival of the cuckoo and the vigour of its calls were also used as indicators of the weather^2, 3^. In ancient Egypt, Aristophanes wrote that its arrival was associated with harvest time^2^. Cuckoo lore is intimately linked with change and metamorphosis^1–3^ and its call reflects the real world passing of time when seeds are transformed into crops, maidens are married and maids become mothers.

However, the most peculiar characteristic of cuckoos leaving its mark on human culture is related to the brood parasitic behavior of the species^4, 5^. Evidence from folklore suggests that the cuckoo is also associated with many species of birds (e.g., sparrows, magpies, ravens)^2, 6^, and these are often the species upon which the cuckoo relies to rear its young. Recent findings support the hypothesis that the species’ call mirrors the quality of the environment it inhabits^7, 8^. It appears that the cuckoo has become embedded in the collective imagination of people, as an indicator of the quality or richness of the environment. As such, science is becoming increasingly interested in the evolutionary and ecological aspects of its breeding strategy^9, 10^. Recent studies have shown that the occurrence of the cuckoo is associated with the presence of many bird species, suggesting that it is a powerful indicator of hotspots of bird species richness, perhaps even a better predictor than top predators^11^. In fact, new research has provided strong evidence for the cuckoo being a prime surrogate of bird diversity in many European countries and thus useful for ecological monitoring, increasing the importance of the species for development of conservation strategies^12, 13^.

Monitoring population trends is one of the most important tasks in ecology and for conservation planning^14–16^, however the use of ecological tools (i.e. surrogates) that facilitate this task is many times required in order to obtain data from the wild^17–20^. On the other hand, because the common cuckoo is a widespread species present also in Asia, it is interesting to study if the same pattern found in some European countries, linking the species with bird hotspots, also occurs in other continents. In Asia the species has potentially different host species than in many European countries. Furthermore, in Asia, where other cuckoo species are breeding, the common cuckoo is not the only parasite species, and for this reason it is subject to a greater competitive pressure than in Europe.

However, until now nothing was know about common cuckoo predictability in other continents, where the common cuckoo co-occurs in same habitat than other parasite cuckoo species. Then, the co-occurence of other cuckoo species can reduce the value of common cuckoo as proxy of species diversity in Asia. Furthermore, would be necessary to know if the trend of common cuckoo populations follows the overall trend of bird species in different countries, because this condition could make cuckoo a suitable indicator, even under climate change scenarios.

In this study, we tested the hypothesis that the common cuckoo may serve as an effective surrogate for bird taxonomic diversity in ten European and two Asian countries, comparing the predictive power of the species among countries. Furthermore, we tested whether the population trend of the cuckoo and the climate suitability trend also mirror the overall trend of bird communities in Europe. A deep understanding of connections linking this indicator species and biodiversity in general will provide new insights useful for addressing action plans for biodiversity conservation and management strategies of ecosystems^21–23^.

## Results

**Figure 1:**
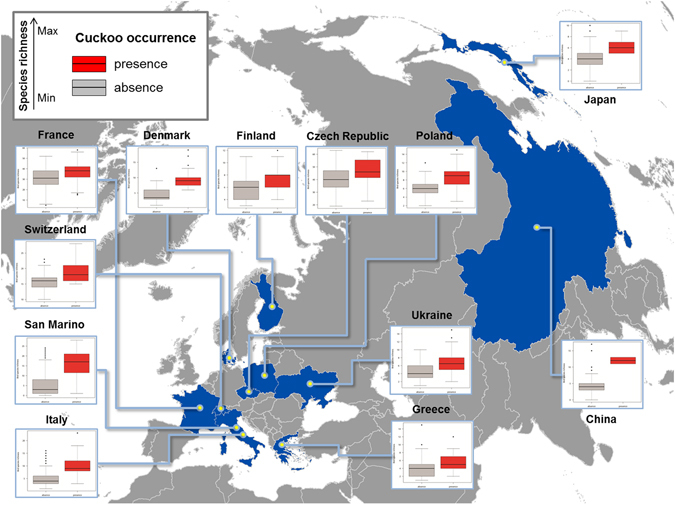
Comparison of average bird species richness between sample sites where cuckoos were present (red boxes) or absent (grey boxes) in twelve European and Asian countries. The box plots show medians, quartiles, 5- and 95-percentiles and extreme values. The map was generated with GIS soſtware (ArcGIS 10.1)^57^ with geographical background using data available under the Open Database Licence (“© OpenStreetMap and contributors”; cartography licensed as CC BY-SA) http://www.openstreetmap.org/copyright.

A total of 65,234 observations of bird occurrence in 3,592 sample sites in different environments were collected from ten European and two Asian countries. The maximum bird species richness per point count in all countries ranged from 12 species (Finland), to 28 species (San Marino and Switzerland). In European countries, the average bird species richness per site ranged from 4.0 ± 2 species (Greece) to 16.4 ± 3.3 species (Switzerland). The overall bird species richness was significantly positively correlated with number of non-host species in each sample site (r^2^ = 0.94, F = 7,132, df = 1, 470, p < 0.05). In Asian countries, the average bird species richness per site ranged from 4.1 ± 1.9 species (Japan) to 4.4 ± 2.1 species (China), with maximum values of 10 and 17 species for Japan and China, respectively (ESM, Table A, Fig. 1).

**Figure 2:**
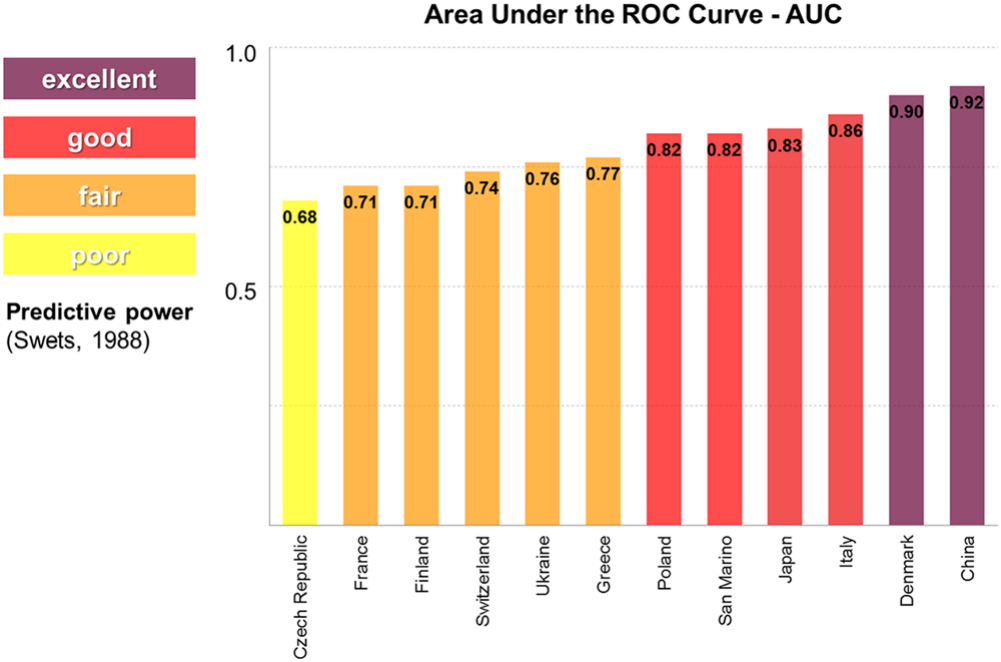
Predictive performance of models using bird species richness as a surrogate of cuckoo occurrence in twelve European and Asian countries. The height of the histogram columns represents the values of the area under the curve (AUC), that is the goodness of fit measure used in this study, indicating how well the model fits a set of observations.

Sample sites were treated as statistically independent observations because the spatial autocorrelation in all studied countries was not significant (Mantel test, 999 randomizations p > 0.05). In both European and Asian countries common cuckoo occurrence was strongly positively correlated with overall bird species richness (Table 1), with values of bird species richness always being higher in the sample sites where common cuckoos were presents (Fig. 1). The estimate for the variables “bird species richness” ranged across positive values of confidence intervals (Table 1). The performance of models using bird species richness to predict common cuckoo occurrence in European and Asian countries ranged between fair to excellent in all studies based on point counts, with slightly lower values in studies based on transects or point counts merged in squares (Fig. 2, ESM Figure A).

**Table 1:** Fixed-effect parameters in a Generalized Linear Mixed Model, accounting for cuckoo occurrence in relation to bird species richness (taxonomic diversity), in ten European and two Asian countries.

Variables	Estimate	CI	SE	z	p
(Intercept)	−3.197	−4.443/−1.952	0.635	−5.033	<0.05
Bird species richness	0.150	0.134/0.167	0.008	18.081	<0.05

The full model is based on 3592 sample sites. Random effects: Country (groups = 12) and dominant environment (groups = 5). CI: confidence interval (lower/upper); SE: standard error.

**Figure 3:**
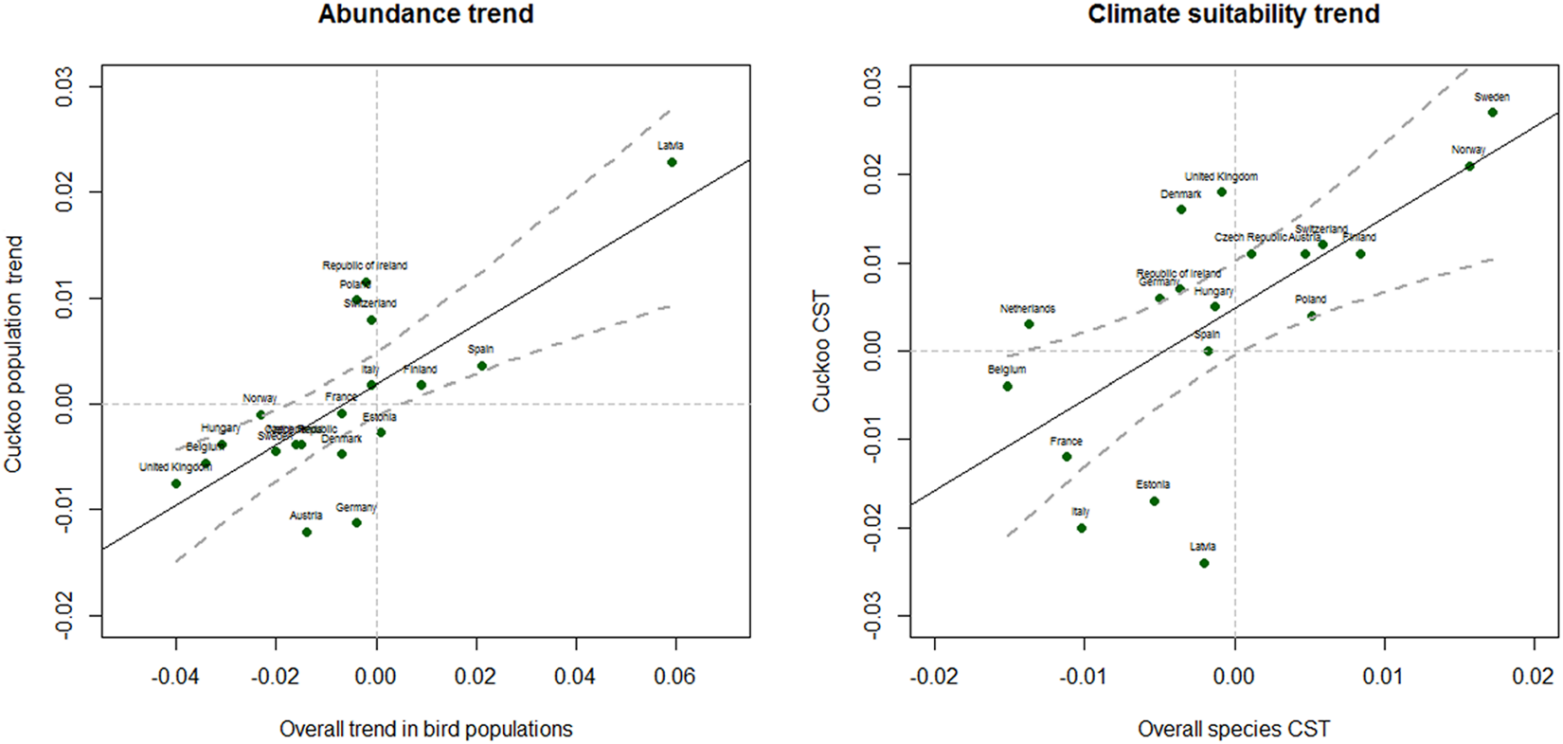
Correlation between cuckoo population trend and overall trend of bird populations (left panel) and cuckoo climate suitability trend (CST) and overall CST of birds (right panel) in ten European countries. The bird population trends and bird CST in Europe were obtained from Stephens *et al*.^14^. The lines are linear regression lines and 95% confidence intervals.

The temporal trend in common cuckoo populations was positively correlated with the overall trend in bird populations in European countries (r^2^ = 0.54, F = 19.9, df = 1, 17, p < 0.05; Table 2, Fig. 3). Finally, the climate suitability trend of the common cuckoo was positively correlated with the overall climate suitability trend for all other bird species in European countries (r^2^ = 0.43, F = 12.8, df = 1, 17, p < 0.05; Table 2, Fig. 3).

**Table 2:** Linear regression models based on data from European countries, accounting for cuckoo abundance trend and cuckoo climate suitability trend (CST) in relation to the same measeures for all bird populations in nineteen selected countries.

Predictors/Response	Estimate	CI	SE	Z	p
***Cuckoo abundance trend***					
(Intercept)	0.002	−0.001/0.005	0.001	1.268	0.221
Overall bird abundance trend	0.285	0.150/0.419	0.064	4.461	<0.05
***Cuckoo climate suitability trend***					
(Intercept)	0.005	−0.0005/0.010	0.002	1.918	0.072
Overall bird CST	1.032	0.423/1.641	0.288	3.576	<0.05

The bird population trends and bird climate suitability trend in Europe were taken from Stephens *et al*.^14^. The full models were based on 19 countries. CI: confidence interval (lower/upper); SE: standard error. Only significant variables are shown in the table.

## Discussion

### Cuckoo as indicator: Extending species surrogacy from Europe to Asia

This study provides new evidence on the common cuckoo as a surrogate of bird species richness, previously tested in some European countries. This suggests that the common cuckoo is potentially a prime bioindicator in Eurasia. Even if related to different host species, and considering that the common cuckoo is not the only parasitic cuckoo in Asia^24^, we found the same pattern than in Europe: Occurrence of the common cuckoo is positively correlated with bird species richness in both continents. The implications related to finding the same pattern in Europe and in Asia are important from an ecological point of view. First, in Asia, *C. canorus* has different host species than in Europe. Second, the common cuckoo is not the only brood parasite in China and Japan. So, *C. canorus* is subject to a greater competitive pressure from other cuckoo species in Asia than in Europe. However, common cuckoo still shows the same capacity as surrogate of bird species richness, highlighting the process linking this particular (and charismatic species) to overall bird diversity.

In fact, the performance of statistical models for common cuckoo was encouraging, providing predictive power from fair to excellent in studies based on point counts (by far the most commonly used in field ornithology^25, 26^) but acceptable also when working with other bird census approaches as transects or point count data merged in squares. These results confirm the hypothesis that this parasitic bird can be used to predict areas with high taxonomic diversity^11, 12^, providing new insights toward the identification of an adequate bioindicator across a wide spatial range, even using different survey methods. The study of spatial patterns of species richness is one of the most important components for designing reserves and ecological networks, as well as for predicting consequences of global change, and thus for conservation purposes^27^. The main explanation for the positive correlation between common cuckoo occurrence and high biodiversity is that the distribution of parasitic birds is driven not only by their own ecological needs (climate and trophic availability), but mainly by the presence of their host species^28, 29^. We have already demonstrated how the richness of host species is positively correlated with overall bird species richness^12^, and here we also demonstrated how the richness of even non-host species is positively correlated with overall bird species richness. Thus, areas characterized by high avian diversity are also areas with high values of cuckoo host diversity and host abundance^30^.

Our study shows that the population trend of common cuckoo, as well as climate suitability trend for the common cuckoo, follow the overall trend for populations of all other passerines species and the climate suitability trend in all Europena countries. This result supports the hypothesis that common cuckoo is a suitable bioindicator, making the species also sensible to climate change scenarios. When using proxies of population trends, many aspects need to be considered. For instance if within country variation in population abundance trends of different species is larger or smaller than variation among countries, and also how trends of other bird species can be related to the average community trend. However, in this study we adopted a descriptive approach rather than explaining the causal relationship between common cuckoo and overall trends^14^. The only intention was to test whether common cuckoo trend can reflect the trend of bird populations in general, and this test was affirmative. More studies are necessary, but our findings suggests that studying the trend of common cuckoo population can also predict the overall trend of bird species in a given community.

The occurrence of the common cuckoo could be sufficient for determining hotspots of bird taxonomic diversity. The hypothesis that the common cuckoo prefers specific habitats, and that the larger biodiversity in sites with cuckoos present simply reflects the presence of such habitats, is not supported by our results. In our extensive dataset, comprising twelve countries from two continents, we found the species in many different dominant environments, and even including in models the main type of environment, the surrogacy of the species was positively correlated with bird species richness. In fact, the common cuckoo in different parts of its range prefers meadows, shrub, parkland, open hedgerows, farmland or forests^31^.

### Cuckoo footprint in human culture, weakness and strengths of the proposed methodology and conservation implications

The common cuckoo still occupies notoriety in songs, literature^3, 32^, anatomy text books^6^ and popular culture, and it has attained iconic status in the cuckoo clock; the bird’s calls marking the passage of time, from hour to hour, the whole year round. Currently, new evidence suggests that common cuckoo can also play a role in conservation.

The proposed methodology represents a valid alternative to other ecological monitoring strategies, for many reasons. The main strengths are related to a) feasibility, b) cost-effectiveness and c) possibility of application in many kinds of environments.

Cuckoos are characterized by distinct and loud vocalisations, which greatly enhance the detectability of the species, increasing survey effectiveness for researchers. In fact, the well-recognized and widespread nature of the cuckoo in public consciousness, as well as the distinctiveness and popularity of its song^3, 12^, make the bird an effective tool for encouraging participation in wide-scale volunteer surveys^33, 34^. The advantages of using not only specialized ornithologists and citizens are the possibilities to collect more data at a large spatial scale, as well as directly involving citizens in an active role on conservation. The datasets collected by the general public (citizen science programs), can be used to improve biogeographical studies focused on large-scale conservation targets^35^. In the particular case of the common cuckoo, involvement of citizen science programs can provide large amounts of data, reducing the effective cost of surveys, without compromising the quality of data collected, which is one of the main criticisms raised against these kind of programmes^36^.

Even considering the main criteria for the definition of a good surrogate (balance between robustness, communicability, accuracy, generality, cost-effectiveness and good transferability of the surrogacy)^37^, in the light of our findings, the common cuckoo should constitute a useful tool capable of (a) identifying areas characterized by rich bird communities (hotspots of bird diversity); (b) being applicable in different environments, since the common cuckoo is a widespread species, well adapted to live in different habitats^31^; (c) being applicable to different survey methods (point counts, transects, square census, mapping, etc.) and (d) providing an efficient surrogate for biodiversity also under climate change scenarios.

We can hypothesize a potential weakness on the use of common cuckoo as surrogate of bird species richness in some wetlands, with high number of individuals of a preferred host species. For instance, we found high density populations of Oriental reed warblers *Acrocephalus orientalis*, one of the most common host for *Cuculus canorus* ^24^, in wetland areas inside some urban parks of Beijing, China^38^. There, the predictive power of cuckoo occurrence as a surrogate of bird species richness can be a little bit compromised, because more strongly correlated to the density of *Acrocephalus orientalis*.

However, because much of the work to conserve biodiversity is carried out by non-governmental conservation organizations with the help of public support, the use of an emblematic species, like the common cuckoo, that can be considered a flagship species, is expected to be a valuable tool for communication and for convincing the public, authorities and politicians to preserve and enhance biodiversity in different ecosystems^39^. Furthermore, as highlighted by Entwistle *et al*.^40^, even considering that charisma of a species is culturally dependent^41^, the effectiveness of a flagship species can be largely improved if the connection between the species and human culture is strong, as for the common cuckoo. The common cuckoo surrogacy represents such a case, where science can draw upon folklore and lead conservation planning by exploiting the iconic image and sound of a bird.

## Methods

The study was carried out using data on bird species presence-absence collected in ten European and two Asian countries, partly derived from our previous studies and partly from this new study (Table 3). A detailed description of the bird surveys performed in Czech Republic, France, Greece, Switzerland, San Marino and Italy is provided in Morelli *et al*.^12^. In Finland, Ukraine, Poland, Denmark, China and Japan data on bird species richness were collected using the same methodology (Table 3). All point counts were performed for 5 minutes, during favourable weather conditions. At each sample site we recorded the occurrence of the cuckoo, assuming a constant detectability of the species^42^, and all bird species detected acoustically or visually were recorded. Bird species richness was expressed as the number of bird species recorded during each point count. Bird species richness was used as a measure of taxonomic diversity in all countries^43^. Information about cuckoo’s host species in some European countries was taken from literature^10, 44^. When available, we also calculated the common cuckoo’s host-species richness, as the number of bird species potentially host of this brood parasite in each country.

**Table 3:** Summary of survey methodology, number of sample sites, rate of occurrence of common cuckoo and source of data (published or unpublished) for 12 countries where observations of common cuckoos were collected.

Country	Survey method	Sample sites	Rate presence/absence (%)	Source
Czech Republic	transect	101	35.6	12
France	square	1153	76.2	12
Finland	point count	158	63.9	This study
Greece	point count	285	12.0	12
Switzerland	point count	115	17.4	12
Ukraine	point count	258	50.4	This study
Poland	point count	332	50.0	This study
Japan	point count	400	2.2	This study
San Marino	point count	250	22.8	This study
Italy	point count	287	12.0	12
Denmark	point count	48	50.0	This study
China	point count	205	2.0	This study

All sample sites were classified as forest, farmland, grassland or urban during the survey, on the basis of the dominant category of land use 100 m around each sample site, when main land use was >50%^45^. Sample sites where no single land-use was dominant, were classified as mixed environments.

The abundance trend and climate suitability trend (CST) for all bird species were obtained from Stephens *et al*.^14^. For each bird species, the abundance trend is “the slope of the regression of the natural logarithm of the abundance index on calendar year across the time period encompassed by 1980 and 2010”^14^. The climate suitability trend is based on the response of bird species to climatic variables recorded in the same period as bird populations were estimated (1980–2010)^14^. The CST is, by definition, “the slope of the regression between logit annual mean probability of occurrence and year for each species, under the bioclimate variables measured for each year” (see detailed definition in ref. 46). CSTs can be considered as informative predictors of abundance trends, and they can then be used to study potential impacts of future climatic change^14, 46, 47^. For all European countries where the common cuckoo is present, we estimated the overall values of bird abundance trend and climate suitability trend, as the average values considering all bird species present in each country using the data provided in Stephens *et al*.^14^.

### Statistical analyses

The relationship between common cuckoo occurrence and bird species richness (taxonomic diversity) at the level of each sample site was examined using Generalized Linear Mixed Models (GLMMs). We used Mantel tests to test for spatial autocorrelation in the data^48, 49^, based on the geographic distance matrix and the matrix of differences in bird species richness among sites, applying Monte Carlo permutations with 999 randomizations to test for significance^50^. Bird species richness was modeled as a fixed effect^51^, while country and dominant environment (land use) were included as random effects to account for possible consistent differences among countries and environments. Dominant environment was included as a random effect because we were not interested in exploring the potential fixed effects on cuckoo distribution. The ecological rationale for these models is that common cuckoo occurrence is sensitive to variation in bird community composition^11, 12^. Therefore, cuckoo occurrence (presence-absence) was used as a binomial response variable, while bird species richness (numerical variable) was used as a predictor. Best-fit models were selected by lower Akaike Information Criterion (AIC) values^52^. The confidence intervals for the significant variables selected in the best model were calculated by the Wald method^51^. In order to estimate the accuracy of the models, we used the area under the ROC curve (AUC)^53^, which indicates the predictive performance expressed as an index ranging from 0.5 to 1^54^. The accuracy of the model can be interpreted after Swets^55^ as following: 0.90–1.00 excellent; 0.80–0.90 good; 0.70–0.80 fair; 0.60–0.70 poor; and 0.50–0.60 fail.

The correlation between bird species richness and non-host species richness, as well as the correlation between the abundance trend of common cuckoo populations or their climate suitability trend and the overall bird populations were analysed by means of linear regression. We compared the common cuckoo abundance trend and CST with overall values obtained for bird communities in each country, in order to understand if common cuckoo trends follow overall trends. All statistical tests were performed with R software^56^.

## Electronic supplementary material

Electronic Supplementary Material
