# Supplementary material for: The common cuckoo is an effective indicator of high bird species richness in Asia and Europe
Source: Sci Rep. 2017 Jun 29;7:4376. doi: 10.1038/s41598-017-04794-3 (PMC5491505; doi:10.1038/s41598-017-04794-3)
Supplement: Supplementary file 1 — Electronic Supplementary Material [file 41598_2017_4794_MOESM1_ESM.pdf]

Electronic Supplementary Material

The common cuckoo is an effective indicator of high bird species richness in Asia and Europe

Federico Morelli, Anders Pape Møller, Emma Nelson, Yanina Benedetti, Wei Liang, Petra Šímová, Marco Moretti, Piotr Tryjanowski

Tables

Table A. Bird species richness in areas with cuckoo was present and absent in ten European and two Asiatic countries.

	Bird species richness							
**Country**	**Cuckoo: absent**				**Cuckoo: present**			
	average	max	min	SD	average	max	min	SD
China	4.2	17	0	2.6	12.0	13	11	1.2
Czech Republic	41.0	63	19	9.0	47.1	62	23	9.7
Denmark	4.6	13	1	2.8	9.7	19	6	3.3
Finland	5.9	11	3	1.9	7.4	12	4	1.9
France	31.1	52	5	8.9	37.5	58	15	7.7
Greece	3.8	15	1	1.9	5.6	12	2	2.3
Italy	4.9	16	1	3.0	10.1	23	3	3.9
Japan	4.0	10	0	1.8	6.3	9	5	1.5
Poland	6.0	12	2	1.9	8.8	15	3	2.5
San Marino	4.7	22	1	5.3	13.5	24	5	6.1
Switzerland	15.9	23	10	3.0	19.1	28	15	3.8
Ukraine	4.4	10	1	1.8	6.5	15	2	2.2

Figures

Figure A. Goodness of fit measure (Area Under the Curve, AUC) of best models correlating cuckoo occurrence to bird species richness in ten European and two Asiatic countries.

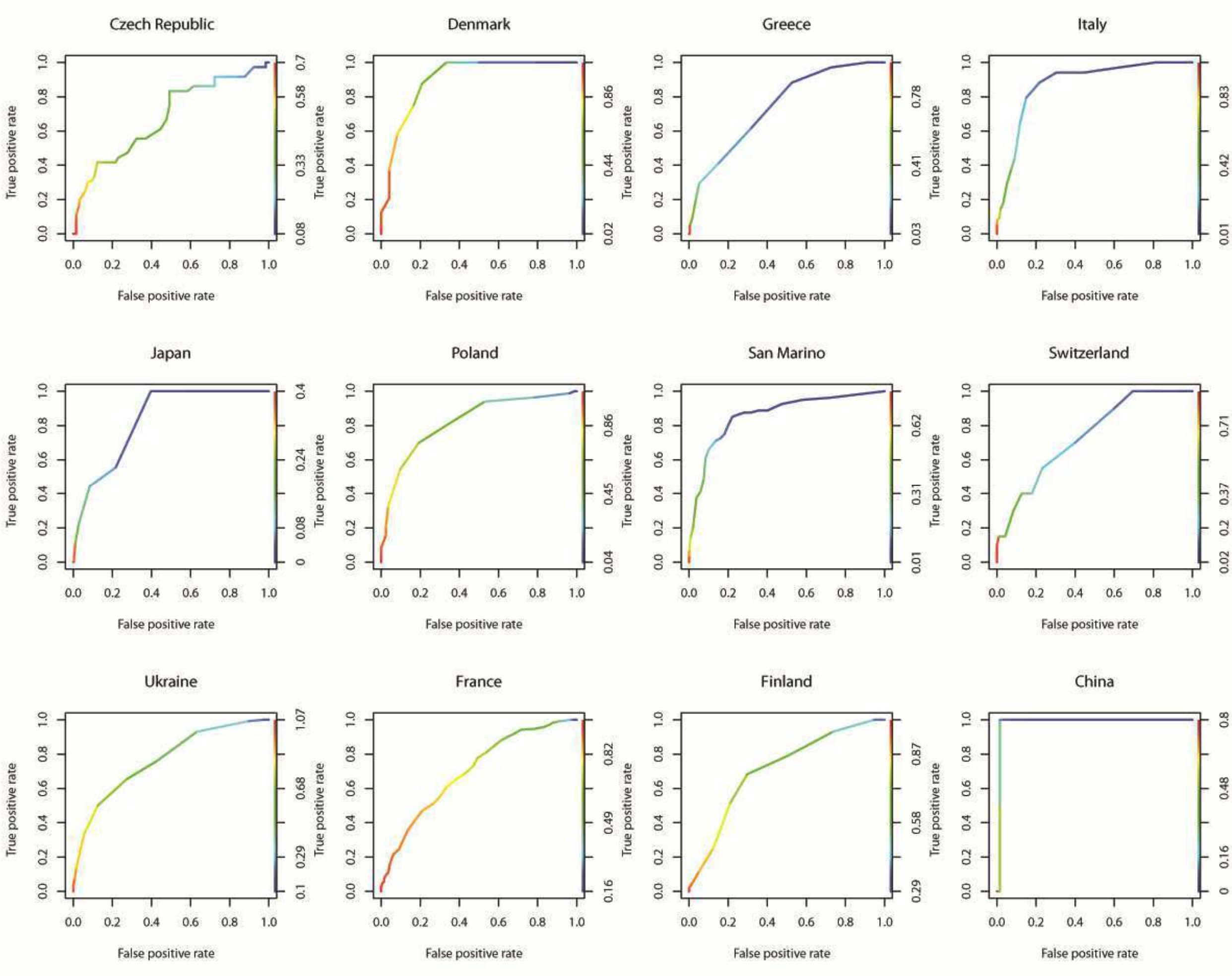

Electronic Supplementary Material - dataset

The common cuckoo is an effective indicator of high bird species richness in Asia and Europe

Federico Morelli, Anders Pape Møller, Emma Nelson, Yanina Benedetti, Wei Liang, Petra Šímová, Marco Moretti, Piotr Tryjanowski

ID	Country	Continent	Dominant environment	Cuckoo occurrence	Bird richness	Type of survey
1	Czech Republic	Europe	mixed	presence	57	transect
2	Czech Republic	Europe	mixed	absence	37	transect
3	Czech Republic	Europe	mixed	presence	62	transect
4	Czech Republic	Europe	mixed	absence	32	transect
5	Czech Republic	Europe	mixed	absence	40	transect
6	Czech Republic	Europe	mixed	absence	44	transect
7	Czech Republic	Europe	mixed	presence	58	transect
8	Czech Republic	Europe	mixed	presence	55	transect
9	Czech Republic	Europe	mixed	absence	56	transect
10	Czech Republic	Europe	mixed	presence	58	transect
11	Czech Republic	Europe	mixed	absence	36	transect
12	Czech Republic	Europe	mixed	absence	27	transect
13	Czech Republic	Europe	mixed	presence	39	transect
14	Czech Republic	Europe	mixed	presence	47	transect
15	Czech Republic	Europe	mixed	presence	44	transect
16	Czech Republic	Europe	mixed	absence	32	transect
17	Czech Republic	Europe	mixed	presence	30	transect
18	Czech Republic	Europe	mixed	absence	25	transect
19	Czech Republic	Europe	mixed	absence	30	transect
20	Czech Republic	Europe	mixed	presence	46	transect
21	Czech Republic	Europe	mixed	presence	42	transect
22	Czech Republic	Europe	mixed	absence	44	transect
23	Czech Republic	Europe	mixed	absence	43	transect
24	Czech Republic	Europe	mixed	presence	44	transect
25	Czech Republic	Europe	mixed	absence	31	transect
26	Czech Republic	Europe	mixed	presence	48	transect
27	Czech Republic	Europe	mixed	presence	43	transect
28	Czech Republic	Europe	mixed	presence	62	transect
29	Czech Republic	Europe	mixed	absence	47	transect
30	Czech Republic	Europe	mixed	absence	40	transect
31	Czech Republic	Europe	mixed	absence	46	transect
32	Czech Republic	Europe	mixed	absence	37	transect
33	Czech Republic	Europe	mixed	absence	52	transect
34	Czech Republic	Europe	mixed	absence	49	transect
35	Czech Republic	Europe	mixed	absence	45	transect
36	Czech Republic	Europe	mixed	presence	59	transect
37	Czech Republic	Europe	mixed	absence	42	transect
38	Czech Republic	Europe	mixed	absence	47	transect
39	Czech Republic	Europe	mixed	presence	46	transect
40	Czech Republic	Europe	mixed	absence	56	transect
41	Czech Republic	Europe	mixed	absence	36	transect
42	Czech Republic	Europe	mixed	absence	47	transect
43	Czech Republic	Europe	mixed	absence	50	transect
44	Czech Republic	Europe	mixed	presence	42	transect
45	Czech Republic	Europe	mixed	presence	35	transect
46	Czech Republic	Europe	mixed	absence	44	transect
47	Czech Republic	Europe	mixed	absence	32	transect
48	Czech Republic	Europe	mixed	absence	32	transect
49	Czech Republic	Europe	mixed	absence	50	transect

50	Czech Republic	Europe	mixed	absence	58	transect
51	Czech Republic	Europe	mixed	absence	54	transect
52	Czech Republic	Europe	mixed	absence	26	transect
53	Czech Republic	Europe	mixed	presence	41	transect
54	Czech Republic	Europe	mixed	presence	55	transect
55	Czech Republic	Europe	mixed	absence	30	transect
56	Czech Republic	Europe	mixed	presence	46	transect
57	Czech Republic	Europe	mixed	absence	30	transect
58	Czech Republic	Europe	mixed	absence	53	transect
59	Czech Republic	Europe	mixed	absence	63	transect
60	Czech Republic	Europe	mixed	presence	52	transect
61	Czech Republic	Europe	mixed	absence	43	transect
62	Czech Republic	Europe	mixed	presence	41	transect
63	Czech Republic	Europe	mixed	absence	34	transect
64	Czech Republic	Europe	mixed	absence	46	transect
65	Czech Republic	Europe	mixed	absence	33	transect
66	Czech Republic	Europe	mixed	absence	39	transect
67	Czech Republic	Europe	mixed	presence	42	transect
68	Czech Republic	Europe	mixed	presence	52	transect
69	Czech Republic	Europe	mixed	absence	38	transect
70	Czech Republic	Europe	mixed	absence	40	transect
71	Czech Republic	Europe	mixed	absence	45	transect
72	Czech Republic	Europe	mixed	absence	49	transect
73	Czech Republic	Europe	mixed	absence	55	transect
74	Czech Republic	Europe	mixed	absence	40	transect
75	Czech Republic	Europe	mixed	absence	34	transect
76	Czech Republic	Europe	mixed	absence	40	transect
77	Czech Republic	Europe	mixed	absence	31	transect
78	Czech Republic	Europe	mixed	absence	45	transect
79	Czech Republic	Europe	mixed	presence	53	transect
80	Czech Republic	Europe	mixed	absence	49	transect
81	Czech Republic	Europe	mixed	absence	49	transect
82	Czech Republic	Europe	mixed	absence	46	transect
83	Czech Republic	Europe	mixed	absence	39	transect
84	Czech Republic	Europe	mixed	absence	37	transect
85	Czech Republic	Europe	mixed	presence	56	transect
86	Czech Republic	Europe	mixed	absence	26	transect
87	Czech Republic	Europe	mixed	presence	23	transect
88	Czech Republic	Europe	mixed	presence	30	transect
89	Czech Republic	Europe	mixed	absence	38	transect
90	Czech Republic	Europe	mixed	absence	40	transect
91	Czech Republic	Europe	mixed	presence	61	transect
92	Czech Republic	Europe	mixed	presence	35	transect
93	Czech Republic	Europe	mixed	presence	52	transect
94	Czech Republic	Europe	mixed	absence	34	transect
95	Czech Republic	Europe	mixed	absence	19	transect
96	Czech Republic	Europe	mixed	presence	43	transect
97	Czech Republic	Europe	mixed	presence	41	transect
98	Czech Republic	Europe	mixed	absence	44	transect
99	Czech Republic	Europe	mixed	absence	48	transect

100	Czech Republic	Europe	mixed	presence	56	transect
101	Czech Republic	Europe	mixed	absence	44	transect
102	Denmark	Europe	farmland	presence	9	point count
103	Denmark	Europe	farmland	absence	13	point count
104	Denmark	Europe	farmland	presence	14	point count
105	Denmark	Europe	farmland	absence	9	point count
106	Denmark	Europe	farmland	presence	9	point count
107	Denmark	Europe	farmland	absence	8	point count
108	Denmark	Europe	farmland	presence	9	point count
109	Denmark	Europe	farmland	absence	8	point count
110	Denmark	Europe	farmland	presence	19	point count
111	Denmark	Europe	farmland	absence	6	point count
112	Denmark	Europe	farmland	presence	17	point count
113	Denmark	Europe	farmland	absence	5	point count
114	Denmark	Europe	farmland	presence	10	point count
115	Denmark	Europe	farmland	absence	2	point count
116	Denmark	Europe	farmland	presence	6	point count
117	Denmark	Europe	farmland	absence	2	point count
118	Denmark	Europe	farmland	presence	13	point count
119	Denmark	Europe	farmland	absence	4	point count
120	Denmark	Europe	farmland	presence	13	point count
121	Denmark	Europe	farmland	absence	3	point count
122	Denmark	Europe	farmland	presence	9	point count
123	Denmark	Europe	farmland	absence	1	point count
124	Denmark	Europe	farmland	presence	6	point count
125	Denmark	Europe	farmland	absence	2	point count
126	Denmark	Europe	farmland	presence	7	point count
127	Denmark	Europe	farmland	absence	6	point count
128	Denmark	Europe	farmland	presence	6	point count
129	Denmark	Europe	farmland	absence	4	point count
130	Denmark	Europe	farmland	presence	9	point count
131	Denmark	Europe	farmland	absence	2	point count
132	Denmark	Europe	farmland	presence	7	point count
133	Denmark	Europe	farmland	absence	5	point count
134	Denmark	Europe	farmland	presence	10	point count
135	Denmark	Europe	farmland	absence	6	point count
136	Denmark	Europe	farmland	presence	7	point count
137	Denmark	Europe	farmland	absence	3	point count
138	Denmark	Europe	farmland	presence	10	point count
139	Denmark	Europe	farmland	absence	3	point count
140	Denmark	Europe	farmland	presence	8	point count
141	Denmark	Europe	farmland	absence	7	point count
142	Denmark	Europe	farmland	presence	8	point count
143	Denmark	Europe	farmland	absence	3	point count
144	Denmark	Europe	farmland	presence	8	point count
145	Denmark	Europe	farmland	absence	3	point count
146	Denmark	Europe	farmland	presence	8	point count
147	Denmark	Europe	farmland	absence	3	point count
148	Denmark	Europe	farmland	presence	10	point count
149	Denmark	Europe	farmland	absence	3	point count

150	Greece	Europe	farmland	absence	3	point count
151	Greece	Europe	farmland	absence	1	point count
152	Greece	Europe	farmland	absence	3	point count
153	Greece	Europe	farmland	absence	3	point count
154	Greece	Europe	farmland	absence	3	point count
155	Greece	Europe	farmland	absence	1	point count
156	Greece	Europe	farmland	absence	2	point count
157	Greece	Europe	farmland	absence	3	point count
158	Greece	Europe	farmland	absence	5	point count
159	Greece	Europe	farmland	absence	2	point count
160	Greece	Europe	farmland	absence	4	point count
161	Greece	Europe	farmland	absence	3	point count
162	Greece	Europe	farmland	absence	4	point count
163	Greece	Europe	farmland	absence	5	point count
164	Greece	Europe	farmland	absence	3	point count
165	Greece	Europe	farmland	absence	1	point count
166	Greece	Europe	farmland	absence	5	point count
167	Greece	Europe	farmland	absence	5	point count
168	Greece	Europe	farmland	absence	4	point count
169	Greece	Europe	farmland	absence	4	point count
170	Greece	Europe	farmland	absence	3	point count
171	Greece	Europe	farmland	absence	3	point count
172	Greece	Europe	farmland	absence	3	point count
173	Greece	Europe	farmland	absence	1	point count
174	Greece	Europe	farmland	absence	5	point count
175	Greece	Europe	farmland	absence	5	point count
176	Greece	Europe	farmland	absence	2	point count
177	Greece	Europe	farmland	absence	5	point count
178	Greece	Europe	farmland	absence	4	point count
179	Greece	Europe	farmland	absence	3	point count
180	Greece	Europe	farmland	absence	2	point count
181	Greece	Europe	farmland	absence	2	point count
182	Greece	Europe	farmland	absence	2	point count
183	Greece	Europe	farmland	absence	4	point count
184	Greece	Europe	farmland	absence	3	point count
185	Greece	Europe	farmland	absence	6	point count
186	Greece	Europe	farmland	absence	3	point count
187	Greece	Europe	farmland	absence	4	point count
188	Greece	Europe	farmland	absence	5	point count
189	Greece	Europe	farmland	absence	3	point count
190	Greece	Europe	farmland	absence	3	point count
191	Greece	Europe	farmland	absence	2	point count
192	Greece	Europe	farmland	absence	4	point count
193	Greece	Europe	farmland	absence	3	point count
194	Greece	Europe	farmland	absence	3	point count
195	Greece	Europe	farmland	absence	2	point count
196	Greece	Europe	farmland	absence	4	point count
197	Greece	Europe	farmland	absence	2	point count
198	Greece	Europe	farmland	absence	5	point count
199	Greece	Europe	farmland	absence	4	point count

200	Greece	Europe	farmland	absence	3	point count
201	Greece	Europe	farmland	absence	5	point count
202	Greece	Europe	farmland	absence	4	point count
203	Greece	Europe	farmland	absence	5	point count
204	Greece	Europe	farmland	absence	2	point count
205	Greece	Europe	farmland	absence	4	point count
206	Greece	Europe	farmland	absence	3	point count
207	Greece	Europe	farmland	absence	4	point count
208	Greece	Europe	farmland	absence	1	point count
209	Greece	Europe	farmland	absence	1	point count
210	Greece	Europe	farmland	absence	1	point count
211	Greece	Europe	farmland	absence	4	point count
212	Greece	Europe	farmland	absence	3	point count
213	Greece	Europe	farmland	absence	4	point count
214	Greece	Europe	farmland	absence	2	point count
215	Greece	Europe	farmland	absence	4	point count
216	Greece	Europe	farmland	absence	2	point count
217	Greece	Europe	farmland	absence	1	point count
218	Greece	Europe	farmland	absence	3	point count
219	Greece	Europe	farmland	absence	1	point count
220	Greece	Europe	farmland	absence	3	point count
221	Greece	Europe	farmland	absence	2	point count
222	Greece	Europe	farmland	absence	4	point count
223	Greece	Europe	farmland	absence	2	point count
224	Greece	Europe	farmland	absence	1	point count
225	Greece	Europe	farmland	absence	3	point count
226	Greece	Europe	farmland	absence	2	point count
227	Greece	Europe	farmland	absence	1	point count
228	Greece	Europe	farmland	absence	2	point count
229	Greece	Europe	farmland	absence	1	point count
230	Greece	Europe	farmland	absence	1	point count
231	Greece	Europe	farmland	absence	3	point count
232	Greece	Europe	farmland	absence	2	point count
233	Greece	Europe	farmland	absence	5	point count
234	Greece	Europe	farmland	absence	4	point count
235	Greece	Europe	farmland	presence	3	point count
236	Greece	Europe	farmland	presence	3	point count
237	Greece	Europe	farmland	absence	1	point count
238	Greece	Europe	farmland	absence	2	point count
239	Greece	Europe	farmland	absence	3	point count
240	Greece	Europe	farmland	absence	6	point count
241	Greece	Europe	farmland	absence	1	point count
242	Greece	Europe	farmland	absence	3	point count
243	Greece	Europe	farmland	absence	2	point count
244	Greece	Europe	farmland	absence	2	point count
245	Greece	Europe	farmland	absence	3	point count
246	Greece	Europe	farmland	absence	2	point count
247	Greece	Europe	farmland	presence	4	point count
248	Greece	Europe	farmland	absence	3	point count
249	Greece	Europe	farmland	absence	1	point count

250	Greece	Europe	farmland	absence	3	point count
251	Greece	Europe	farmland	absence	2	point count
252	Greece	Europe	farmland	absence	4	point count
253	Greece	Europe	farmland	absence	3	point count
254	Greece	Europe	farmland	absence	2	point count
255	Greece	Europe	farmland	absence	1	point count
256	Greece	Europe	farmland	absence	4	point count
257	Greece	Europe	farmland	absence	3	point count
258	Greece	Europe	farmland	absence	4	point count
259	Greece	Europe	farmland	absence	4	point count
260	Greece	Europe	farmland	absence	2	point count
261	Greece	Europe	farmland	absence	4	point count
262	Greece	Europe	farmland	absence	6	point count
263	Greece	Europe	farmland	absence	3	point count
264	Greece	Europe	farmland	absence	4	point count
265	Greece	Europe	farmland	absence	5	point count
266	Greece	Europe	farmland	absence	6	point count
267	Greece	Europe	farmland	absence	4	point count
268	Greece	Europe	farmland	absence	3	point count
269	Greece	Europe	farmland	absence	4	point count
270	Greece	Europe	farmland	absence	4	point count
271	Greece	Europe	farmland	absence	4	point count
272	Greece	Europe	farmland	absence	4	point count
273	Greece	Europe	farmland	absence	4	point count
274	Greece	Europe	farmland	absence	5	point count
275	Greece	Europe	farmland	absence	4	point count
276	Greece	Europe	farmland	absence	2	point count
277	Greece	Europe	farmland	absence	5	point count
278	Greece	Europe	farmland	absence	2	point count
279	Greece	Europe	farmland	absence	2	point count
280	Greece	Europe	farmland	absence	2	point count
281	Greece	Europe	farmland	absence	1	point count
282	Greece	Europe	farmland	absence	9	point count
283	Greece	Europe	farmland	absence	3	point count
284	Greece	Europe	farmland	absence	8	point count
285	Greece	Europe	farmland	absence	5	point count
286	Greece	Europe	farmland	absence	5	point count
287	Greece	Europe	farmland	absence	4	point count
288	Greece	Europe	farmland	absence	3	point count
289	Greece	Europe	farmland	absence	5	point count
290	Greece	Europe	farmland	absence	3	point count
291	Greece	Europe	farmland	absence	4	point count
292	Greece	Europe	farmland	absence	7	point count
293	Greece	Europe	farmland	absence	9	point count
294	Greece	Europe	farmland	absence	3	point count
295	Greece	Europe	farmland	absence	5	point count
296	Greece	Europe	farmland	absence	6	point count
297	Greece	Europe	farmland	absence	6	point count
298	Greece	Europe	farmland	absence	3	point count
299	Greece	Europe	farmland	absence	4	point count

300	Greece	Europe	farmland	absence	5	point count
301	Greece	Europe	farmland	absence	6	point count
302	Greece	Europe	farmland	absence	5	point count
303	Greece	Europe	farmland	absence	7	point count
304	Greece	Europe	farmland	absence	6	point count
305	Greece	Europe	farmland	absence	8	point count
306	Greece	Europe	farmland	absence	2	point count
307	Greece	Europe	farmland	absence	6	point count
308	Greece	Europe	farmland	absence	2	point count
309	Greece	Europe	farmland	absence	2	point count
310	Greece	Europe	farmland	absence	2	point count
311	Greece	Europe	farmland	absence	4	point count
312	Greece	Europe	farmland	absence	6	point count
313	Greece	Europe	farmland	absence	5	point count
314	Greece	Europe	farmland	absence	3	point count
315	Greece	Europe	farmland	absence	4	point count
316	Greece	Europe	farmland	absence	5	point count
317	Greece	Europe	farmland	absence	5	point count
318	Greece	Europe	farmland	absence	6	point count
319	Greece	Europe	farmland	absence	15	point count
320	Greece	Europe	farmland	absence	5	point count
321	Greece	Europe	farmland	absence	5	point count
322	Greece	Europe	farmland	absence	6	point count
323	Greece	Europe	farmland	absence	6	point count
324	Greece	Europe	farmland	absence	6	point count
325	Greece	Europe	farmland	absence	5	point count
326	Greece	Europe	farmland	absence	5	point count
327	Greece	Europe	farmland	absence	7	point count
328	Greece	Europe	farmland	absence	5	point count
329	Greece	Europe	farmland	absence	6	point count
330	Greece	Europe	farmland	absence	4	point count
331	Greece	Europe	farmland	absence	5	point count
332	Greece	Europe	farmland	absence	6	point count
333	Greece	Europe	farmland	absence	5	point count
334	Greece	Europe	farmland	absence	5	point count
335	Greece	Europe	farmland	absence	6	point count
336	Greece	Europe	farmland	absence	2	point count
337	Greece	Europe	farmland	absence	6	point count
338	Greece	Europe	farmland	absence	4	point count
339	Greece	Europe	farmland	absence	3	point count
340	Greece	Europe	farmland	absence	8	point count
341	Greece	Europe	farmland	absence	3	point count
342	Greece	Europe	farmland	absence	4	point count
343	Greece	Europe	farmland	absence	4	point count
344	Greece	Europe	farmland	absence	2	point count
345	Greece	Europe	farmland	absence	4	point count
346	Greece	Europe	farmland	presence	2	point count
347	Greece	Europe	farmland	presence	3	point count
348	Greece	Europe	farmland	presence	5	point count
349	Greece	Europe	farmland	absence	2	point count

350	Greece	Europe	farmland	presence	4	point count
351	Greece	Europe	farmland	absence	1	point count
352	Greece	Europe	farmland	absence	3	point count
353	Greece	Europe	farmland	absence	2	point count
354	Greece	Europe	farmland	absence	1	point count
355	Greece	Europe	farmland	absence	1	point count
356	Greece	Europe	farmland	absence	3	point count
357	Greece	Europe	farmland	presence	5	point count
358	Greece	Europe	farmland	absence	4	point count
359	Greece	Europe	farmland	absence	5	point count
360	Greece	Europe	farmland	absence	6	point count
361	Greece	Europe	farmland	presence	6	point count
362	Greece	Europe	farmland	presence	7	point count
363	Greece	Europe	farmland	presence	5	point count
364	Greece	Europe	farmland	presence	8	point count
365	Greece	Europe	farmland	absence	5	point count
366	Greece	Europe	farmland	absence	4	point count
367	Greece	Europe	farmland	presence	6	point count
368	Greece	Europe	farmland	presence	4	point count
369	Greece	Europe	farmland	presence	4	point count
370	Greece	Europe	farmland	absence	5	point count
371	Greece	Europe	farmland	absence	4	point count
372	Greece	Europe	farmland	absence	3	point count
373	Greece	Europe	farmland	absence	5	point count
374	Greece	Europe	farmland	absence	6	point count
375	Greece	Europe	farmland	absence	3	point count
376	Greece	Europe	farmland	absence	2	point count
377	Greece	Europe	farmland	absence	3	point count
378	Greece	Europe	farmland	absence	3	point count
379	Greece	Europe	farmland	absence	5	point count
380	Greece	Europe	farmland	absence	6	point count
381	Greece	Europe	farmland	absence	4	point count
382	Greece	Europe	farmland	absence	4	point count
383	Greece	Europe	farmland	absence	2	point count
384	Greece	Europe	farmland	absence	2	point count
385	Greece	Europe	farmland	absence	2	point count
386	Greece	Europe	farmland	absence	2	point count
387	Greece	Europe	farmland	absence	3	point count
388	Greece	Europe	farmland	absence	1	point count
389	Greece	Europe	farmland	absence	4	point count
390	Greece	Europe	farmland	absence	7	point count
391	Greece	Europe	farmland	absence	1	point count
392	Greece	Europe	farmland	absence	2	point count
393	Greece	Europe	farmland	absence	3	point count
394	Greece	Europe	farmland	absence	2	point count
395	Greece	Europe	farmland	absence	6	point count
396	Greece	Europe	farmland	absence	5	point count
397	Greece	Europe	farmland	absence	4	point count
398	Greece	Europe	farmland	absence	8	point count
399	Greece	Europe	farmland	absence	6	point count

400	Greece	Europe	farmland	absence	5	point count
401	Greece	Europe	farmland	absence	5	point count
402	Greece	Europe	farmland	absence	6	point count
403	Greece	Europe	farmland	absence	2	point count
404	Greece	Europe	farmland	absence	2	point count
405	Greece	Europe	farmland	presence	12	point count
406	Greece	Europe	farmland	presence	8	point count
407	Greece	Europe	farmland	presence	9	point count
408	Greece	Europe	farmland	presence	6	point count
409	Greece	Europe	farmland	presence	6	point count
410	Greece	Europe	farmland	presence	7	point count
411	Greece	Europe	farmland	absence	4	point count
412	Greece	Europe	farmland	presence	5	point count
413	Greece	Europe	farmland	presence	4	point count
414	Greece	Europe	farmland	presence	4	point count
415	Greece	Europe	farmland	presence	7	point count
416	Greece	Europe	farmland	absence	10	point count
417	Greece	Europe	farmland	presence	8	point count
418	Greece	Europe	farmland	absence	6	point count
419	Greece	Europe	farmland	presence	12	point count
420	Greece	Europe	farmland	absence	2	point count
421	Greece	Europe	farmland	presence	4	point count
422	Greece	Europe	farmland	presence	5	point count
423	Greece	Europe	farmland	presence	4	point count
424	Greece	Europe	farmland	absence	4	point count
425	Greece	Europe	farmland	presence	8	point count
426	Greece	Europe	farmland	presence	4	point count
427	Greece	Europe	farmland	absence	5	point count
428	Greece	Europe	farmland	absence	8	point count
429	Greece	Europe	farmland	absence	3	point count
430	Greece	Europe	farmland	absence	5	point count
431	Greece	Europe	farmland	absence	4	point count
432	Greece	Europe	farmland	absence	4	point count
433	Greece	Europe	farmland	presence	5	point count
434	Greece	Europe	farmland	presence	5	point count
435	Italy	Europe	grassland	absence	8	point count
436	Italy	Europe	grassland	absence	7	point count
437	Italy	Europe	grassland	absence	14	point count
438	Italy	Europe	grassland	absence	11	point count
439	Italy	Europe	grassland	absence	13	point count
440	Italy	Europe	grassland	absence	8	point count
441	Italy	Europe	grassland	absence	12	point count
442	Italy	Europe	grassland	presence	7	point count
443	Italy	Europe	grassland	absence	10	point count
444	Italy	Europe	grassland	absence	3	point count
445	Italy	Europe	grassland	absence	3	point count
446	Italy	Europe	grassland	absence	3	point count
447	Italy	Europe	grassland	absence	5	point count
448	Italy	Europe	grassland	absence	5	point count
449	Italy	Europe	grassland	absence	2	point count

450	Italy	Europe	grassland	absence	3	point count
451	Italy	Europe	grassland	absence	3	point count
452	Italy	Europe	grassland	absence	1	point count
453	Italy	Europe	grassland	absence	2	point count
454	Italy	Europe	grassland	absence	3	point count
455	Italy	Europe	grassland	absence	2	point count
456	Italy	Europe	grassland	absence	2	point count
457	Italy	Europe	grassland	absence	3	point count
458	Italy	Europe	grassland	absence	4	point count
459	Italy	Europe	grassland	absence	5	point count
460	Italy	Europe	grassland	absence	2	point count
461	Italy	Europe	grassland	absence	4	point count
462	Italy	Europe	grassland	absence	5	point count
463	Italy	Europe	grassland	absence	2	point count
464	Italy	Europe	grassland	absence	3	point count
465	Italy	Europe	grassland	absence	3	point count
466	Italy	Europe	grassland	absence	1	point count
467	Italy	Europe	grassland	absence	2	point count
468	Italy	Europe	grassland	absence	6	point count
469	Italy	Europe	grassland	absence	4	point count
470	Italy	Europe	grassland	absence	2	point count
471	Italy	Europe	grassland	absence	2	point count
472	Italy	Europe	grassland	absence	3	point count
473	Italy	Europe	grassland	absence	3	point count
474	Italy	Europe	grassland	absence	8	point count
475	Italy	Europe	grassland	absence	7	point count
476	Italy	Europe	grassland	absence	9	point count
477	Italy	Europe	grassland	absence	4	point count
478	Italy	Europe	grassland	absence	1	point count
479	Italy	Europe	grassland	absence	5	point count
480	Italy	Europe	grassland	absence	3	point count
481	Italy	Europe	grassland	absence	7	point count
482	Italy	Europe	grassland	absence	5	point count
483	Italy	Europe	grassland	absence	2	point count
484	Italy	Europe	grassland	absence	5	point count
485	Italy	Europe	grassland	absence	4	point count
486	Italy	Europe	grassland	absence	1	point count
487	Italy	Europe	grassland	absence	3	point count
488	Italy	Europe	grassland	absence	5	point count
489	Italy	Europe	grassland	absence	3	point count
490	Italy	Europe	grassland	absence	1	point count
491	Italy	Europe	grassland	absence	2	point count
492	Italy	Europe	grassland	absence	4	point count
493	Italy	Europe	grassland	absence	5	point count
494	Italy	Europe	grassland	absence	3	point count
495	Italy	Europe	grassland	absence	4	point count
496	Italy	Europe	grassland	absence	6	point count
497	Italy	Europe	grassland	absence	2	point count
498	Italy	Europe	grassland	absence	4	point count
499	Italy	Europe	grassland	absence	4	point count

500	Italy	Europe	grassland	absence	2	point count
501	Italy	Europe	grassland	absence	5	point count
502	Italy	Europe	grassland	absence	5	point count
503	Italy	Europe	grassland	absence	4	point count
504	Italy	Europe	grassland	absence	4	point count
505	Italy	Europe	grassland	absence	4	point count
506	Italy	Europe	grassland	absence	5	point count
507	Italy	Europe	grassland	absence	3	point count
508	Italy	Europe	grassland	absence	3	point count
509	Italy	Europe	grassland	absence	2	point count
510	Italy	Europe	grassland	absence	3	point count
511	Italy	Europe	grassland	absence	4	point count
512	Italy	Europe	grassland	absence	2	point count
513	Italy	Europe	grassland	absence	3	point count
514	Italy	Europe	grassland	absence	3	point count
515	Italy	Europe	grassland	absence	4	point count
516	Italy	Europe	grassland	absence	2	point count
517	Italy	Europe	grassland	absence	1	point count
518	Italy	Europe	grassland	absence	2	point count
519	Italy	Europe	grassland	absence	4	point count
520	Italy	Europe	grassland	absence	7	point count
521	Italy	Europe	grassland	absence	1	point count
522	Italy	Europe	grassland	absence	1	point count
523	Italy	Europe	grassland	absence	5	point count
524	Italy	Europe	grassland	absence	6	point count
525	Italy	Europe	grassland	absence	1	point count
526	Italy	Europe	grassland	absence	5	point count
527	Italy	Europe	grassland	absence	4	point count
528	Italy	Europe	grassland	absence	3	point count
529	Italy	Europe	grassland	absence	4	point count
530	Italy	Europe	grassland	absence	3	point count
531	Italy	Europe	grassland	absence	6	point count
532	Italy	Europe	grassland	absence	3	point count
533	Italy	Europe	grassland	absence	7	point count
534	Italy	Europe	grassland	absence	3	point count
535	Italy	Europe	grassland	absence	6	point count
536	Italy	Europe	grassland	absence	4	point count
537	Italy	Europe	grassland	absence	6	point count
538	Italy	Europe	grassland	absence	6	point count
539	Italy	Europe	grassland	absence	4	point count
540	Italy	Europe	grassland	absence	5	point count
541	Italy	Europe	grassland	absence	3	point count
542	Italy	Europe	grassland	absence	4	point count
543	Italy	Europe	grassland	absence	3	point count
544	Italy	Europe	grassland	absence	2	point count
545	Italy	Europe	grassland	absence	4	point count
546	Italy	Europe	grassland	absence	6	point count
547	Italy	Europe	grassland	absence	1	point count
548	Italy	Europe	grassland	absence	6	point count
549	Italy	Europe	grassland	absence	3	point count

550	Italy	Europe	grassland	absence	2	point count
551	Italy	Europe	grassland	absence	4	point count
552	Italy	Europe	grassland	absence	4	point count
553	Italy	Europe	grassland	absence	4	point count
554	Italy	Europe	grassland	absence	4	point count
555	Italy	Europe	grassland	absence	2	point count
556	Italy	Europe	grassland	absence	3	point count
557	Italy	Europe	grassland	absence	3	point count
558	Italy	Europe	grassland	absence	5	point count
559	Italy	Europe	grassland	absence	7	point count
560	Italy	Europe	grassland	absence	5	point count
561	Italy	Europe	grassland	absence	7	point count
562	Italy	Europe	grassland	absence	3	point count
563	Italy	Europe	grassland	absence	1	point count
564	Italy	Europe	grassland	absence	2	point count
565	Italy	Europe	grassland	absence	2	point count
566	Italy	Europe	grassland	absence	2	point count
567	Italy	Europe	grassland	absence	4	point count
568	Italy	Europe	grassland	absence	4	point count
569	Italy	Europe	grassland	absence	2	point count
570	Italy	Europe	grassland	absence	6	point count
571	Italy	Europe	grassland	absence	4	point count
572	Italy	Europe	grassland	absence	4	point count
573	Italy	Europe	grassland	absence	5	point count
574	Italy	Europe	grassland	absence	9	point count
575	Italy	Europe	grassland	absence	5	point count
576	Italy	Europe	grassland	absence	2	point count
577	Italy	Europe	grassland	absence	7	point count
578	Italy	Europe	grassland	absence	2	point count
579	Italy	Europe	grassland	absence	7	point count
580	Italy	Europe	grassland	absence	5	point count
581	Italy	Europe	grassland	absence	3	point count
582	Italy	Europe	grassland	absence	3	point count
583	Italy	Europe	grassland	absence	4	point count
584	Italy	Europe	grassland	absence	2	point count
585	Italy	Europe	grassland	absence	7	point count
586	Italy	Europe	grassland	absence	5	point count
587	Italy	Europe	grassland	absence	6	point count
588	Italy	Europe	grassland	absence	4	point count
589	Italy	Europe	grassland	absence	6	point count
590	Italy	Europe	grassland	absence	3	point count
591	Italy	Europe	grassland	absence	2	point count
592	Italy	Europe	grassland	absence	2	point count
593	Italy	Europe	grassland	absence	2	point count
594	Italy	Europe	grassland	absence	3	point count
595	Italy	Europe	grassland	absence	4	point count
596	Italy	Europe	grassland	absence	4	point count
597	Italy	Europe	grassland	absence	3	point count
598	Italy	Europe	grassland	absence	8	point count
599	Italy	Europe	grassland	absence	5	point count

600	Italy	Europe	grassland	absence	3	point count
601	Italy	Europe	grassland	absence	5	point count
602	Italy	Europe	grassland	absence	3	point count
603	Italy	Europe	grassland	absence	2	point count
604	Italy	Europe	grassland	absence	5	point count
605	Italy	Europe	grassland	absence	4	point count
606	Italy	Europe	grassland	absence	3	point count
607	Italy	Europe	grassland	absence	4	point count
608	Italy	Europe	grassland	absence	6	point count
609	Italy	Europe	grassland	absence	6	point count
610	Italy	Europe	grassland	absence	5	point count
611	Italy	Europe	grassland	absence	5	point count
612	Italy	Europe	grassland	absence	6	point count
613	Italy	Europe	grassland	absence	6	point count
614	Italy	Europe	grassland	absence	7	point count
615	Italy	Europe	grassland	absence	5	point count
616	Italy	Europe	grassland	absence	5	point count
617	Italy	Europe	grassland	absence	4	point count
618	Italy	Europe	grassland	absence	2	point count
619	Italy	Europe	grassland	absence	4	point count
620	Italy	Europe	grassland	absence	3	point count
621	Italy	Europe	grassland	absence	3	point count
622	Italy	Europe	grassland	absence	6	point count
623	Italy	Europe	grassland	absence	2	point count
624	Italy	Europe	grassland	absence	2	point count
625	Italy	Europe	grassland	absence	7	point count
626	Italy	Europe	grassland	absence	5	point count
627	Italy	Europe	grassland	absence	3	point count
628	Italy	Europe	grassland	absence	5	point count
629	Italy	Europe	grassland	absence	3	point count
630	Italy	Europe	grassland	absence	3	point count
631	Italy	Europe	grassland	absence	3	point count
632	Italy	Europe	grassland	absence	1	point count
633	Italy	Europe	grassland	absence	3	point count
634	Italy	Europe	grassland	absence	6	point count
635	Italy	Europe	grassland	absence	4	point count
636	Italy	Europe	grassland	absence	2	point count
637	Italy	Europe	grassland	absence	4	point count
638	Italy	Europe	grassland	absence	4	point count
639	Italy	Europe	grassland	absence	3	point count
640	Italy	Europe	grassland	absence	4	point count
641	Italy	Europe	grassland	absence	5	point count
642	Italy	Europe	grassland	absence	5	point count
643	Italy	Europe	grassland	absence	4	point count
644	Italy	Europe	grassland	absence	9	point count
645	Italy	Europe	grassland	absence	8	point count
646	Italy	Europe	grassland	absence	2	point count
647	Italy	Europe	grassland	absence	5	point count
648	Italy	Europe	grassland	absence	6	point count
649	Italy	Europe	grassland	absence	15	point count

650	Italy	Europe	grassland	absence	16	point count
651	Italy	Europe	grassland	absence	12	point count
652	Italy	Europe	grassland	absence	11	point count
653	Italy	Europe	grassland	absence	8	point count
654	Italy	Europe	grassland	absence	10	point count
655	Italy	Europe	grassland	presence	10	point count
656	Italy	Europe	grassland	presence	12	point count
657	Italy	Europe	grassland	absence	7	point count
658	Italy	Europe	grassland	presence	8	point count
659	Italy	Europe	grassland	presence	12	point count
660	Italy	Europe	grassland	absence	6	point count
661	Italy	Europe	grassland	presence	13	point count
662	Italy	Europe	grassland	absence	9	point count
663	Italy	Europe	grassland	absence	10	point count
664	Italy	Europe	grassland	absence	12	point count
665	Italy	Europe	grassland	presence	8	point count
666	Italy	Europe	grassland	absence	4	point count
667	Italy	Europe	grassland	absence	6	point count
668	Italy	Europe	grassland	absence	7	point count
669	Italy	Europe	grassland	absence	7	point count
670	Italy	Europe	grassland	absence	11	point count
671	Italy	Europe	grassland	absence	11	point count
672	Italy	Europe	grassland	presence	6	point count
673	Italy	Europe	grassland	absence	5	point count
674	Italy	Europe	grassland	presence	6	point count
675	Italy	Europe	grassland	presence	11	point count
676	Italy	Europe	grassland	presence	9	point count
677	Italy	Europe	grassland	presence	10	point count
678	Italy	Europe	grassland	presence	9	point count
679	Italy	Europe	grassland	absence	5	point count
680	Italy	Europe	grassland	absence	11	point count
681	Italy	Europe	grassland	presence	9	point count
682	Italy	Europe	grassland	presence	7	point count
683	Italy	Europe	grassland	presence	3	point count
684	Italy	Europe	grassland	presence	4	point count
685	Italy	Europe	grassland	absence	4	point count
686	Italy	Europe	grassland	presence	8	point count
687	Italy	Europe	grassland	presence	9	point count
688	Italy	Europe	grassland	absence	4	point count
689	Italy	Europe	grassland	absence	15	point count
690	Italy	Europe	grassland	absence	12	point count
691	Italy	Europe	grassland	presence	9	point count
692	Italy	Europe	grassland	presence	18	point count
693	Italy	Europe	grassland	absence	9	point count
694	Italy	Europe	grassland	absence	9	point count
695	Italy	Europe	grassland	absence	13	point count
696	Italy	Europe	grassland	absence	13	point count
697	Italy	Europe	grassland	absence	11	point count
698	Italy	Europe	grassland	presence	23	point count
699	Italy	Europe	grassland	absence	10	point count

700	Italy	Europe	grassland	presence	11	point count
701	Italy	Europe	grassland	presence	11	point count
702	Italy	Europe	grassland	absence	12	point count
703	Italy	Europe	grassland	presence	14	point count
704	Italy	Europe	grassland	absence	7	point count
705	Italy	Europe	grassland	presence	8	point count
706	Italy	Europe	grassland	presence	12	point count
707	Italy	Europe	grassland	presence	16	point count
708	Italy	Europe	grassland	presence	14	point count
709	Italy	Europe	grassland	presence	9	point count
710	Italy	Europe	grassland	absence	14	point count
711	Italy	Europe	grassland	presence	8	point count
712	Italy	Europe	grassland	absence	8	point count
713	Italy	Europe	grassland	presence	7	point count
714	Italy	Europe	grassland	presence	12	point count
715	Italy	Europe	grassland	absence	8	point count
716	Italy	Europe	grassland	absence	5	point count
717	Italy	Europe	grassland	absence	3	point count
718	Italy	Europe	grassland	absence	7	point count
719	Italy	Europe	grassland	absence	6	point count
720	Italy	Europe	grassland	presence	9	point count
721	Italy	Europe	grassland	absence	9	point count
722	Japan	Asia	NA	absence	5	point count
723	Japan	Asia	NA	absence	4	point count
724	Japan	Asia	NA	absence	5	point count
725	Japan	Asia	NA	absence	4	point count
726	Japan	Asia	NA	absence	7	point count
727	Japan	Asia	NA	absence	6	point count
728	Japan	Asia	NA	absence	3	point count
729	Japan	Asia	NA	absence	3	point count
730	Japan	Asia	NA	absence	5	point count
731	Japan	Asia	NA	absence	5	point count
732	Japan	Asia	NA	absence	6	point count
733	Japan	Asia	NA	absence	6	point count
734	Japan	Asia	NA	presence	5	point count
735	Japan	Asia	NA	presence	6	point count
736	Japan	Asia	NA	absence	5	point count
737	Japan	Asia	NA	presence	7	point count
738	Japan	Asia	NA	presence	7	point count
739	Japan	Asia	NA	absence	8	point count
740	Japan	Asia	NA	absence	7	point count
741	Japan	Asia	NA	absence	5	point count
742	Japan	Asia	NA	absence	7	point count
743	Japan	Asia	NA	absence	5	point count
744	Japan	Asia	NA	presence	5	point count
745	Japan	Asia	NA	absence	7	point count
746	Japan	Asia	NA	absence	5	point count
747	Japan	Asia	NA	presence	8	point count
748	Japan	Asia	NA	presence	5	point count
749	Japan	Asia	NA	absence	4	point count

750	Japan	Asia	NA	absence	5	point count
751	Japan	Asia	NA	absence	2	point count
752	Japan	Asia	NA	absence	5	point count
753	Japan	Asia	NA	absence	5	point count
754	Japan	Asia	NA	absence	2	point count
755	Japan	Asia	NA	absence	6	point count
756	Japan	Asia	NA	absence	4	point count
757	Japan	Asia	NA	absence	3	point count
758	Japan	Asia	NA	absence	2	point count
759	Japan	Asia	NA	absence	3	point count
760	Japan	Asia	NA	absence	5	point count
761	Japan	Asia	NA	absence	1	point count
762	Japan	Asia	NA	absence	3	point count
763	Japan	Asia	NA	absence	1	point count
764	Japan	Asia	NA	absence	4	point count
765	Japan	Asia	NA	absence	6	point count
766	Japan	Asia	NA	absence	4	point count
767	Japan	Asia	NA	absence	4	point count
768	Japan	Asia	NA	absence	6	point count
769	Japan	Asia	NA	presence	5	point count
770	Japan	Asia	NA	presence	9	point count
771	Japan	Asia	NA	absence	5	point count
772	Japan	Asia	NA	absence	2	point count
773	Japan	Asia	NA	absence	4	point count
774	Japan	Asia	NA	absence	5	point count
775	Japan	Asia	NA	absence	2	point count
776	Japan	Asia	NA	absence	3	point count
777	Japan	Asia	NA	absence	2	point count
778	Japan	Asia	NA	absence	1	point count
779	Japan	Asia	NA	absence	3	point count
780	Japan	Asia	NA	absence	3	point count
781	Japan	Asia	NA	absence	1	point count
782	Japan	Asia	NA	absence	2	point count
783	Japan	Asia	NA	absence	2	point count
784	Japan	Asia	NA	absence	3	point count
785	Japan	Asia	NA	absence	2	point count
786	Japan	Asia	NA	absence	2	point count
787	Japan	Asia	NA	absence	4	point count
788	Japan	Asia	NA	absence	1	point count
789	Japan	Asia	NA	absence	2	point count
790	Japan	Asia	NA	absence	2	point count
791	Japan	Asia	NA	absence	4	point count
792	Japan	Asia	NA	absence	2	point count
793	Japan	Asia	NA	absence	2	point count
794	Japan	Asia	NA	absence	4	point count
795	Japan	Asia	NA	absence	3	point count
796	Japan	Asia	NA	absence	3	point count
797	Japan	Asia	NA	absence	2	point count
798	Japan	Asia	NA	absence	2	point count
799	Japan	Asia	NA	absence	3	point count

800	Japan	Asia	NA	absence	3	point count
801	Japan	Asia	NA	absence	5	point count
802	Japan	Asia	NA	absence	4	point count
803	Japan	Asia	NA	absence	3	point count
804	Japan	Asia	NA	absence	2	point count
805	Japan	Asia	NA	absence	3	point count
806	Japan	Asia	NA	absence	2	point count
807	Japan	Asia	NA	absence	2	point count
808	Japan	Asia	NA	absence	2	point count
809	Japan	Asia	NA	absence	6	point count
810	Japan	Asia	NA	absence	3	point count
811	Japan	Asia	NA	absence	4	point count
812	Japan	Asia	NA	absence	4	point count
813	Japan	Asia	NA	absence	2	point count
814	Japan	Asia	NA	absence	2	point count
815	Japan	Asia	NA	absence	4	point count
816	Japan	Asia	NA	absence	2	point count
817	Japan	Asia	NA	absence	5	point count
818	Japan	Asia	NA	absence	3	point count
819	Japan	Asia	NA	absence	1	point count
820	Japan	Asia	NA	absence	4	point count
821	Japan	Asia	NA	absence	5	point count
822	Japan	Asia	NA	absence	5	point count
823	Japan	Asia	NA	absence	4	point count
824	Japan	Asia	NA	absence	6	point count
825	Japan	Asia	NA	absence	6	point count
826	Japan	Asia	NA	absence	7	point count
827	Japan	Asia	NA	absence	7	point count
828	Japan	Asia	NA	absence	8	point count
829	Japan	Asia	NA	absence	5	point count
830	Japan	Asia	NA	absence	2	point count
831	Japan	Asia	NA	absence	3	point count
832	Japan	Asia	NA	absence	3	point count
833	Japan	Asia	NA	absence	5	point count
834	Japan	Asia	NA	absence	2	point count
835	Japan	Asia	NA	absence	3	point count
836	Japan	Asia	NA	absence	4	point count
837	Japan	Asia	NA	absence	6	point count
838	Japan	Asia	NA	absence	4	point count
839	Japan	Asia	NA	absence	5	point count
840	Japan	Asia	NA	absence	7	point count
841	Japan	Asia	NA	absence	4	point count
842	Japan	Asia	NA	absence	3	point count
843	Japan	Asia	NA	absence	5	point count
844	Japan	Asia	NA	absence	3	point count
845	Japan	Asia	NA	absence	4	point count
846	Japan	Asia	NA	absence	3	point count
847	Japan	Asia	NA	absence	4	point count
848	Japan	Asia	NA	absence	4	point count
849	Japan	Asia	NA	absence	4	point count

850	Japan	Asia	NA	absence	6	point count
851	Japan	Asia	NA	absence	5	point count
852	Japan	Asia	NA	absence	3	point count
853	Japan	Asia	NA	absence	5	point count
854	Japan	Asia	NA	absence	0	point count
855	Japan	Asia	NA	absence	2	point count
856	Japan	Asia	NA	absence	2	point count
857	Japan	Asia	NA	absence	2	point count
858	Japan	Asia	NA	absence	1	point count
859	Japan	Asia	NA	absence	4	point count
860	Japan	Asia	NA	absence	5	point count
861	Japan	Asia	NA	absence	0	point count
862	Japan	Asia	NA	absence	3	point count
863	Japan	Asia	NA	absence	4	point count
864	Japan	Asia	NA	absence	3	point count
865	Japan	Asia	NA	absence	3	point count
866	Japan	Asia	NA	absence	3	point count
867	Japan	Asia	NA	absence	6	point count
868	Japan	Asia	NA	absence	2	point count
869	Japan	Asia	NA	absence	4	point count
870	Japan	Asia	NA	absence	4	point count
871	Japan	Asia	NA	absence	4	point count
872	Japan	Asia	NA	absence	2	point count
873	Japan	Asia	NA	absence	2	point count
874	Japan	Asia	NA	absence	5	point count
875	Japan	Asia	NA	absence	3	point count
876	Japan	Asia	NA	absence	2	point count
877	Japan	Asia	NA	absence	3	point count
878	Japan	Asia	NA	absence	3	point count
879	Japan	Asia	NA	absence	4	point count
880	Japan	Asia	NA	absence	5	point count
881	Japan	Asia	NA	absence	3	point count
882	Japan	Asia	NA	absence	2	point count
883	Japan	Asia	NA	absence	4	point count
884	Japan	Asia	NA	absence	1	point count
885	Japan	Asia	NA	absence	2	point count
886	Japan	Asia	NA	absence	3	point count
887	Japan	Asia	NA	absence	3	point count
888	Japan	Asia	NA	absence	3	point count
889	Japan	Asia	NA	absence	3	point count
890	Japan	Asia	NA	absence	1	point count
891	Japan	Asia	NA	absence	5	point count
892	Japan	Asia	NA	absence	4	point count
893	Japan	Asia	NA	absence	2	point count
894	Japan	Asia	NA	absence	2	point count
895	Japan	Asia	NA	absence	6	point count
896	Japan	Asia	NA	absence	4	point count
897	Japan	Asia	NA	absence	4	point count
898	Japan	Asia	NA	absence	4	point count
899	Japan	Asia	NA	absence	3	point count

900	Japan	Asia	NA	absence	3	point count
901	Japan	Asia	NA	absence	4	point count
902	Japan	Asia	NA	absence	6	point count
903	Japan	Asia	NA	absence	4	point count
904	Japan	Asia	NA	absence	7	point count
905	Japan	Asia	NA	absence	5	point count
906	Japan	Asia	NA	absence	6	point count
907	Japan	Asia	NA	absence	6	point count
908	Japan	Asia	NA	absence	6	point count
909	Japan	Asia	NA	absence	6	point count
910	Japan	Asia	NA	absence	6	point count
911	Japan	Asia	NA	absence	5	point count
912	Japan	Asia	NA	absence	6	point count
913	Japan	Asia	NA	absence	5	point count
914	Japan	Asia	NA	absence	4	point count
915	Japan	Asia	NA	absence	4	point count
916	Japan	Asia	NA	absence	4	point count
917	Japan	Asia	NA	absence	4	point count
918	Japan	Asia	NA	absence	8	point count
919	Japan	Asia	NA	absence	8	point count
920	Japan	Asia	NA	absence	3	point count
921	Japan	Asia	NA	absence	6	point count
922	Japan	Asia	NA	absence	9	point count
923	Japan	Asia	NA	absence	7	point count
924	Japan	Asia	NA	absence	6	point count
925	Japan	Asia	NA	absence	7	point count
926	Japan	Asia	NA	absence	7	point count
927	Japan	Asia	NA	absence	8	point count
928	Japan	Asia	NA	absence	8	point count
929	Japan	Asia	NA	absence	6	point count
930	Japan	Asia	NA	absence	6	point count
931	Japan	Asia	NA	absence	5	point count
932	Japan	Asia	NA	absence	5	point count
933	Japan	Asia	NA	absence	6	point count
934	Japan	Asia	NA	absence	8	point count
935	Japan	Asia	NA	absence	6	point count
936	Japan	Asia	NA	absence	7	point count
937	Japan	Asia	NA	absence	6	point count
938	Japan	Asia	NA	absence	9	point count
939	Japan	Asia	NA	absence	5	point count
940	Japan	Asia	NA	absence	7	point count
941	Japan	Asia	NA	absence	3	point count
942	Japan	Asia	NA	absence	7	point count
943	Japan	Asia	NA	absence	6	point count
944	Japan	Asia	NA	absence	4	point count
945	Japan	Asia	NA	absence	5	point count
946	Japan	Asia	NA	absence	5	point count
947	Japan	Asia	NA	absence	5	point count
948	Japan	Asia	NA	absence	4	point count
949	Japan	Asia	NA	absence	6	point count

950	Japan	Asia	NA	absence	3	point count
951	Japan	Asia	NA	absence	5	point count
952	Japan	Asia	NA	absence	4	point count
953	Japan	Asia	NA	absence	5	point count
954	Japan	Asia	NA	absence	6	point count
955	Japan	Asia	NA	absence	4	point count
956	Japan	Asia	NA	absence	7	point count
957	Japan	Asia	NA	absence	6	point count
958	Japan	Asia	NA	absence	5	point count
959	Japan	Asia	NA	absence	4	point count
960	Japan	Asia	NA	absence	7	point count
961	Japan	Asia	NA	absence	5	point count
962	Japan	Asia	NA	absence	4	point count
963	Japan	Asia	NA	absence	5	point count
964	Japan	Asia	NA	absence	6	point count
965	Japan	Asia	NA	absence	5	point count
966	Japan	Asia	NA	absence	5	point count
967	Japan	Asia	NA	absence	5	point count
968	Japan	Asia	NA	absence	3	point count
969	Japan	Asia	NA	absence	5	point count
970	Japan	Asia	NA	absence	4	point count
971	Japan	Asia	NA	absence	4	point count
972	Japan	Asia	NA	absence	3	point count
973	Japan	Asia	NA	absence	4	point count
974	Japan	Asia	NA	absence	3	point count
975	Japan	Asia	NA	absence	6	point count
976	Japan	Asia	NA	absence	9	point count
977	Japan	Asia	NA	absence	6	point count
978	Japan	Asia	NA	absence	2	point count
979	Japan	Asia	NA	absence	6	point count
980	Japan	Asia	NA	absence	4	point count
981	Japan	Asia	NA	absence	7	point count
982	Japan	Asia	NA	absence	6	point count
983	Japan	Asia	NA	absence	6	point count
984	Japan	Asia	NA	absence	6	point count
985	Japan	Asia	NA	absence	4	point count
986	Japan	Asia	NA	absence	4	point count
987	Japan	Asia	NA	absence	6	point count
988	Japan	Asia	NA	absence	5	point count
989	Japan	Asia	NA	absence	6	point count
990	Japan	Asia	NA	absence	5	point count
991	Japan	Asia	NA	absence	6	point count
992	Japan	Asia	NA	absence	5	point count
993	Japan	Asia	NA	absence	4	point count
994	Japan	Asia	NA	absence	5	point count
995	Japan	Asia	NA	absence	6	point count
996	Japan	Asia	NA	absence	5	point count
997	Japan	Asia	NA	absence	6	point count
998	Japan	Asia	NA	absence	7	point count
999	Japan	Asia	NA	absence	6	point count

1000	Japan	Asia	NA		absence	7	point count
1001	Japan	Asia	NA		absence	6	point count
1002	Japan	Asia	NA		absence	6	point count
1003	Japan	Asia	NA		absence	7	point count
1004	Japan	Asia	NA		absence	5	point count
1005	Japan	Asia	NA		absence	7	point count
1006	Japan	Asia	NA		absence	6	point count
1007	Japan	Asia	NA		absence	6	point count
1008	Japan	Asia	NA		absence	10	point count
1009	Japan	Asia	NA		absence	6	point count
1010	Japan	Asia	NA		absence	5	point count
1011	Japan	Asia	NA		absence	3	point count
1012	Japan	Asia	NA		absence	4	point count
1013	Japan	Asia	NA		absence	3	point count
1014	Japan	Asia	NA		absence	3	point count
1015	Japan	Asia	NA		absence	4	point count
1016	Japan	Asia	NA		absence	5	point count
1017	Japan	Asia	NA		absence	4	point count
1018	Japan	Asia	NA		absence	4	point count
1019	Japan	Asia	NA		absence	4	point count
1020	Japan	Asia	NA		absence	3	point count
1021	Japan	Asia	NA		absence	5	point count
1022	Japan	Asia	NA		absence	4	point count
1023	Japan	Asia	NA		absence	4	point count
1024	Japan	Asia	NA		absence	5	point count
1025	Japan	Asia	NA		absence	5	point count
1026	Japan	Asia	NA		absence	3	point count
1027	Japan	Asia	NA		absence	7	point count
1028	Japan	Asia	NA		absence	3	point count
1029	Japan	Asia	NA		absence	5	point count
1030	Japan	Asia	NA		absence	3	point count
1031	Japan	Asia	NA		absence	5	point count
1032	Japan	Asia	NA		absence	2	point count
1033	Japan	Asia	NA		absence	4	point count
1034	Japan	Asia	NA		absence	5	point count
1035	Japan	Asia	NA		absence	3	point count
1036	Japan	Asia	NA		absence	1	point count
1037	Japan	Asia	NA		absence	2	point count
1038	Japan	Asia	NA		absence	3	point count
1039	Japan	Asia	NA		absence	2	point count
1040	Japan	Asia	NA		absence	3	point count
1041	Japan	Asia	NA		absence	2	point count
1042	Japan	Asia	NA		absence	4	point count
1043	Japan	Asia	NA		absence	4	point count
1044	Japan	Asia	NA		absence	4	point count
1045	Japan	Asia	NA		absence	3	point count
1046	Japan	Asia	NA		absence	5	point count
1047	Japan	Asia	NA		absence	2	point count
1048	Japan	Asia	NA		absence	2	point count
1049	Japan	Asia	NA		absence	3	point count

1050	Japan	Asia	NA	absence	5	point count
1051	Japan	Asia	NA	absence	1	point count
1052	Japan	Asia	NA	absence	3	point count
1053	Japan	Asia	NA	absence	4	point count
1054	Japan	Asia	NA	absence	6	point count
1055	Japan	Asia	NA	absence	5	point count
1056	Japan	Asia	NA	absence	4	point count
1057	Japan	Asia	NA	absence	2	point count
1058	Japan	Asia	NA	absence	3	point count
1059	Japan	Asia	NA	absence	2	point count
1060	Japan	Asia	NA	absence	2	point count
1061	Japan	Asia	NA	absence	3	point count
1062	Japan	Asia	NA	absence	4	point count
1063	Japan	Asia	NA	absence	5	point count
1064	Japan	Asia	NA	absence	6	point count
1065	Japan	Asia	NA	absence	4	point count
1066	Japan	Asia	NA	absence	5	point count
1067	Japan	Asia	NA	absence	4	point count
1068	Japan	Asia	NA	absence	6	point count
1069	Japan	Asia	NA	absence	3	point count
1070	Japan	Asia	NA	absence	5	point count
1071	Japan	Asia	NA	absence	5	point count
1072	Japan	Asia	NA	absence	5	point count
1073	Japan	Asia	NA	absence	5	point count
1074	Japan	Asia	NA	absence	3	point count
1075	Japan	Asia	NA	absence	2	point count
1076	Japan	Asia	NA	absence	3	point count
1077	Japan	Asia	NA	absence	3	point count
1078	Japan	Asia	NA	absence	2	point count
1079	Japan	Asia	NA	absence	3	point count
1080	Japan	Asia	NA	absence	3	point count
1081	Japan	Asia	NA	absence	1	point count
1082	Japan	Asia	NA	absence	3	point count
1083	Japan	Asia	NA	absence	1	point count
1084	Japan	Asia	NA	absence	1	point count
1085	Japan	Asia	NA	absence	2	point count
1086	Japan	Asia	NA	absence	2	point count
1087	Japan	Asia	NA	absence	1	point count
1088	Japan	Asia	NA	absence	3	point count
1089	Japan	Asia	NA	absence	1	point count
1090	Japan	Asia	NA	absence	1	point count
1091	Japan	Asia	NA	absence	1	point count
1092	Japan	Asia	NA	absence	3	point count
1093	Japan	Asia	NA	absence	1	point count
1094	Japan	Asia	NA	absence	1	point count
1095	Japan	Asia	NA	absence	0	point count
1096	Japan	Asia	NA	absence	2	point count
1097	Japan	Asia	NA	absence	1	point count
1098	Japan	Asia	NA	absence	2	point count
1099	Japan	Asia	NA	absence	2	point count

1100	Japan	Asia	NA	absence	1	point count
1101	Japan	Asia	NA	absence	2	point count
1102	Japan	Asia	NA	absence	3	point count
1103	Japan	Asia	NA	absence	3	point count
1104	Japan	Asia	NA	absence	2	point count
1105	Japan	Asia	NA	absence	2	point count
1106	Japan	Asia	NA	absence	2	point count
1107	Japan	Asia	NA	absence	2	point count
1108	Japan	Asia	NA	absence	2	point count
1109	Japan	Asia	NA	absence	2	point count
1110	Japan	Asia	NA	absence	1	point count
1111	Japan	Asia	NA	absence	3	point count
1112	Japan	Asia	NA	absence	2	point count
1113	Japan	Asia	NA	absence	3	point count
1114	Japan	Asia	NA	absence	3	point count
1115	Japan	Asia	NA	absence	3	point count
1116	Japan	Asia	NA	absence	2	point count
1117	Japan	Asia	NA	absence	1	point count
1118	Japan	Asia	NA	absence	1	point count
1119	Japan	Asia	NA	absence	1	point count
1120	Japan	Asia	NA	absence	3	point count
1121	Japan	Asia	NA	absence	5	point count
1122	Poland	Europe	farmland	presence	7	point count
1123	Poland	Europe	farmland	absence	3	point count
1124	Poland	Europe	farmland	presence	8	point count
1125	Poland	Europe	farmland	absence	4	point count
1126	Poland	Europe	farmland	presence	4	point count
1127	Poland	Europe	farmland	absence	4	point count
1128	Poland	Europe	farmland	presence	8	point count
1129	Poland	Europe	farmland	absence	4	point count
1130	Poland	Europe	farmland	presence	6	point count
1131	Poland	Europe	farmland	absence	6	point count
1132	Poland	Europe	farmland	presence	12	point count
1133	Poland	Europe	farmland	absence	7	point count
1134	Poland	Europe	farmland	presence	8	point count
1135	Poland	Europe	farmland	absence	6	point count
1136	Poland	Europe	farmland	presence	12	point count
1137	Poland	Europe	farmland	absence	5	point count
1138	Poland	Europe	farmland	presence	7	point count
1139	Poland	Europe	farmland	absence	6	point count
1140	Poland	Europe	farmland	presence	10	point count
1141	Poland	Europe	farmland	absence	5	point count
1142	Poland	Europe	farmland	presence	9	point count
1143	Poland	Europe	farmland	absence	5	point count
1144	Poland	Europe	farmland	presence	7	point count
1145	Poland	Europe	farmland	absence	4	point count
1146	Poland	Europe	farmland	presence	9	point count
1147	Poland	Europe	farmland	absence	6	point count
1148	Poland	Europe	farmland	presence	8	point count
1149	Poland	Europe	farmland	absence	5	point count

1150	Poland	Europe	farmland	presence	7	point count
1151	Poland	Europe	farmland	absence	4	point count
1152	Poland	Europe	farmland	presence	10	point count
1153	Poland	Europe	farmland	absence	5	point count
1154	Poland	Europe	farmland	presence	6	point count
1155	Poland	Europe	farmland	absence	4	point count
1156	Poland	Europe	farmland	presence	8	point count
1157	Poland	Europe	farmland	absence	6	point count
1158	Poland	Europe	farmland	presence	10	point count
1159	Poland	Europe	farmland	absence	8	point count
1160	Poland	Europe	farmland	presence	10	point count
1161	Poland	Europe	farmland	absence	6	point count
1162	Poland	Europe	farmland	presence	12	point count
1163	Poland	Europe	farmland	absence	9	point count
1164	Poland	Europe	farmland	presence	14	point count
1165	Poland	Europe	farmland	absence	9	point count
1166	Poland	Europe	farmland	presence	8	point count
1167	Poland	Europe	farmland	absence	4	point count
1168	Poland	Europe	farmland	presence	6	point count
1169	Poland	Europe	farmland	absence	4	point count
1170	Poland	Europe	farmland	presence	5	point count
1171	Poland	Europe	farmland	absence	4	point count
1172	Poland	Europe	farmland	presence	7	point count
1173	Poland	Europe	farmland	absence	5	point count
1174	Poland	Europe	farmland	presence	6	point count
1175	Poland	Europe	farmland	absence	5	point count
1176	Poland	Europe	farmland	presence	11	point count
1177	Poland	Europe	farmland	absence	6	point count
1178	Poland	Europe	farmland	presence	3	point count
1179	Poland	Europe	farmland	absence	3	point count
1180	Poland	Europe	farmland	presence	6	point count
1181	Poland	Europe	farmland	absence	2	point count
1182	Poland	Europe	farmland	presence	6	point count
1183	Poland	Europe	farmland	absence	5	point count
1184	Poland	Europe	farmland	presence	6	point count
1185	Poland	Europe	farmland	absence	6	point count
1186	Poland	Europe	farmland	presence	4	point count
1187	Poland	Europe	farmland	absence	4	point count
1188	Poland	Europe	farmland	presence	9	point count
1189	Poland	Europe	farmland	absence	5	point count
1190	Poland	Europe	farmland	presence	9	point count
1191	Poland	Europe	farmland	absence	7	point count
1192	Poland	Europe	farmland	presence	14	point count
1193	Poland	Europe	farmland	absence	9	point count
1194	Poland	Europe	farmland	presence	10	point count
1195	Poland	Europe	farmland	absence	7	point count
1196	Poland	Europe	farmland	presence	12	point count
1197	Poland	Europe	farmland	absence	5	point count
1198	Poland	Europe	farmland	presence	9	point count
1199	Poland	Europe	farmland	absence	7	point count

1200	Poland	Europe	farmland	presence	8	point count
1201	Poland	Europe	farmland	absence	4	point count
1202	Poland	Europe	farmland	presence	9	point count
1203	Poland	Europe	farmland	absence	5	point count
1204	Poland	Europe	farmland	presence	12	point count
1205	Poland	Europe	farmland	absence	6	point count
1206	Poland	Europe	farmland	presence	8	point count
1207	Poland	Europe	farmland	absence	5	point count
1208	Poland	Europe	farmland	presence	6	point count
1209	Poland	Europe	farmland	absence	5	point count
1210	Poland	Europe	farmland	presence	6	point count
1211	Poland	Europe	farmland	absence	4	point count
1212	Poland	Europe	farmland	presence	6	point count
1213	Poland	Europe	farmland	absence	4	point count
1214	Poland	Europe	farmland	presence	8	point count
1215	Poland	Europe	farmland	absence	6	point count
1216	Poland	Europe	farmland	presence	8	point count
1217	Poland	Europe	farmland	absence	5	point count
1218	Poland	Europe	farmland	presence	7	point count
1219	Poland	Europe	farmland	absence	5	point count
1220	Poland	Europe	farmland	presence	10	point count
1221	Poland	Europe	farmland	absence	8	point count
1222	Poland	Europe	farmland	presence	9	point count
1223	Poland	Europe	farmland	absence	5	point count
1224	Poland	Europe	farmland	presence	6	point count
1225	Poland	Europe	farmland	absence	5	point count
1226	Poland	Europe	farmland	presence	9	point count
1227	Poland	Europe	farmland	absence	6	point count
1228	Poland	Europe	farmland	presence	5	point count
1229	Poland	Europe	farmland	absence	5	point count
1230	Poland	Europe	farmland	presence	6	point count
1231	Poland	Europe	farmland	absence	4	point count
1232	Poland	Europe	farmland	presence	9	point count
1233	Poland	Europe	farmland	absence	6	point count
1234	Poland	Europe	farmland	presence	9	point count
1235	Poland	Europe	farmland	absence	8	point count
1236	Poland	Europe	farmland	presence	9	point count
1237	Poland	Europe	farmland	absence	6	point count
1238	Poland	Europe	farmland	presence	9	point count
1239	Poland	Europe	farmland	absence	7	point count
1240	Poland	Europe	farmland	presence	9	point count
1241	Poland	Europe	farmland	absence	5	point count
1242	Poland	Europe	farmland	presence	10	point count
1243	Poland	Europe	farmland	absence	6	point count
1244	Poland	Europe	farmland	presence	8	point count
1245	Poland	Europe	farmland	absence	7	point count
1246	Poland	Europe	farmland	presence	11	point count
1247	Poland	Europe	farmland	absence	8	point count
1248	Poland	Europe	farmland	presence	10	point count
1249	Poland	Europe	farmland	absence	7	point count

1250	Poland	Europe	farmland	presence	10	point count
1251	Poland	Europe	farmland	absence	8	point count
1252	Poland	Europe	farmland	presence	7	point count
1253	Poland	Europe	farmland	absence	6	point count
1254	Poland	Europe	farmland	presence	7	point count
1255	Poland	Europe	farmland	absence	4	point count
1256	Poland	Europe	farmland	presence	9	point count
1257	Poland	Europe	farmland	absence	7	point count
1258	Poland	Europe	farmland	presence	15	point count
1259	Poland	Europe	farmland	absence	10	point count
1260	Poland	Europe	farmland	presence	9	point count
1261	Poland	Europe	farmland	absence	5	point count
1262	Poland	Europe	farmland	presence	13	point count
1263	Poland	Europe	farmland	absence	12	point count
1264	Poland	Europe	farmland	presence	12	point count
1265	Poland	Europe	farmland	absence	12	point count
1266	Poland	Europe	farmland	presence	9	point count
1267	Poland	Europe	farmland	absence	8	point count
1268	Poland	Europe	farmland	presence	11	point count
1269	Poland	Europe	farmland	absence	5	point count
1270	Poland	Europe	farmland	presence	10	point count
1271	Poland	Europe	farmland	absence	7	point count
1272	Poland	Europe	farmland	presence	15	point count
1273	Poland	Europe	farmland	absence	7	point count
1274	Poland	Europe	farmland	presence	9	point count
1275	Poland	Europe	farmland	absence	6	point count
1276	Poland	Europe	farmland	presence	8	point count
1277	Poland	Europe	farmland	absence	7	point count
1278	Poland	Europe	farmland	presence	11	point count
1279	Poland	Europe	farmland	absence	6	point count
1280	Poland	Europe	farmland	presence	8	point count
1281	Poland	Europe	farmland	absence	8	point count
1282	Poland	Europe	farmland	presence	13	point count
1283	Poland	Europe	farmland	absence	9	point count
1284	Poland	Europe	farmland	presence	9	point count
1285	Poland	Europe	farmland	absence	8	point count
1286	Poland	Europe	farmland	presence	13	point count
1287	Poland	Europe	farmland	absence	9	point count
1288	Poland	Europe	farmland	presence	7	point count
1289	Poland	Europe	farmland	absence	3	point count
1290	Poland	Europe	farmland	presence	8	point count
1291	Poland	Europe	farmland	absence	4	point count
1292	Poland	Europe	farmland	presence	4	point count
1293	Poland	Europe	farmland	absence	4	point count
1294	Poland	Europe	farmland	presence	8	point count
1295	Poland	Europe	farmland	absence	4	point count
1296	Poland	Europe	farmland	presence	6	point count
1297	Poland	Europe	farmland	absence	6	point count
1298	Poland	Europe	farmland	presence	12	point count
1299	Poland	Europe	farmland	absence	7	point count

1300	Poland	Europe	farmland	presence	8	point count
1301	Poland	Europe	farmland	absence	6	point count
1302	Poland	Europe	farmland	presence	12	point count
1303	Poland	Europe	farmland	absence	5	point count
1304	Poland	Europe	farmland	presence	7	point count
1305	Poland	Europe	farmland	absence	6	point count
1306	Poland	Europe	farmland	presence	10	point count
1307	Poland	Europe	farmland	absence	5	point count
1308	Poland	Europe	farmland	presence	9	point count
1309	Poland	Europe	farmland	absence	5	point count
1310	Poland	Europe	farmland	presence	7	point count
1311	Poland	Europe	farmland	absence	4	point count
1312	Poland	Europe	farmland	presence	9	point count
1313	Poland	Europe	farmland	absence	6	point count
1314	Poland	Europe	farmland	presence	8	point count
1315	Poland	Europe	farmland	absence	5	point count
1316	Poland	Europe	farmland	presence	7	point count
1317	Poland	Europe	farmland	absence	4	point count
1318	Poland	Europe	farmland	presence	10	point count
1319	Poland	Europe	farmland	absence	5	point count
1320	Poland	Europe	farmland	presence	6	point count
1321	Poland	Europe	farmland	absence	4	point count
1322	Poland	Europe	farmland	presence	8	point count
1323	Poland	Europe	farmland	absence	6	point count
1324	Poland	Europe	farmland	presence	10	point count
1325	Poland	Europe	farmland	absence	8	point count
1326	Poland	Europe	farmland	presence	10	point count
1327	Poland	Europe	farmland	absence	6	point count
1328	Poland	Europe	farmland	presence	12	point count
1329	Poland	Europe	farmland	absence	9	point count
1330	Poland	Europe	farmland	presence	14	point count
1331	Poland	Europe	farmland	absence	9	point count
1332	Poland	Europe	farmland	presence	8	point count
1333	Poland	Europe	farmland	absence	4	point count
1334	Poland	Europe	farmland	presence	6	point count
1335	Poland	Europe	farmland	absence	4	point count
1336	Poland	Europe	farmland	presence	5	point count
1337	Poland	Europe	farmland	absence	4	point count
1338	Poland	Europe	farmland	presence	7	point count
1339	Poland	Europe	farmland	absence	5	point count
1340	Poland	Europe	farmland	presence	6	point count
1341	Poland	Europe	farmland	absence	5	point count
1342	Poland	Europe	farmland	presence	11	point count
1343	Poland	Europe	farmland	absence	6	point count
1344	Poland	Europe	farmland	presence	3	point count
1345	Poland	Europe	farmland	absence	3	point count
1346	Poland	Europe	farmland	presence	6	point count
1347	Poland	Europe	farmland	absence	2	point count
1348	Poland	Europe	farmland	presence	6	point count
1349	Poland	Europe	farmland	absence	5	point count

1350	Poland	Europe	farmland	presence	6	point count
1351	Poland	Europe	farmland	absence	6	point count
1352	Poland	Europe	farmland	presence	4	point count
1353	Poland	Europe	farmland	absence	4	point count
1354	Poland	Europe	farmland	presence	9	point count
1355	Poland	Europe	farmland	absence	5	point count
1356	Poland	Europe	farmland	presence	9	point count
1357	Poland	Europe	farmland	absence	7	point count
1358	Poland	Europe	farmland	presence	14	point count
1359	Poland	Europe	farmland	absence	9	point count
1360	Poland	Europe	farmland	presence	10	point count
1361	Poland	Europe	farmland	absence	7	point count
1362	Poland	Europe	farmland	presence	12	point count
1363	Poland	Europe	farmland	absence	5	point count
1364	Poland	Europe	farmland	presence	9	point count
1365	Poland	Europe	farmland	absence	7	point count
1366	Poland	Europe	farmland	presence	8	point count
1367	Poland	Europe	farmland	absence	4	point count
1368	Poland	Europe	farmland	presence	9	point count
1369	Poland	Europe	farmland	absence	5	point count
1370	Poland	Europe	farmland	presence	12	point count
1371	Poland	Europe	farmland	absence	6	point count
1372	Poland	Europe	farmland	presence	8	point count
1373	Poland	Europe	farmland	absence	5	point count
1374	Poland	Europe	farmland	presence	6	point count
1375	Poland	Europe	farmland	absence	5	point count
1376	Poland	Europe	farmland	presence	6	point count
1377	Poland	Europe	farmland	absence	4	point count
1378	Poland	Europe	farmland	presence	6	point count
1379	Poland	Europe	farmland	absence	4	point count
1380	Poland	Europe	farmland	presence	8	point count
1381	Poland	Europe	farmland	absence	6	point count
1382	Poland	Europe	farmland	presence	8	point count
1383	Poland	Europe	farmland	absence	5	point count
1384	Poland	Europe	farmland	presence	7	point count
1385	Poland	Europe	farmland	absence	5	point count
1386	Poland	Europe	farmland	presence	10	point count
1387	Poland	Europe	farmland	absence	8	point count
1388	Poland	Europe	farmland	presence	9	point count
1389	Poland	Europe	farmland	absence	5	point count
1390	Poland	Europe	farmland	presence	6	point count
1391	Poland	Europe	farmland	absence	5	point count
1392	Poland	Europe	farmland	presence	9	point count
1393	Poland	Europe	farmland	absence	6	point count
1394	Poland	Europe	farmland	presence	5	point count
1395	Poland	Europe	farmland	absence	5	point count
1396	Poland	Europe	farmland	presence	6	point count
1397	Poland	Europe	farmland	absence	4	point count
1398	Poland	Europe	farmland	presence	9	point count
1399	Poland	Europe	farmland	absence	6	point count

1400	Poland	Europe	farmland	presence	9	point count
1401	Poland	Europe	farmland	absence	8	point count
1402	Poland	Europe	farmland	presence	9	point count
1403	Poland	Europe	farmland	absence	6	point count
1404	Poland	Europe	farmland	presence	9	point count
1405	Poland	Europe	farmland	absence	7	point count
1406	Poland	Europe	farmland	presence	9	point count
1407	Poland	Europe	farmland	absence	5	point count
1408	Poland	Europe	farmland	presence	10	point count
1409	Poland	Europe	farmland	absence	6	point count
1410	Poland	Europe	farmland	presence	8	point count
1411	Poland	Europe	farmland	absence	7	point count
1412	Poland	Europe	farmland	presence	11	point count
1413	Poland	Europe	farmland	absence	8	point count
1414	Poland	Europe	farmland	presence	10	point count
1415	Poland	Europe	farmland	absence	7	point count
1416	Poland	Europe	farmland	presence	10	point count
1417	Poland	Europe	farmland	absence	8	point count
1418	Poland	Europe	farmland	presence	7	point count
1419	Poland	Europe	farmland	absence	6	point count
1420	Poland	Europe	farmland	presence	7	point count
1421	Poland	Europe	farmland	absence	4	point count
1422	Poland	Europe	farmland	presence	9	point count
1423	Poland	Europe	farmland	absence	7	point count
1424	Poland	Europe	farmland	presence	15	point count
1425	Poland	Europe	farmland	absence	10	point count
1426	Poland	Europe	farmland	presence	9	point count
1427	Poland	Europe	farmland	absence	5	point count
1428	Poland	Europe	farmland	presence	13	point count
1429	Poland	Europe	farmland	absence	12	point count
1430	Poland	Europe	farmland	presence	12	point count
1431	Poland	Europe	farmland	absence	12	point count
1432	Poland	Europe	farmland	presence	9	point count
1433	Poland	Europe	farmland	absence	8	point count
1434	Poland	Europe	farmland	presence	11	point count
1435	Poland	Europe	farmland	absence	5	point count
1436	Poland	Europe	farmland	presence	10	point count
1437	Poland	Europe	farmland	absence	7	point count
1438	Poland	Europe	farmland	presence	15	point count
1439	Poland	Europe	farmland	absence	7	point count
1440	Poland	Europe	farmland	presence	9	point count
1441	Poland	Europe	farmland	absence	6	point count
1442	Poland	Europe	farmland	presence	8	point count
1443	Poland	Europe	farmland	absence	7	point count
1444	Poland	Europe	farmland	presence	11	point count
1445	Poland	Europe	farmland	absence	6	point count
1446	Poland	Europe	farmland	presence	8	point count
1447	Poland	Europe	farmland	absence	8	point count
1448	Poland	Europe	farmland	presence	13	point count
1449	Poland	Europe	farmland	absence	9	point count

1450	Poland	Europe	farmland	presence	9	point count
1451	Poland	Europe	farmland	absence	8	point count
1452	Poland	Europe	farmland	presence	13	point count
1453	Poland	Europe	farmland	absence	9	point count
1454	San Marino	Europe	mixed	absence	1	point count
1455	San Marino	Europe	mixed	absence	12	point count
1456	San Marino	Europe	mixed	absence	6	point count
1457	San Marino	Europe	mixed	presence	24	point count
1458	San Marino	Europe	mixed	absence	1	point count
1459	San Marino	Europe	mixed	absence	19	point count
1460	San Marino	Europe	mixed	presence	16	point count
1461	San Marino	Europe	mixed	absence	4	point count
1462	San Marino	Europe	mixed	absence	2	point count
1463	San Marino	Europe	mixed	absence	22	point count
1464	San Marino	Europe	mixed	absence	3	point count
1465	San Marino	Europe	mixed	absence	1	point count
1466	San Marino	Europe	mixed	presence	7	point count
1467	San Marino	Europe	mixed	absence	1	point count
1468	San Marino	Europe	mixed	absence	2	point count
1469	San Marino	Europe	mixed	absence	8	point count
1470	San Marino	Europe	mixed	absence	1	point count
1471	San Marino	Europe	mixed	absence	6	point count
1472	San Marino	Europe	mixed	absence	3	point count
1473	San Marino	Europe	mixed	absence	1	point count
1474	San Marino	Europe	mixed	presence	20	point count
1475	San Marino	Europe	mixed	presence	16	point count
1476	San Marino	Europe	mixed	presence	14	point count
1477	San Marino	Europe	mixed	presence	17	point count
1478	San Marino	Europe	mixed	absence	1	point count
1479	San Marino	Europe	mixed	absence	19	point count
1480	San Marino	Europe	mixed	presence	7	point count
1481	San Marino	Europe	mixed	absence	8	point count
1482	San Marino	Europe	mixed	absence	12	point count
1483	San Marino	Europe	mixed	absence	1	point count
1484	San Marino	Europe	mixed	presence	23	point count
1485	San Marino	Europe	mixed	absence	4	point count
1486	San Marino	Europe	mixed	absence	9	point count
1487	San Marino	Europe	mixed	absence	1	point count
1488	San Marino	Europe	mixed	absence	1	point count
1489	San Marino	Europe	mixed	absence	4	point count
1490	San Marino	Europe	mixed	absence	3	point count
1491	San Marino	Europe	mixed	absence	3	point count
1492	San Marino	Europe	mixed	absence	1	point count
1493	San Marino	Europe	mixed	absence	1	point count
1494	San Marino	Europe	mixed	presence	23	point count
1495	San Marino	Europe	mixed	absence	1	point count
1496	San Marino	Europe	mixed	presence	16	point count
1497	San Marino	Europe	mixed	absence	8	point count
1498	San Marino	Europe	mixed	absence	18	point count
1499	San Marino	Europe	mixed	presence	5	point count

1500	San Marino	Europe	mixed	absence	2	point count
1501	San Marino	Europe	mixed	absence	8	point count
1502	San Marino	Europe	mixed	absence	1	point count
1503	San Marino	Europe	mixed	absence	1	point count
1504	San Marino	Europe	mixed	presence	5	point count
1505	San Marino	Europe	mixed	absence	1	point count
1506	San Marino	Europe	mixed	presence	5	point count
1507	San Marino	Europe	mixed	absence	1	point count
1508	San Marino	Europe	mixed	absence	1	point count
1509	San Marino	Europe	mixed	presence	5	point count
1510	San Marino	Europe	mixed	absence	3	point count
1511	San Marino	Europe	mixed	absence	2	point count
1512	San Marino	Europe	mixed	absence	2	point count
1513	San Marino	Europe	mixed	presence	7	point count
1514	San Marino	Europe	mixed	absence	3	point count
1515	San Marino	Europe	mixed	presence	18	point count
1516	San Marino	Europe	mixed	absence	11	point count
1517	San Marino	Europe	mixed	absence	9	point count
1518	San Marino	Europe	mixed	absence	22	point count
1519	San Marino	Europe	mixed	absence	1	point count
1520	San Marino	Europe	mixed	absence	2	point count
1521	San Marino	Europe	mixed	presence	15	point count
1522	San Marino	Europe	mixed	presence	20	point count
1523	San Marino	Europe	mixed	absence	8	point count
1524	San Marino	Europe	mixed	absence	15	point count
1525	San Marino	Europe	mixed	presence	5	point count
1526	San Marino	Europe	mixed	absence	3	point count
1527	San Marino	Europe	mixed	absence	18	point count
1528	San Marino	Europe	mixed	presence	16	point count
1529	San Marino	Europe	mixed	presence	5	point count
1530	San Marino	Europe	mixed	presence	16	point count
1531	San Marino	Europe	mixed	absence	1	point count
1532	San Marino	Europe	mixed	absence	1	point count
1533	San Marino	Europe	mixed	absence	1	point count
1534	San Marino	Europe	mixed	absence	2	point count
1535	San Marino	Europe	mixed	absence	1	point count
1536	San Marino	Europe	mixed	presence	10	point count
1537	San Marino	Europe	mixed	absence	1	point count
1538	San Marino	Europe	mixed	absence	3	point count
1539	San Marino	Europe	mixed	presence	23	point count
1540	San Marino	Europe	mixed	absence	1	point count
1541	San Marino	Europe	mixed	presence	5	point count
1542	San Marino	Europe	mixed	absence	4	point count
1543	San Marino	Europe	mixed	absence	2	point count
1544	San Marino	Europe	mixed	absence	1	point count
1545	San Marino	Europe	mixed	absence	9	point count
1546	San Marino	Europe	mixed	absence	6	point count
1547	San Marino	Europe	mixed	absence	1	point count
1548	San Marino	Europe	mixed	absence	6	point count
1549	San Marino	Europe	mixed	absence	4	point count

1550	San Marino	Europe	mixed	absence	4	point count
1551	San Marino	Europe	mixed	absence	1	point count
1552	San Marino	Europe	mixed	presence	21	point count
1553	San Marino	Europe	mixed	absence	3	point count
1554	San Marino	Europe	mixed	absence	4	point count
1555	San Marino	Europe	mixed	absence	1	point count
1556	San Marino	Europe	mixed	presence	23	point count
1557	San Marino	Europe	mixed	presence	19	point count
1558	San Marino	Europe	mixed	absence	4	point count
1559	San Marino	Europe	mixed	absence	4	point count
1560	San Marino	Europe	mixed	absence	18	point count
1561	San Marino	Europe	mixed	absence	2	point count
1562	San Marino	Europe	mixed	absence	3	point count
1563	San Marino	Europe	mixed	absence	4	point count
1564	San Marino	Europe	mixed	absence	6	point count
1565	San Marino	Europe	mixed	absence	8	point count
1566	San Marino	Europe	mixed	absence	2	point count
1567	San Marino	Europe	mixed	presence	7	point count
1568	San Marino	Europe	mixed	absence	1	point count
1569	San Marino	Europe	mixed	presence	5	point count
1570	San Marino	Europe	mixed	presence	16	point count
1571	San Marino	Europe	mixed	absence	2	point count
1572	San Marino	Europe	mixed	absence	1	point count
1573	San Marino	Europe	mixed	absence	12	point count
1574	San Marino	Europe	mixed	absence	3	point count
1575	San Marino	Europe	mixed	absence	1	point count
1576	San Marino	Europe	mixed	absence	2	point count
1577	San Marino	Europe	mixed	absence	3	point count
1578	San Marino	Europe	mixed	absence	3	point count
1579	San Marino	Europe	mixed	absence	3	point count
1580	San Marino	Europe	mixed	absence	1	point count
1581	San Marino	Europe	mixed	presence	16	point count
1582	San Marino	Europe	mixed	absence	8	point count
1583	San Marino	Europe	mixed	absence	2	point count
1584	San Marino	Europe	mixed	absence	22	point count
1585	San Marino	Europe	mixed	absence	2	point count
1586	San Marino	Europe	mixed	presence	9	point count
1587	San Marino	Europe	mixed	absence	4	point count
1588	San Marino	Europe	mixed	absence	1	point count
1589	San Marino	Europe	mixed	absence	1	point count
1590	San Marino	Europe	mixed	absence	1	point count
1591	San Marino	Europe	mixed	absence	3	point count
1592	San Marino	Europe	mixed	absence	1	point count
1593	San Marino	Europe	mixed	presence	7	point count
1594	San Marino	Europe	mixed	absence	2	point count
1595	San Marino	Europe	mixed	absence	1	point count
1596	San Marino	Europe	mixed	absence	2	point count
1597	San Marino	Europe	mixed	absence	1	point count
1598	San Marino	Europe	mixed	absence	1	point count
1599	San Marino	Europe	mixed	absence	6	point count

1600	San Marino	Europe	mixed	absence	1	point count
1601	San Marino	Europe	mixed	absence	2	point count
1602	San Marino	Europe	mixed	absence	19	point count
1603	San Marino	Europe	mixed	absence	1	point count
1604	San Marino	Europe	mixed	absence	15	point count
1605	San Marino	Europe	mixed	absence	8	point count
1606	San Marino	Europe	mixed	presence	11	point count
1607	San Marino	Europe	mixed	absence	3	point count
1608	San Marino	Europe	mixed	presence	7	point count
1609	San Marino	Europe	mixed	absence	4	point count
1610	San Marino	Europe	mixed	absence	12	point count
1611	San Marino	Europe	mixed	presence	17	point count
1612	San Marino	Europe	mixed	absence	3	point count
1613	San Marino	Europe	mixed	absence	3	point count
1614	San Marino	Europe	mixed	absence	4	point count
1615	San Marino	Europe	mixed	absence	2	point count
1616	San Marino	Europe	mixed	absence	8	point count
1617	San Marino	Europe	mixed	absence	3	point count
1618	San Marino	Europe	mixed	presence	23	point count
1619	San Marino	Europe	mixed	presence	16	point count
1620	San Marino	Europe	mixed	absence	1	point count
1621	San Marino	Europe	mixed	presence	18	point count
1622	San Marino	Europe	mixed	absence	3	point count
1623	San Marino	Europe	mixed	presence	19	point count
1624	San Marino	Europe	mixed	absence	11	point count
1625	San Marino	Europe	mixed	presence	7	point count
1626	San Marino	Europe	mixed	absence	8	point count
1627	San Marino	Europe	mixed	absence	3	point count
1628	San Marino	Europe	mixed	absence	1	point count
1629	San Marino	Europe	mixed	absence	4	point count
1630	San Marino	Europe	mixed	absence	1	point count
1631	San Marino	Europe	mixed	absence	1	point count
1632	San Marino	Europe	mixed	absence	3	point count
1633	San Marino	Europe	mixed	absence	2	point count
1634	San Marino	Europe	mixed	presence	11	point count
1635	San Marino	Europe	mixed	absence	19	point count
1636	San Marino	Europe	mixed	absence	15	point count
1637	San Marino	Europe	mixed	absence	2	point count
1638	San Marino	Europe	mixed	absence	1	point count
1639	San Marino	Europe	mixed	presence	7	point count
1640	San Marino	Europe	mixed	absence	2	point count
1641	San Marino	Europe	mixed	absence	3	point count
1642	San Marino	Europe	mixed	absence	19	point count
1643	San Marino	Europe	mixed	absence	1	point count
1644	San Marino	Europe	mixed	absence	1	point count
1645	San Marino	Europe	mixed	absence	2	point count
1646	San Marino	Europe	mixed	absence	1	point count
1647	San Marino	Europe	mixed	absence	4	point count
1648	San Marino	Europe	mixed	presence	13	point count
1649	San Marino	Europe	mixed	absence	3	point count

1650	San Marino	Europe	mixed	absence	19	point count
1651	San Marino	Europe	mixed	absence	19	point count
1652	San Marino	Europe	mixed	presence	21	point count
1653	San Marino	Europe	mixed	absence	6	point count
1654	San Marino	Europe	mixed	presence	14	point count
1655	San Marino	Europe	mixed	absence	1	point count
1656	San Marino	Europe	mixed	absence	4	point count
1657	San Marino	Europe	mixed	absence	2	point count
1658	San Marino	Europe	mixed	absence	8	point count
1659	San Marino	Europe	mixed	absence	4	point count
1660	San Marino	Europe	mixed	absence	1	point count
1661	San Marino	Europe	mixed	presence	13	point count
1662	San Marino	Europe	mixed	presence	20	point count
1663	San Marino	Europe	mixed	absence	3	point count
1664	San Marino	Europe	mixed	absence	1	point count
1665	San Marino	Europe	mixed	absence	1	point count
1666	San Marino	Europe	mixed	presence	13	point count
1667	San Marino	Europe	mixed	absence	9	point count
1668	San Marino	Europe	mixed	absence	11	point count
1669	San Marino	Europe	mixed	absence	2	point count
1670	San Marino	Europe	mixed	absence	1	point count
1671	San Marino	Europe	mixed	absence	3	point count
1672	San Marino	Europe	mixed	absence	2	point count
1673	San Marino	Europe	mixed	absence	2	point count
1674	San Marino	Europe	mixed	absence	2	point count
1675	San Marino	Europe	mixed	absence	1	point count
1676	San Marino	Europe	mixed	absence	2	point count
1677	San Marino	Europe	mixed	presence	5	point count
1678	San Marino	Europe	mixed	absence	4	point count
1679	San Marino	Europe	mixed	absence	15	point count
1680	San Marino	Europe	mixed	absence	4	point count
1681	San Marino	Europe	mixed	absence	1	point count
1682	San Marino	Europe	mixed	absence	4	point count
1683	San Marino	Europe	mixed	absence	3	point count
1684	San Marino	Europe	mixed	absence	9	point count
1685	San Marino	Europe	mixed	presence	16	point count
1686	San Marino	Europe	mixed	absence	2	point count
1687	San Marino	Europe	mixed	absence	19	point count
1688	San Marino	Europe	mixed	absence	1	point count
1689	San Marino	Europe	mixed	absence	1	point count
1690	San Marino	Europe	mixed	absence	12	point count
1691	San Marino	Europe	mixed	absence	8	point count
1692	San Marino	Europe	mixed	absence	1	point count
1693	San Marino	Europe	mixed	presence	13	point count
1694	San Marino	Europe	mixed	absence	1	point count
1695	San Marino	Europe	mixed	absence	18	point count
1696	San Marino	Europe	mixed	absence	2	point count
1697	San Marino	Europe	mixed	absence	1	point count
1698	San Marino	Europe	mixed	presence	16	point count
1699	San Marino	Europe	mixed	presence	7	point count

1700	San Marino	Europe	mixed	presence	17	point count
1701	San Marino	Europe	mixed	absence	2	point count
1702	San Marino	Europe	mixed	absence	1	point count
1703	San Marino	Europe	mixed	absence	1	point count
1704	Switzerland	Europe	forest	absence	11	point count
1705	Switzerland	Europe	forest	absence	12	point count
1706	Switzerland	Europe	forest	absence	10	point count
1707	Switzerland	Europe	forest	absence	18	point count
1708	Switzerland	Europe	forest	absence	13	point count
1709	Switzerland	Europe	forest	absence	15	point count
1710	Switzerland	Europe	forest	presence	18	point count
1711	Switzerland	Europe	forest	absence	16	point count
1712	Switzerland	Europe	forest	absence	13	point count
1713	Switzerland	Europe	forest	absence	12	point count
1714	Switzerland	Europe	forest	absence	17	point count
1715	Switzerland	Europe	forest	absence	13	point count
1716	Switzerland	Europe	forest	presence	16	point count
1717	Switzerland	Europe	forest	absence	16	point count
1718	Switzerland	Europe	forest	absence	18	point count
1719	Switzerland	Europe	forest	presence	20	point count
1720	Switzerland	Europe	forest	absence	12	point count
1721	Switzerland	Europe	forest	absence	16	point count
1722	Switzerland	Europe	forest	absence	11	point count
1723	Switzerland	Europe	forest	absence	11	point count
1724	Switzerland	Europe	forest	absence	17	point count
1725	Switzerland	Europe	forest	absence	14	point count
1726	Switzerland	Europe	forest	absence	12	point count
1727	Switzerland	Europe	forest	absence	15	point count
1728	Switzerland	Europe	forest	absence	19	point count
1729	Switzerland	Europe	forest	absence	17	point count
1730	Switzerland	Europe	forest	presence	15	point count
1731	Switzerland	Europe	forest	presence	18	point count
1732	Switzerland	Europe	forest	absence	17	point count
1733	Switzerland	Europe	forest	presence	21	point count
1734	Switzerland	Europe	forest	presence	23	point count
1735	Switzerland	Europe	forest	presence	21	point count
1736	Switzerland	Europe	forest	absence	17	point count
1737	Switzerland	Europe	forest	absence	11	point count
1738	Switzerland	Europe	forest	presence	15	point count
1739	Switzerland	Europe	forest	absence	14	point count
1740	Switzerland	Europe	forest	absence	12	point count
1741	Switzerland	Europe	forest	absence	22	point count
1742	Switzerland	Europe	forest	absence	16	point count
1743	Switzerland	Europe	forest	absence	15	point count
1744	Switzerland	Europe	forest	absence	16	point count
1745	Switzerland	Europe	forest	absence	16	point count
1746	Switzerland	Europe	forest	absence	20	point count
1747	Switzerland	Europe	forest	absence	16	point count
1748	Switzerland	Europe	forest	absence	15	point count
1749	Switzerland	Europe	forest	absence	16	point count

1750	Switzerland	Europe	forest	absence	20	point count
1751	Switzerland	Europe	forest	presence	28	point count
1752	Switzerland	Europe	forest	absence	15	point count
1753	Switzerland	Europe	forest	absence	22	point count
1754	Switzerland	Europe	forest	absence	16	point count
1755	Switzerland	Europe	forest	absence	18	point count
1756	Switzerland	Europe	forest	absence	16	point count
1757	Switzerland	Europe	forest	absence	19	point count
1758	Switzerland	Europe	forest	absence	17	point count
1759	Switzerland	Europe	forest	presence	21	point count
1760	Switzerland	Europe	forest	presence	28	point count
1761	Switzerland	Europe	forest	absence	16	point count
1762	Switzerland	Europe	forest	absence	17	point count
1763	Switzerland	Europe	forest	absence	22	point count
1764	Switzerland	Europe	forest	presence	16	point count
1765	Switzerland	Europe	forest	absence	17	point count
1766	Switzerland	Europe	forest	absence	20	point count
1767	Switzerland	Europe	forest	absence	16	point count
1768	Switzerland	Europe	forest	absence	17	point count
1769	Switzerland	Europe	forest	absence	19	point count
1770	Switzerland	Europe	forest	absence	16	point count
1771	Switzerland	Europe	forest	absence	17	point count
1772	Switzerland	Europe	forest	absence	17	point count
1773	Switzerland	Europe	forest	absence	14	point count
1774	Switzerland	Europe	forest	absence	13	point count
1775	Switzerland	Europe	forest	absence	20	point count
1776	Switzerland	Europe	forest	absence	15	point count
1777	Switzerland	Europe	forest	absence	17	point count
1778	Switzerland	Europe	forest	absence	23	point count
1779	Switzerland	Europe	forest	absence	17	point count
1780	Switzerland	Europe	forest	absence	21	point count
1781	Switzerland	Europe	forest	absence	17	point count
1782	Switzerland	Europe	forest	absence	14	point count
1783	Switzerland	Europe	forest	absence	17	point count
1784	Switzerland	Europe	forest	presence	16	point count
1785	Switzerland	Europe	forest	presence	17	point count
1786	Switzerland	Europe	forest	absence	14	point count
1787	Switzerland	Europe	forest	absence	16	point count
1788	Switzerland	Europe	forest	absence	18	point count
1789	Switzerland	Europe	forest	absence	16	point count
1790	Switzerland	Europe	forest	absence	14	point count
1791	Switzerland	Europe	forest	absence	19	point count
1792	Switzerland	Europe	forest	absence	16	point count
1793	Switzerland	Europe	forest	presence	17	point count
1794	Switzerland	Europe	forest	absence	18	point count
1795	Switzerland	Europe	forest	absence	12	point count
1796	Switzerland	Europe	forest	absence	21	point count
1797	Switzerland	Europe	forest	absence	11	point count
1798	Switzerland	Europe	forest	absence	12	point count
1799	Switzerland	Europe	forest	absence	21	point count

1800	Switzerland	Europe	forest	absence	16	point count
1801	Switzerland	Europe	forest	absence	17	point count
1802	Switzerland	Europe	forest	absence	13	point count
1803	Switzerland	Europe	forest	absence	16	point count
1804	Switzerland	Europe	forest	absence	13	point count
1805	Switzerland	Europe	forest	absence	15	point count
1806	Switzerland	Europe	forest	absence	15	point count
1807	Switzerland	Europe	forest	absence	14	point count
1808	Switzerland	Europe	forest	absence	13	point count
1809	Switzerland	Europe	forest	absence	21	point count
1810	Switzerland	Europe	forest	presence	17	point count
1811	Switzerland	Europe	forest	absence	19	point count
1812	Switzerland	Europe	forest	presence	18	point count
1813	Switzerland	Europe	forest	absence	15	point count
1814	Switzerland	Europe	forest	absence	13	point count
1815	Switzerland	Europe	forest	absence	16	point count
1816	Switzerland	Europe	forest	presence	16	point count
1817	Switzerland	Europe	forest	presence	20	point count
1818	Switzerland	Europe	forest	absence	10	point count
1819	Ukraine	Europe	forest	presence	13	point count
1820	Ukraine	Europe	forest	absence	4	point count
1821	Ukraine	Europe	forest	presence	9	point count
1822	Ukraine	Europe	forest	absence	4	point count
1823	Ukraine	Europe	forest	presence	5	point count
1824	Ukraine	Europe	forest	absence	4	point count
1825	Ukraine	Europe	forest	presence	6	point count
1826	Ukraine	Europe	forest	absence	8	point count
1827	Ukraine	Europe	forest	presence	6	point count
1828	Ukraine	Europe	forest	absence	3	point count
1829	Ukraine	Europe	forest	presence	7	point count
1830	Ukraine	Europe	forest	absence	4	point count
1831	Ukraine	Europe	forest	presence	8	point count
1832	Ukraine	Europe	forest	absence	3	point count
1833	Ukraine	Europe	forest	presence	8	point count
1834	Ukraine	Europe	forest	absence	4	point count
1835	Ukraine	Europe	forest	presence	7	point count
1836	Ukraine	Europe	forest	absence	4	point count
1837	Ukraine	Europe	forest	presence	5	point count
1838	Ukraine	Europe	forest	absence	4	point count
1839	Ukraine	Europe	forest	presence	4	point count
1840	Ukraine	Europe	forest	absence	5	point count
1841	Ukraine	Europe	forest	presence	4	point count
1842	Ukraine	Europe	forest	absence	3	point count
1843	Ukraine	Europe	forest	presence	7	point count
1844	Ukraine	Europe	forest	absence	2	point count
1845	Ukraine	Europe	forest	presence	8	point count
1846	Ukraine	Europe	forest	absence	7	point count
1847	Ukraine	Europe	forest	presence	8	point count
1848	Ukraine	Europe	forest	absence	7	point count
1849	Ukraine	Europe	forest	presence	5	point count

1850	Ukraine	Europe	forest	absence	5	point count
1851	Ukraine	Europe	forest	presence	7	point count
1852	Ukraine	Europe	forest	absence	8	point count
1853	Ukraine	Europe	forest	presence	9	point count
1854	Ukraine	Europe	forest	absence	5	point count
1855	Ukraine	Europe	forest	presence	7	point count
1856	Ukraine	Europe	forest	absence	2	point count
1857	Ukraine	Europe	forest	presence	4	point count
1858	Ukraine	Europe	forest	absence	3	point count
1859	Ukraine	Europe	forest	presence	7	point count
1860	Ukraine	Europe	forest	absence	5	point count
1861	Ukraine	Europe	forest	presence	7	point count
1862	Ukraine	Europe	forest	absence	5	point count
1863	Ukraine	Europe	forest	presence	4	point count
1864	Ukraine	Europe	forest	absence	5	point count
1865	Ukraine	Europe	forest	presence	4	point count
1866	Ukraine	Europe	forest	absence	5	point count
1867	Ukraine	Europe	forest	presence	6	point count
1868	Ukraine	Europe	forest	absence	5	point count
1869	Ukraine	Europe	forest	presence	5	point count
1870	Ukraine	Europe	forest	absence	5	point count
1871	Ukraine	Europe	forest	presence	4	point count
1872	Ukraine	Europe	forest	absence	5	point count
1873	Ukraine	Europe	forest	presence	6	point count
1874	Ukraine	Europe	forest	absence	4	point count
1875	Ukraine	Europe	forest	presence	5	point count
1876	Ukraine	Europe	forest	absence	5	point count
1877	Ukraine	Europe	forest	presence	5	point count
1878	Ukraine	Europe	forest	absence	4	point count
1879	Ukraine	Europe	forest	presence	5	point count
1880	Ukraine	Europe	forest	absence	5	point count
1881	Ukraine	Europe	forest	presence	5	point count
1882	Ukraine	Europe	forest	absence	4	point count
1883	Ukraine	Europe	forest	presence	6	point count
1884	Ukraine	Europe	forest	absence	4	point count
1885	Ukraine	Europe	forest	presence	4	point count
1886	Ukraine	Europe	forest	absence	4	point count
1887	Ukraine	Europe	forest	presence	6	point count
1888	Ukraine	Europe	forest	absence	4	point count
1889	Ukraine	Europe	forest	presence	3	point count
1890	Ukraine	Europe	forest	absence	4	point count
1891	Ukraine	Europe	forest	presence	3	point count
1892	Ukraine	Europe	forest	absence	4	point count
1893	Ukraine	Europe	forest	presence	6	point count
1894	Ukraine	Europe	forest	absence	4	point count
1895	Ukraine	Europe	forest	presence	6	point count
1896	Ukraine	Europe	forest	absence	4	point count
1897	Ukraine	Europe	forest	presence	6	point count
1898	Ukraine	Europe	forest	absence	4	point count
1899	Ukraine	Europe	forest	presence	6	point count

1900	Ukraine	Europe	forest	absence	4	point count
1901	Ukraine	Europe	forest	presence	8	point count
1902	Ukraine	Europe	forest	absence	5	point count
1903	Ukraine	Europe	forest	presence	7	point count
1904	Ukraine	Europe	forest	absence	5	point count
1905	Ukraine	Europe	forest	presence	8	point count
1906	Ukraine	Europe	forest	absence	2	point count
1907	Ukraine	Europe	forest	presence	7	point count
1908	Ukraine	Europe	forest	absence	5	point count
1909	Ukraine	Europe	forest	presence	6	point count
1910	Ukraine	Europe	forest	absence	3	point count
1911	Ukraine	Europe	forest	presence	6	point count
1912	Ukraine	Europe	forest	absence	6	point count
1913	Ukraine	Europe	forest	absence	7	point count
1914	Ukraine	Europe	forest	presence	5	point count
1915	Ukraine	Europe	forest	presence	4	point count
1916	Ukraine	Europe	forest	absence	3	point count
1917	Ukraine	Europe	forest	presence	4	point count
1918	Ukraine	Europe	forest	absence	5	point count
1919	Ukraine	Europe	forest	presence	4	point count
1920	Ukraine	Europe	forest	absence	4	point count
1921	Ukraine	Europe	forest	presence	4	point count
1922	Ukraine	Europe	forest	absence	4	point count
1923	Ukraine	Europe	forest	presence	4	point count
1924	Ukraine	Europe	forest	absence	4	point count
1925	Ukraine	Europe	forest	presence	3	point count
1926	Ukraine	Europe	forest	presence	3	point count
1927	Ukraine	Europe	forest	presence	3	point count
1928	Ukraine	Europe	forest	absence	1	point count
1929	Ukraine	Europe	forest	presence	3	point count
1930	Ukraine	Europe	forest	absence	1	point count
1931	Ukraine	Europe	forest	presence	4	point count
1932	Ukraine	Europe	forest	absence	2	point count
1933	Ukraine	Europe	forest	presence	3	point count
1934	Ukraine	Europe	forest	absence	4	point count
1935	Ukraine	Europe	forest	presence	3	point count
1936	Ukraine	Europe	forest	absence	3	point count
1937	Ukraine	Europe	forest	presence	5	point count
1938	Ukraine	Europe	forest	absence	2	point count
1939	Ukraine	Europe	forest	presence	5	point count
1940	Ukraine	Europe	forest	absence	1	point count
1941	Ukraine	Europe	forest	presence	2	point count
1942	Ukraine	Europe	forest	absence	1	point count
1943	Ukraine	Europe	forest	presence	8	point count
1944	Ukraine	Europe	forest	absence	2	point count
1945	Ukraine	Europe	forest	presence	10	point count
1946	Ukraine	Europe	forest	absence	3	point count
1947	Ukraine	Europe	forest	presence	10	point count
1948	Ukraine	Europe	forest	absence	3	point count
1949	Ukraine	Europe	forest	presence	7	point count

1950	Ukraine	Europe	forest	absence	3	point count
1951	Ukraine	Europe	forest	presence	9	point count
1952	Ukraine	Europe	forest	absence	3	point count
1953	Ukraine	Europe	forest	presence	10	point count
1954	Ukraine	Europe	forest	absence	3	point count
1955	Ukraine	Europe	forest	presence	6	point count
1956	Ukraine	Europe	forest	absence	3	point count
1957	Ukraine	Europe	forest	presence	8	point count
1958	Ukraine	Europe	forest	absence	3	point count
1959	Ukraine	Europe	forest	presence	10	point count
1960	Ukraine	Europe	forest	absence	8	point count
1961	Ukraine	Europe	forest	presence	8	point count
1962	Ukraine	Europe	forest	absence	3	point count
1963	Ukraine	Europe	forest	presence	9	point count
1964	Ukraine	Europe	forest	absence	7	point count
1965	Ukraine	Europe	forest	presence	10	point count
1966	Ukraine	Europe	forest	absence	6	point count
1967	Ukraine	Europe	forest	presence	8	point count
1968	Ukraine	Europe	forest	absence	7	point count
1969	Ukraine	Europe	forest	presence	8	point count
1970	Ukraine	Europe	forest	absence	7	point count
1971	Ukraine	Europe	forest	presence	7	point count
1972	Ukraine	Europe	forest	absence	3	point count
1973	Ukraine	Europe	forest	presence	7	point count
1974	Ukraine	Europe	forest	absence	3	point count
1975	Ukraine	Europe	forest	presence	7	point count
1976	Ukraine	Europe	forest	absence	3	point count
1977	Ukraine	Europe	forest	presence	8	point count
1978	Ukraine	Europe	forest	absence	5	point count
1979	Ukraine	Europe	forest	presence	8	point count
1980	Ukraine	Europe	forest	absence	5	point count
1981	Ukraine	Europe	forest	presence	4	point count
1982	Ukraine	Europe	forest	absence	3	point count
1983	Ukraine	Europe	forest	presence	4	point count
1984	Ukraine	Europe	forest	absence	3	point count
1985	Ukraine	Europe	forest	presence	4	point count
1986	Ukraine	Europe	forest	absence	3	point count
1987	Ukraine	Europe	forest	presence	7	point count
1988	Ukraine	Europe	forest	absence	7	point count
1989	Ukraine	Europe	forest	presence	8	point count
1990	Ukraine	Europe	forest	absence	6	point count
1991	Ukraine	Europe	forest	presence	9	point count
1992	Ukraine	Europe	forest	absence	3	point count
1993	Ukraine	Europe	forest	presence	9	point count
1994	Ukraine	Europe	forest	absence	6	point count
1995	Ukraine	Europe	forest	presence	7	point count
1996	Ukraine	Europe	forest	absence	6	point count
1997	Ukraine	Europe	forest	presence	6	point count
1998	Ukraine	Europe	forest	absence	6	point count
1999	Ukraine	Europe	forest	presence	9	point count

2000	Ukraine	Europe	forest	absence	3	point count
2001	Ukraine	Europe	forest	presence	7	point count
2002	Ukraine	Europe	forest	absence	3	point count
2003	Ukraine	Europe	forest	presence	7	point count
2004	Ukraine	Europe	forest	absence	3	point count
2005	Ukraine	Europe	forest	presence	5	point count
2006	Ukraine	Europe	forest	absence	3	point count
2007	Ukraine	Europe	forest	presence	7	point count
2008	Ukraine	Europe	forest	absence	3	point count
2009	Ukraine	Europe	forest	presence	7	point count
2010	Ukraine	Europe	forest	absence	2	point count
2011	Ukraine	Europe	forest	presence	8	point count
2012	Ukraine	Europe	forest	absence	2	point count
2013	Ukraine	Europe	forest	presence	8	point count
2014	Ukraine	Europe	forest	absence	2	point count
2015	Ukraine	Europe	forest	presence	8	point count
2016	Ukraine	Europe	forest	absence	3	point count
2017	Ukraine	Europe	forest	presence	4	point count
2018	Ukraine	Europe	forest	absence	3	point count
2019	Ukraine	Europe	forest	presence	10	point count
2020	Ukraine	Europe	forest	absence	6	point count
2021	Ukraine	Europe	forest	presence	6	point count
2022	Ukraine	Europe	forest	absence	6	point count
2023	Ukraine	Europe	forest	presence	6	point count
2024	Ukraine	Europe	forest	absence	6	point count
2025	Ukraine	Europe	forest	presence	7	point count
2026	Ukraine	Europe	forest	absence	5	point count
2027	Ukraine	Europe	forest	presence	6	point count
2028	Ukraine	Europe	forest	absence	6	point count
2029	Ukraine	Europe	forest	presence	4	point count
2030	Ukraine	Europe	forest	absence	5	point count
2031	Ukraine	Europe	forest	presence	4	point count
2032	Ukraine	Europe	forest	absence	3	point count
2033	Ukraine	Europe	forest	presence	6	point count
2034	Ukraine	Europe	forest	absence	3	point count
2035	Ukraine	Europe	forest	presence	6	point count
2036	Ukraine	Europe	forest	absence	3	point count
2037	Ukraine	Europe	forest	presence	5	point count
2038	Ukraine	Europe	forest	absence	3	point count
2039	Ukraine	Europe	forest	presence	5	point count
2040	Ukraine	Europe	forest	absence	4	point count
2041	Ukraine	Europe	forest	presence	4	point count
2042	Ukraine	Europe	forest	absence	4	point count
2043	Ukraine	Europe	forest	presence	4	point count
2044	Ukraine	Europe	forest	absence	4	point count
2045	Ukraine	Europe	forest	presence	15	point count
2046	Ukraine	Europe	forest	absence	6	point count
2047	Ukraine	Europe	forest	presence	12	point count
2048	Ukraine	Europe	forest	absence	8	point count
2049	Ukraine	Europe	forest	presence	8	point count

2050	Ukraine	Europe	forest	absence	6	point count
2051	Ukraine	Europe	forest	presence	8	point count
2052	Ukraine	Europe	forest	absence	6	point count
2053	Ukraine	Europe	forest	presence	8	point count
2054	Ukraine	Europe	forest	absence	6	point count
2055	Ukraine	Europe	forest	presence	7	point count
2056	Ukraine	Europe	forest	absence	6	point count
2057	Ukraine	Europe	forest	presence	7	point count
2058	Ukraine	Europe	forest	absence	6	point count
2059	Ukraine	Europe	forest	presence	8	point count
2060	Ukraine	Europe	forest	absence	6	point count
2061	Ukraine	Europe	forest	presence	8	point count
2062	Ukraine	Europe	forest	absence	6	point count
2063	Ukraine	Europe	forest	presence	8	point count
2064	Ukraine	Europe	forest	absence	8	point count
2065	Ukraine	Europe	forest	presence	4	point count
2066	Ukraine	Europe	forest	absence	9	point count
2067	Ukraine	Europe	forest	presence	6	point count
2068	Ukraine	Europe	forest	absence	7	point count
2069	Ukraine	Europe	forest	presence	8	point count
2070	Ukraine	Europe	forest	absence	6	point count
2071	Ukraine	Europe	forest	presence	12	point count
2072	Ukraine	Europe	forest	absence	7	point count
2073	Ukraine	Europe	forest	presence	8	point count
2074	Ukraine	Europe	forest	absence	10	point count
2075	Ukraine	Europe	forest	presence	9	point count
2076	Ukraine	Europe	forest	absence	3	point count
2077	France	Europe	farmland	presence	46	squares
2078	France	Europe	farmland	presence	38	squares
2079	France	Europe	farmland	presence	46	squares
2080	France	Europe	farmland	presence	41	squares
2081	France	Europe	farmland	presence	32	squares
2082	France	Europe	farmland	presence	30	squares
2083	France	Europe	farmland	presence	44	squares
2084	France	Europe	farmland	absence	42	squares
2085	France	Europe	farmland	presence	25	squares
2086	France	Europe	farmland	presence	40	squares
2087	France	Europe	farmland	presence	29	squares
2088	France	Europe	farmland	presence	34	squares
2089	France	Europe	farmland	absence	37	squares
2090	France	Europe	farmland	presence	30	squares
2091	France	Europe	farmland	presence	40	squares
2092	France	Europe	farmland	presence	42	squares
2093	France	Europe	farmland	presence	29	squares
2094	France	Europe	farmland	absence	38	squares
2095	France	Europe	farmland	presence	36	squares
2096	France	Europe	farmland	absence	43	squares
2097	France	Europe	farmland	presence	44	squares
2098	France	Europe	farmland	absence	34	squares
2099	France	Europe	farmland	presence	36	squares

2100	France	Europe	farmland	presence	40 squares
2101	France	Europe	farmland	presence	52 squares
2102	France	Europe	farmland	presence	47 squares
2103	France	Europe	farmland	presence	50 squares
2104	France	Europe	farmland	presence	35 squares
2105	France	Europe	farmland	presence	28 squares
2106	France	Europe	farmland	presence	29 squares
2107	France	Europe	farmland	presence	48 squares
2108	France	Europe	farmland	presence	39 squares
2109	France	Europe	farmland	presence	32 squares
2110	France	Europe	farmland	presence	52 squares
2111	France	Europe	farmland	absence	21 squares
2112	France	Europe	farmland	presence	40 squares
2113	France	Europe	farmland	presence	44 squares
2114	France	Europe	farmland	presence	44 squares
2115	France	Europe	farmland	presence	46 squares
2116	France	Europe	farmland	presence	44 squares
2117	France	Europe	farmland	presence	39 squares
2118	France	Europe	farmland	presence	42 squares
2119	France	Europe	farmland	presence	35 squares
2120	France	Europe	farmland	presence	43 squares
2121	France	Europe	farmland	absence	38 squares
2122	France	Europe	farmland	presence	54 squares
2123	France	Europe	farmland	presence	37 squares
2124	France	Europe	farmland	presence	38 squares
2125	France	Europe	farmland	presence	26 squares
2126	France	Europe	farmland	absence	23 squares
2127	France	Europe	farmland	presence	18 squares
2128	France	Europe	farmland	presence	18 squares
2129	France	Europe	farmland	presence	35 squares
2130	France	Europe	farmland	presence	39 squares
2131	France	Europe	farmland	presence	38 squares
2132	France	Europe	farmland	presence	43 squares
2133	France	Europe	farmland	presence	42 squares
2134	France	Europe	farmland	presence	31 squares
2135	France	Europe	farmland	presence	32 squares
2136	France	Europe	farmland	absence	19 squares
2137	France	Europe	farmland	absence	18 squares
2138	France	Europe	farmland	absence	29 squares
2139	France	Europe	farmland	presence	29 squares
2140	France	Europe	farmland	presence	44 squares
2141	France	Europe	farmland	presence	37 squares
2142	France	Europe	farmland	presence	38 squares
2143	France	Europe	farmland	presence	34 squares
2144	France	Europe	farmland	presence	42 squares
2145	France	Europe	farmland	presence	30 squares
2146	France	Europe	farmland	presence	37 squares
2147	France	Europe	farmland	presence	32 squares
2148	France	Europe	farmland	presence	48 squares
2149	France	Europe	farmland	presence	40 squares

2150	France	Europe	farmland	presence	40 squares
2151	France	Europe	farmland	presence	39 squares
2152	France	Europe	farmland	presence	28 squares
2153	France	Europe	farmland	presence	36 squares
2154	France	Europe	farmland	presence	38 squares
2155	France	Europe	farmland	presence	43 squares
2156	France	Europe	farmland	presence	47 squares
2157	France	Europe	farmland	presence	35 squares
2158	France	Europe	farmland	presence	46 squares
2159	France	Europe	farmland	absence	41 squares
2160	France	Europe	farmland	absence	24 squares
2161	France	Europe	farmland	presence	36 squares
2162	France	Europe	farmland	presence	43 squares
2163	France	Europe	farmland	presence	26 squares
2164	France	Europe	farmland	presence	44 squares
2165	France	Europe	farmland	presence	44 squares
2166	France	Europe	farmland	presence	45 squares
2167	France	Europe	farmland	presence	41 squares
2168	France	Europe	farmland	absence	25 squares
2169	France	Europe	farmland	presence	40 squares
2170	France	Europe	farmland	absence	28 squares
2171	France	Europe	farmland	absence	26 squares
2172	France	Europe	farmland	presence	22 squares
2173	France	Europe	farmland	absence	43 squares
2174	France	Europe	farmland	absence	40 squares
2175	France	Europe	farmland	presence	41 squares
2176	France	Europe	farmland	absence	50 squares
2177	France	Europe	farmland	presence	51 squares
2178	France	Europe	farmland	presence	39 squares
2179	France	Europe	farmland	presence	30 squares
2180	France	Europe	farmland	presence	33 squares
2181	France	Europe	farmland	absence	33 squares
2182	France	Europe	farmland	absence	26 squares
2183	France	Europe	farmland	presence	23 squares
2184	France	Europe	farmland	absence	32 squares
2185	France	Europe	farmland	presence	29 squares
2186	France	Europe	farmland	presence	35 squares
2187	France	Europe	farmland	presence	37 squares
2188	France	Europe	farmland	absence	35 squares
2189	France	Europe	farmland	presence	46 squares
2190	France	Europe	farmland	presence	42 squares
2191	France	Europe	farmland	absence	33 squares
2192	France	Europe	farmland	presence	50 squares
2193	France	Europe	farmland	presence	35 squares
2194	France	Europe	farmland	presence	37 squares
2195	France	Europe	farmland	presence	32 squares
2196	France	Europe	farmland	presence	28 squares
2197	France	Europe	farmland	absence	24 squares
2198	France	Europe	farmland	absence	38 squares
2199	France	Europe	farmland	absence	30 squares

2200	France	Europe	farmland	presence	32	squares
2201	France	Europe	farmland	presence	23	squares
2202	France	Europe	farmland	presence	29	squares
2203	France	Europe	farmland	absence	35	squares
2204	France	Europe	farmland	absence	33	squares
2205	France	Europe	farmland	absence	14	squares
2206	France	Europe	farmland	presence	32	squares
2207	France	Europe	farmland	presence	30	squares
2208	France	Europe	farmland	absence	19	squares
2209	France	Europe	farmland	presence	27	squares
2210	France	Europe	farmland	presence	31	squares
2211	France	Europe	farmland	presence	16	squares
2212	France	Europe	farmland	absence	14	squares
2213	France	Europe	farmland	absence	35	squares
2214	France	Europe	farmland	absence	28	squares
2215	France	Europe	farmland	absence	28	squares
2216	France	Europe	farmland	presence	33	squares
2217	France	Europe	farmland	presence	40	squares
2218	France	Europe	farmland	absence	32	squares
2219	France	Europe	farmland	absence	38	squares
2220	France	Europe	farmland	presence	38	squares
2221	France	Europe	farmland	absence	25	squares
2222	France	Europe	farmland	absence	37	squares
2223	France	Europe	farmland	presence	42	squares
2224	France	Europe	farmland	presence	43	squares
2225	France	Europe	farmland	absence	27	squares
2226	France	Europe	farmland	absence	29	squares
2227	France	Europe	farmland	presence	36	squares
2228	France	Europe	farmland	presence	56	squares
2229	France	Europe	farmland	absence	18	squares
2230	France	Europe	farmland	presence	37	squares
2231	France	Europe	farmland	presence	41	squares
2232	France	Europe	farmland	presence	38	squares
2233	France	Europe	farmland	presence	45	squares
2234	France	Europe	farmland	presence	41	squares
2235	France	Europe	farmland	presence	58	squares
2236	France	Europe	farmland	presence	40	squares
2237	France	Europe	farmland	presence	45	squares
2238	France	Europe	farmland	presence	40	squares
2239	France	Europe	farmland	presence	39	squares
2240	France	Europe	farmland	absence	39	squares
2241	France	Europe	farmland	presence	39	squares
2242	France	Europe	farmland	presence	43	squares
2243	France	Europe	farmland	presence	29	squares
2244	France	Europe	farmland	presence	36	squares
2245	France	Europe	farmland	presence	44	squares
2246	France	Europe	farmland	presence	42	squares
2247	France	Europe	farmland	presence	35	squares
2248	France	Europe	farmland	absence	38	squares
2249	France	Europe	farmland	presence	33	squares

2250	France	Europe	farmland	presence	53	squares
2251	France	Europe	farmland	presence	32	squares
2252	France	Europe	farmland	presence	31	squares
2253	France	Europe	farmland	presence	34	squares
2254	France	Europe	farmland	presence	42	squares
2255	France	Europe	farmland	absence	20	squares
2256	France	Europe	farmland	presence	37	squares
2257	France	Europe	farmland	presence	50	squares
2258	France	Europe	farmland	presence	46	squares
2259	France	Europe	farmland	presence	55	squares
2260	France	Europe	farmland	presence	42	squares
2261	France	Europe	farmland	presence	46	squares
2262	France	Europe	farmland	presence	39	squares
2263	France	Europe	farmland	presence	38	squares
2264	France	Europe	farmland	presence	44	squares
2265	France	Europe	farmland	presence	40	squares
2266	France	Europe	farmland	presence	48	squares
2267	France	Europe	farmland	presence	35	squares
2268	France	Europe	farmland	presence	34	squares
2269	France	Europe	farmland	presence	46	squares
2270	France	Europe	farmland	presence	42	squares
2271	France	Europe	farmland	absence	32	squares
2272	France	Europe	farmland	presence	29	squares
2273	France	Europe	farmland	presence	39	squares
2274	France	Europe	farmland	absence	26	squares
2275	France	Europe	farmland	presence	46	squares
2276	France	Europe	farmland	presence	33	squares
2277	France	Europe	farmland	presence	33	squares
2278	France	Europe	farmland	presence	41	squares
2279	France	Europe	farmland	presence	36	squares
2280	France	Europe	farmland	presence	33	squares
2281	France	Europe	farmland	presence	31	squares
2282	France	Europe	farmland	presence	39	squares
2283	France	Europe	farmland	presence	33	squares
2284	France	Europe	farmland	presence	30	squares
2285	France	Europe	farmland	presence	46	squares
2286	France	Europe	farmland	presence	39	squares
2287	France	Europe	farmland	presence	32	squares
2288	France	Europe	farmland	absence	29	squares
2289	France	Europe	farmland	presence	26	squares
2290	France	Europe	farmland	presence	30	squares
2291	France	Europe	farmland	presence	30	squares
2292	France	Europe	farmland	absence	29	squares
2293	France	Europe	farmland	absence	39	squares
2294	France	Europe	farmland	absence	42	squares
2295	France	Europe	farmland	presence	31	squares
2296	France	Europe	farmland	absence	31	squares
2297	France	Europe	farmland	presence	30	squares
2298	France	Europe	farmland	presence	33	squares
2299	France	Europe	farmland	presence	26	squares

2300	France	Europe	farmland	absence	30 squares
2301	France	Europe	farmland	presence	27 squares
2302	France	Europe	farmland	absence	40 squares
2303	France	Europe	farmland	presence	28 squares
2304	France	Europe	farmland	absence	52 squares
2305	France	Europe	farmland	presence	31 squares
2306	France	Europe	farmland	presence	39 squares
2307	France	Europe	farmland	absence	26 squares
2308	France	Europe	farmland	absence	23 squares
2309	France	Europe	farmland	presence	34 squares
2310	France	Europe	farmland	absence	25 squares
2311	France	Europe	farmland	absence	26 squares
2312	France	Europe	farmland	presence	39 squares
2313	France	Europe	farmland	absence	30 squares
2314	France	Europe	farmland	presence	32 squares
2315	France	Europe	farmland	presence	29 squares
2316	France	Europe	farmland	absence	30 squares
2317	France	Europe	farmland	absence	25 squares
2318	France	Europe	farmland	presence	33 squares
2319	France	Europe	farmland	presence	33 squares
2320	France	Europe	farmland	presence	23 squares
2321	France	Europe	farmland	presence	32 squares
2322	France	Europe	farmland	presence	49 squares
2323	France	Europe	farmland	presence	38 squares
2324	France	Europe	farmland	presence	30 squares
2325	France	Europe	farmland	presence	35 squares
2326	France	Europe	farmland	presence	37 squares
2327	France	Europe	farmland	presence	38 squares
2328	France	Europe	farmland	presence	43 squares
2329	France	Europe	farmland	presence	46 squares
2330	France	Europe	farmland	presence	43 squares
2331	France	Europe	farmland	presence	45 squares
2332	France	Europe	farmland	presence	41 squares
2333	France	Europe	farmland	presence	45 squares
2334	France	Europe	farmland	absence	25 squares
2335	France	Europe	farmland	presence	44 squares
2336	France	Europe	farmland	presence	35 squares
2337	France	Europe	farmland	presence	45 squares
2338	France	Europe	farmland	presence	45 squares
2339	France	Europe	farmland	presence	54 squares
2340	France	Europe	farmland	presence	39 squares
2341	France	Europe	farmland	presence	35 squares
2342	France	Europe	farmland	presence	39 squares
2343	France	Europe	farmland	presence	46 squares
2344	France	Europe	farmland	presence	29 squares
2345	France	Europe	farmland	presence	39 squares
2346	France	Europe	farmland	absence	43 squares
2347	France	Europe	farmland	presence	41 squares
2348	France	Europe	farmland	presence	40 squares
2349	France	Europe	farmland	presence	40 squares

2350	France	Europe	farmland	presence	30 squares
2351	France	Europe	farmland	absence	23 squares
2352	France	Europe	farmland	presence	29 squares
2353	France	Europe	farmland	absence	36 squares
2354	France	Europe	farmland	presence	19 squares
2355	France	Europe	farmland	presence	23 squares
2356	France	Europe	farmland	absence	18 squares
2357	France	Europe	farmland	absence	18 squares
2358	France	Europe	farmland	presence	41 squares
2359	France	Europe	farmland	presence	42 squares
2360	France	Europe	farmland	presence	36 squares
2361	France	Europe	farmland	absence	25 squares
2362	France	Europe	farmland	presence	33 squares
2363	France	Europe	farmland	presence	36 squares
2364	France	Europe	farmland	presence	39 squares
2365	France	Europe	farmland	presence	32 squares
2366	France	Europe	farmland	presence	24 squares
2367	France	Europe	farmland	presence	29 squares
2368	France	Europe	farmland	presence	27 squares
2369	France	Europe	farmland	presence	31 squares
2370	France	Europe	farmland	presence	27 squares
2371	France	Europe	farmland	presence	41 squares
2372	France	Europe	farmland	absence	39 squares
2373	France	Europe	farmland	presence	32 squares
2374	France	Europe	farmland	presence	38 squares
2375	France	Europe	farmland	presence	29 squares
2376	France	Europe	farmland	presence	33 squares
2377	France	Europe	farmland	presence	37 squares
2378	France	Europe	farmland	absence	42 squares
2379	France	Europe	farmland	presence	47 squares
2380	France	Europe	farmland	presence	42 squares
2381	France	Europe	farmland	presence	44 squares
2382	France	Europe	farmland	presence	27 squares
2383	France	Europe	farmland	presence	46 squares
2384	France	Europe	farmland	presence	41 squares
2385	France	Europe	farmland	presence	39 squares
2386	France	Europe	farmland	presence	43 squares
2387	France	Europe	farmland	presence	34 squares
2388	France	Europe	farmland	presence	38 squares
2389	France	Europe	farmland	presence	39 squares
2390	France	Europe	farmland	presence	37 squares
2391	France	Europe	farmland	absence	29 squares
2392	France	Europe	farmland	absence	27 squares
2393	France	Europe	farmland	absence	38 squares
2394	France	Europe	farmland	absence	34 squares
2395	France	Europe	farmland	absence	25 squares
2396	France	Europe	farmland	absence	35 squares
2397	France	Europe	farmland	absence	27 squares
2398	France	Europe	farmland	absence	22 squares
2399	France	Europe	farmland	absence	40 squares

2400	France	Europe	farmland	absence	21 squares
2401	France	Europe	farmland	presence	31 squares
2402	France	Europe	farmland	presence	28 squares
2403	France	Europe	farmland	presence	27 squares
2404	France	Europe	farmland	absence	26 squares
2405	France	Europe	farmland	presence	30 squares
2406	France	Europe	farmland	absence	40 squares
2407	France	Europe	farmland	absence	30 squares
2408	France	Europe	farmland	presence	26 squares
2409	France	Europe	farmland	presence	48 squares
2410	France	Europe	farmland	presence	46 squares
2411	France	Europe	farmland	presence	35 squares
2412	France	Europe	farmland	presence	36 squares
2413	France	Europe	farmland	presence	36 squares
2414	France	Europe	farmland	presence	31 squares
2415	France	Europe	farmland	presence	39 squares
2416	France	Europe	farmland	absence	18 squares
2417	France	Europe	farmland	absence	30 squares
2418	France	Europe	farmland	presence	37 squares
2419	France	Europe	farmland	presence	46 squares
2420	France	Europe	farmland	presence	28 squares
2421	France	Europe	farmland	absence	24 squares
2422	France	Europe	farmland	presence	43 squares
2423	France	Europe	farmland	presence	42 squares
2424	France	Europe	farmland	presence	33 squares
2425	France	Europe	farmland	presence	43 squares
2426	France	Europe	farmland	presence	34 squares
2427	France	Europe	farmland	presence	26 squares
2428	France	Europe	farmland	presence	27 squares
2429	France	Europe	farmland	presence	19 squares
2430	France	Europe	farmland	presence	30 squares
2431	France	Europe	farmland	presence	29 squares
2432	France	Europe	farmland	presence	33 squares
2433	France	Europe	farmland	presence	31 squares
2434	France	Europe	farmland	absence	24 squares
2435	France	Europe	farmland	presence	40 squares
2436	France	Europe	farmland	absence	33 squares
2437	France	Europe	farmland	presence	24 squares
2438	France	Europe	farmland	absence	27 squares
2439	France	Europe	farmland	presence	36 squares
2440	France	Europe	farmland	presence	27 squares
2441	France	Europe	farmland	absence	35 squares
2442	France	Europe	farmland	absence	35 squares
2443	France	Europe	farmland	absence	25 squares
2444	France	Europe	farmland	presence	35 squares
2445	France	Europe	farmland	presence	36 squares
2446	France	Europe	farmland	presence	31 squares
2447	France	Europe	farmland	presence	41 squares
2448	France	Europe	farmland	presence	27 squares
2449	France	Europe	farmland	presence	32 squares

2450	France	Europe	farmland	absence	29	squares
2451	France	Europe	farmland	presence	27	squares
2452	France	Europe	farmland	presence	37	squares
2453	France	Europe	farmland	presence	36	squares
2454	France	Europe	farmland	presence	33	squares
2455	France	Europe	farmland	presence	37	squares
2456	France	Europe	farmland	presence	35	squares
2457	France	Europe	farmland	presence	43	squares
2458	France	Europe	farmland	presence	38	squares
2459	France	Europe	farmland	presence	35	squares
2460	France	Europe	farmland	presence	44	squares
2461	France	Europe	farmland	presence	37	squares
2462	France	Europe	farmland	presence	32	squares
2463	France	Europe	farmland	presence	45	squares
2464	France	Europe	farmland	presence	46	squares
2465	France	Europe	farmland	presence	46	squares
2466	France	Europe	farmland	presence	44	squares
2467	France	Europe	farmland	presence	40	squares
2468	France	Europe	farmland	presence	50	squares
2469	France	Europe	farmland	presence	29	squares
2470	France	Europe	farmland	presence	40	squares
2471	France	Europe	farmland	presence	42	squares
2472	France	Europe	farmland	presence	41	squares
2473	France	Europe	farmland	presence	36	squares
2474	France	Europe	farmland	absence	38	squares
2475	France	Europe	farmland	presence	36	squares
2476	France	Europe	farmland	presence	32	squares
2477	France	Europe	farmland	presence	39	squares
2478	France	Europe	farmland	presence	30	squares
2479	France	Europe	farmland	absence	38	squares
2480	France	Europe	farmland	presence	33	squares
2481	France	Europe	farmland	absence	36	squares
2482	France	Europe	farmland	absence	25	squares
2483	France	Europe	farmland	presence	23	squares
2484	France	Europe	farmland	presence	20	squares
2485	France	Europe	farmland	presence	41	squares
2486	France	Europe	farmland	absence	38	squares
2487	France	Europe	farmland	presence	23	squares
2488	France	Europe	farmland	absence	29	squares
2489	France	Europe	farmland	presence	38	squares
2490	France	Europe	farmland	absence	34	squares
2491	France	Europe	farmland	absence	35	squares
2492	France	Europe	farmland	presence	33	squares
2493	France	Europe	farmland	presence	37	squares
2494	France	Europe	farmland	presence	36	squares
2495	France	Europe	farmland	presence	23	squares
2496	France	Europe	farmland	presence	32	squares
2497	France	Europe	farmland	presence	33	squares
2498	France	Europe	farmland	presence	26	squares
2499	France	Europe	farmland	presence	35	squares

2500	France	Europe	farmland	presence	22 squares
2501	France	Europe	farmland	presence	36 squares
2502	France	Europe	farmland	presence	34 squares
2503	France	Europe	farmland	presence	40 squares
2504	France	Europe	farmland	presence	20 squares
2505	France	Europe	farmland	absence	38 squares
2506	France	Europe	farmland	presence	36 squares
2507	France	Europe	farmland	presence	36 squares
2508	France	Europe	farmland	presence	30 squares
2509	France	Europe	farmland	presence	31 squares
2510	France	Europe	farmland	presence	29 squares
2511	France	Europe	farmland	presence	30 squares
2512	France	Europe	farmland	presence	35 squares
2513	France	Europe	farmland	presence	42 squares
2514	France	Europe	farmland	presence	24 squares
2515	France	Europe	farmland	absence	25 squares
2516	France	Europe	farmland	absence	26 squares
2517	France	Europe	farmland	presence	42 squares
2518	France	Europe	farmland	presence	32 squares
2519	France	Europe	farmland	presence	44 squares
2520	France	Europe	farmland	presence	39 squares
2521	France	Europe	farmland	presence	26 squares
2522	France	Europe	farmland	presence	33 squares
2523	France	Europe	farmland	presence	29 squares
2524	France	Europe	farmland	presence	37 squares
2525	France	Europe	farmland	absence	30 squares
2526	France	Europe	farmland	presence	23 squares
2527	France	Europe	farmland	presence	32 squares
2528	France	Europe	farmland	presence	24 squares
2529	France	Europe	farmland	absence	28 squares
2530	France	Europe	farmland	presence	30 squares
2531	France	Europe	farmland	presence	34 squares
2532	France	Europe	farmland	presence	35 squares
2533	France	Europe	farmland	presence	51 squares
2534	France	Europe	farmland	presence	36 squares
2535	France	Europe	farmland	presence	46 squares
2536	France	Europe	farmland	presence	42 squares
2537	France	Europe	farmland	presence	42 squares
2538	France	Europe	farmland	presence	31 squares
2539	France	Europe	farmland	presence	47 squares
2540	France	Europe	farmland	presence	53 squares
2541	France	Europe	farmland	absence	41 squares
2542	France	Europe	farmland	presence	44 squares
2543	France	Europe	farmland	presence	50 squares
2544	France	Europe	farmland	presence	47 squares
2545	France	Europe	farmland	presence	49 squares
2546	France	Europe	farmland	presence	41 squares
2547	France	Europe	farmland	presence	49 squares
2548	France	Europe	farmland	presence	36 squares
2549	France	Europe	farmland	presence	48 squares

2550	France	Europe	farmland	presence	43	squares
2551	France	Europe	farmland	presence	45	squares
2552	France	Europe	farmland	presence	39	squares
2553	France	Europe	farmland	presence	54	squares
2554	France	Europe	farmland	presence	22	squares
2555	France	Europe	farmland	presence	50	squares
2556	France	Europe	farmland	presence	39	squares
2557	France	Europe	farmland	presence	40	squares
2558	France	Europe	farmland	presence	40	squares
2559	France	Europe	farmland	presence	46	squares
2560	France	Europe	farmland	presence	45	squares
2561	France	Europe	farmland	presence	43	squares
2562	France	Europe	farmland	absence	39	squares
2563	France	Europe	farmland	presence	44	squares
2564	France	Europe	farmland	presence	34	squares
2565	France	Europe	farmland	presence	47	squares
2566	France	Europe	farmland	presence	39	squares
2567	France	Europe	farmland	presence	37	squares
2568	France	Europe	farmland	presence	33	squares
2569	France	Europe	farmland	presence	42	squares
2570	France	Europe	farmland	presence	30	squares
2571	France	Europe	farmland	presence	41	squares
2572	France	Europe	farmland	presence	52	squares
2573	France	Europe	farmland	presence	40	squares
2574	France	Europe	farmland	presence	46	squares
2575	France	Europe	farmland	presence	35	squares
2576	France	Europe	farmland	presence	36	squares
2577	France	Europe	farmland	presence	34	squares
2578	France	Europe	farmland	presence	30	squares
2579	France	Europe	farmland	presence	35	squares
2580	France	Europe	farmland	absence	31	squares
2581	France	Europe	farmland	absence	39	squares
2582	France	Europe	farmland	presence	39	squares
2583	France	Europe	farmland	presence	31	squares
2584	France	Europe	farmland	presence	29	squares
2585	France	Europe	farmland	presence	29	squares
2586	France	Europe	farmland	presence	43	squares
2587	France	Europe	farmland	absence	33	squares
2588	France	Europe	farmland	presence	39	squares
2589	France	Europe	farmland	presence	33	squares
2590	France	Europe	farmland	presence	29	squares
2591	France	Europe	farmland	presence	33	squares
2592	France	Europe	farmland	presence	37	squares
2593	France	Europe	farmland	presence	34	squares
2594	France	Europe	farmland	presence	40	squares
2595	France	Europe	farmland	absence	21	squares
2596	France	Europe	farmland	presence	38	squares
2597	France	Europe	farmland	absence	38	squares
2598	France	Europe	farmland	presence	37	squares
2599	France	Europe	farmland	presence	50	squares

2600	France	Europe	farmland	presence	46	squares
2601	France	Europe	farmland	presence	38	squares
2602	France	Europe	farmland	presence	23	squares
2603	France	Europe	farmland	presence	17	squares
2604	France	Europe	farmland	presence	33	squares
2605	France	Europe	farmland	presence	35	squares
2606	France	Europe	farmland	presence	43	squares
2607	France	Europe	farmland	presence	35	squares
2608	France	Europe	farmland	presence	36	squares
2609	France	Europe	farmland	presence	32	squares
2610	France	Europe	farmland	absence	38	squares
2611	France	Europe	farmland	absence	29	squares
2612	France	Europe	farmland	presence	49	squares
2613	France	Europe	farmland	presence	32	squares
2614	France	Europe	farmland	presence	42	squares
2615	France	Europe	farmland	presence	48	squares
2616	France	Europe	farmland	presence	40	squares
2617	France	Europe	farmland	presence	23	squares
2618	France	Europe	farmland	presence	41	squares
2619	France	Europe	farmland	presence	20	squares
2620	France	Europe	farmland	presence	37	squares
2621	France	Europe	farmland	absence	27	squares
2622	France	Europe	farmland	presence	27	squares
2623	France	Europe	farmland	presence	40	squares
2624	France	Europe	farmland	presence	42	squares
2625	France	Europe	farmland	absence	34	squares
2626	France	Europe	farmland	absence	34	squares
2627	France	Europe	farmland	absence	40	squares
2628	France	Europe	farmland	absence	46	squares
2629	France	Europe	farmland	presence	48	squares
2630	France	Europe	farmland	presence	45	squares
2631	France	Europe	farmland	presence	40	squares
2632	France	Europe	farmland	absence	43	squares
2633	France	Europe	farmland	presence	50	squares
2634	France	Europe	farmland	presence	36	squares
2635	France	Europe	farmland	absence	40	squares
2636	France	Europe	farmland	absence	38	squares
2637	France	Europe	farmland	absence	35	squares
2638	France	Europe	farmland	absence	23	squares
2639	France	Europe	farmland	presence	44	squares
2640	France	Europe	farmland	presence	44	squares
2641	France	Europe	farmland	absence	37	squares
2642	France	Europe	farmland	presence	33	squares
2643	France	Europe	farmland	presence	45	squares
2644	France	Europe	farmland	presence	45	squares
2645	France	Europe	farmland	presence	32	squares
2646	France	Europe	farmland	presence	31	squares
2647	France	Europe	farmland	presence	37	squares
2648	France	Europe	farmland	presence	42	squares
2649	France	Europe	farmland	presence	38	squares

2650	France	Europe	farmland	presence	26 squares
2651	France	Europe	farmland	presence	30 squares
2652	France	Europe	farmland	presence	25 squares
2653	France	Europe	farmland	presence	48 squares
2654	France	Europe	farmland	absence	48 squares
2655	France	Europe	farmland	presence	35 squares
2656	France	Europe	farmland	presence	33 squares
2657	France	Europe	farmland	absence	47 squares
2658	France	Europe	farmland	presence	33 squares
2659	France	Europe	farmland	presence	33 squares
2660	France	Europe	farmland	presence	32 squares
2661	France	Europe	farmland	absence	30 squares
2662	France	Europe	farmland	absence	34 squares
2663	France	Europe	farmland	presence	47 squares
2664	France	Europe	farmland	absence	28 squares
2665	France	Europe	farmland	absence	50 squares
2666	France	Europe	farmland	presence	41 squares
2667	France	Europe	farmland	presence	46 squares
2668	France	Europe	farmland	absence	40 squares
2669	France	Europe	farmland	presence	45 squares
2670	France	Europe	farmland	presence	46 squares
2671	France	Europe	farmland	absence	23 squares
2672	France	Europe	farmland	presence	30 squares
2673	France	Europe	farmland	presence	54 squares
2674	France	Europe	farmland	absence	50 squares
2675	France	Europe	farmland	presence	38 squares
2676	France	Europe	farmland	presence	45 squares
2677	France	Europe	farmland	absence	41 squares
2678	France	Europe	farmland	presence	37 squares
2679	France	Europe	farmland	presence	28 squares
2680	France	Europe	farmland	presence	41 squares
2681	France	Europe	farmland	presence	53 squares
2682	France	Europe	farmland	absence	44 squares
2683	France	Europe	farmland	presence	31 squares
2684	France	Europe	farmland	presence	37 squares
2685	France	Europe	farmland	presence	27 squares
2686	France	Europe	farmland	presence	32 squares
2687	France	Europe	farmland	presence	42 squares
2688	France	Europe	farmland	presence	28 squares
2689	France	Europe	farmland	presence	52 squares
2690	France	Europe	farmland	presence	52 squares
2691	France	Europe	farmland	presence	53 squares
2692	France	Europe	farmland	presence	42 squares
2693	France	Europe	farmland	presence	53 squares
2694	France	Europe	farmland	presence	46 squares
2695	France	Europe	farmland	presence	42 squares
2696	France	Europe	farmland	presence	34 squares
2697	France	Europe	farmland	presence	52 squares
2698	France	Europe	farmland	presence	37 squares
2699	France	Europe	farmland	absence	39 squares

2700	France	Europe	farmland	presence	41	squares
2701	France	Europe	farmland	absence	41	squares
2702	France	Europe	farmland	presence	34	squares
2703	France	Europe	farmland	presence	33	squares
2704	France	Europe	farmland	presence	23	squares
2705	France	Europe	farmland	presence	40	squares
2706	France	Europe	farmland	presence	36	squares
2707	France	Europe	farmland	presence	32	squares
2708	France	Europe	farmland	presence	27	squares
2709	France	Europe	farmland	absence	44	squares
2710	France	Europe	farmland	absence	28	squares
2711	France	Europe	farmland	absence	31	squares
2712	France	Europe	farmland	absence	31	squares
2713	France	Europe	farmland	presence	34	squares
2714	France	Europe	farmland	presence	23	squares
2715	France	Europe	farmland	presence	26	squares
2716	France	Europe	farmland	presence	29	squares
2717	France	Europe	farmland	presence	51	squares
2718	France	Europe	farmland	presence	37	squares
2719	France	Europe	farmland	presence	38	squares
2720	France	Europe	farmland	absence	33	squares
2721	France	Europe	farmland	presence	47	squares
2722	France	Europe	farmland	presence	41	squares
2723	France	Europe	farmland	presence	45	squares
2724	France	Europe	farmland	presence	46	squares
2725	France	Europe	farmland	presence	41	squares
2726	France	Europe	farmland	presence	35	squares
2727	France	Europe	farmland	presence	48	squares
2728	France	Europe	farmland	presence	39	squares
2729	France	Europe	farmland	presence	32	squares
2730	France	Europe	farmland	absence	43	squares
2731	France	Europe	farmland	presence	33	squares
2732	France	Europe	farmland	absence	41	squares
2733	France	Europe	farmland	presence	45	squares
2734	France	Europe	farmland	presence	22	squares
2735	France	Europe	farmland	absence	45	squares
2736	France	Europe	farmland	absence	46	squares
2737	France	Europe	farmland	presence	41	squares
2738	France	Europe	farmland	presence	43	squares
2739	France	Europe	farmland	presence	39	squares
2740	France	Europe	farmland	absence	39	squares
2741	France	Europe	farmland	presence	37	squares
2742	France	Europe	farmland	presence	24	squares
2743	France	Europe	farmland	absence	31	squares
2744	France	Europe	farmland	presence	34	squares
2745	France	Europe	farmland	presence	38	squares
2746	France	Europe	farmland	absence	26	squares
2747	France	Europe	farmland	presence	37	squares
2748	France	Europe	farmland	presence	37	squares
2749	France	Europe	farmland	presence	36	squares

2750	France	Europe	farmland	presence	42	squares
2751	France	Europe	farmland	presence	35	squares
2752	France	Europe	farmland	absence	41	squares
2753	France	Europe	farmland	presence	43	squares
2754	France	Europe	farmland	presence	36	squares
2755	France	Europe	farmland	absence	47	squares
2756	France	Europe	farmland	presence	26	squares
2757	France	Europe	farmland	absence	40	squares
2758	France	Europe	farmland	presence	41	squares
2759	France	Europe	farmland	presence	43	squares
2760	France	Europe	farmland	presence	41	squares
2761	France	Europe	farmland	presence	32	squares
2762	France	Europe	farmland	presence	38	squares
2763	France	Europe	farmland	absence	27	squares
2764	France	Europe	farmland	presence	44	squares
2765	France	Europe	farmland	presence	24	squares
2766	France	Europe	farmland	presence	31	squares
2767	France	Europe	farmland	presence	29	squares
2768	France	Europe	farmland	presence	42	squares
2769	France	Europe	farmland	presence	31	squares
2770	France	Europe	farmland	presence	29	squares
2771	France	Europe	farmland	presence	34	squares
2772	France	Europe	farmland	absence	28	squares
2773	France	Europe	farmland	presence	29	squares
2774	France	Europe	farmland	presence	36	squares
2775	France	Europe	farmland	presence	16	squares
2776	France	Europe	farmland	absence	36	squares
2777	France	Europe	farmland	presence	56	squares
2778	France	Europe	farmland	presence	52	squares
2779	France	Europe	farmland	presence	31	squares
2780	France	Europe	farmland	presence	24	squares
2781	France	Europe	farmland	presence	43	squares
2782	France	Europe	farmland	presence	34	squares
2783	France	Europe	farmland	presence	42	squares
2784	France	Europe	farmland	presence	48	squares
2785	France	Europe	farmland	presence	26	squares
2786	France	Europe	farmland	presence	45	squares
2787	France	Europe	farmland	absence	33	squares
2788	France	Europe	farmland	absence	25	squares
2789	France	Europe	farmland	presence	24	squares
2790	France	Europe	farmland	presence	49	squares
2791	France	Europe	farmland	absence	30	squares
2792	France	Europe	farmland	presence	36	squares
2793	France	Europe	farmland	presence	42	squares
2794	France	Europe	farmland	presence	58	squares
2795	France	Europe	farmland	presence	54	squares
2796	France	Europe	farmland	presence	32	squares
2797	France	Europe	farmland	presence	34	squares
2798	France	Europe	farmland	presence	47	squares
2799	France	Europe	farmland	presence	41	squares

2800	France	Europe	farmland	absence	39 squares
2801	France	Europe	farmland	presence	50 squares
2802	France	Europe	farmland	presence	35 squares
2803	France	Europe	farmland	presence	32 squares
2804	France	Europe	farmland	absence	30 squares
2805	France	Europe	farmland	presence	37 squares
2806	France	Europe	farmland	presence	35 squares
2807	France	Europe	farmland	presence	27 squares
2808	France	Europe	farmland	absence	33 squares
2809	France	Europe	farmland	absence	15 squares
2810	France	Europe	farmland	presence	46 squares
2811	France	Europe	farmland	presence	46 squares
2812	France	Europe	farmland	absence	31 squares
2813	France	Europe	farmland	presence	34 squares
2814	France	Europe	farmland	absence	25 squares
2815	France	Europe	farmland	presence	26 squares
2816	France	Europe	farmland	absence	36 squares
2817	France	Europe	farmland	absence	31 squares
2818	France	Europe	farmland	presence	37 squares
2819	France	Europe	farmland	presence	41 squares
2820	France	Europe	farmland	presence	32 squares
2821	France	Europe	farmland	presence	41 squares
2822	France	Europe	farmland	presence	32 squares
2823	France	Europe	farmland	absence	32 squares
2824	France	Europe	farmland	presence	40 squares
2825	France	Europe	farmland	presence	26 squares
2826	France	Europe	farmland	presence	44 squares
2827	France	Europe	farmland	presence	35 squares
2828	France	Europe	farmland	presence	29 squares
2829	France	Europe	farmland	presence	31 squares
2830	France	Europe	farmland	absence	31 squares
2831	France	Europe	farmland	presence	31 squares
2832	France	Europe	farmland	absence	24 squares
2833	France	Europe	farmland	presence	26 squares
2834	France	Europe	farmland	presence	34 squares
2835	France	Europe	farmland	presence	30 squares
2836	France	Europe	farmland	presence	26 squares
2837	France	Europe	farmland	presence	33 squares
2838	France	Europe	farmland	presence	41 squares
2839	France	Europe	farmland	presence	46 squares
2840	France	Europe	farmland	presence	41 squares
2841	France	Europe	farmland	presence	42 squares
2842	France	Europe	farmland	presence	41 squares
2843	France	Europe	farmland	absence	48 squares
2844	France	Europe	farmland	presence	43 squares
2845	France	Europe	farmland	presence	46 squares
2846	France	Europe	farmland	presence	52 squares
2847	France	Europe	farmland	presence	44 squares
2848	France	Europe	farmland	presence	32 squares
2849	France	Europe	farmland	presence	49 squares

2850	France	Europe	farmland	absence	36	squares
2851	France	Europe	farmland	absence	36	squares
2852	France	Europe	farmland	presence	55	squares
2853	France	Europe	farmland	presence	30	squares
2854	France	Europe	farmland	absence	32	squares
2855	France	Europe	farmland	presence	42	squares
2856	France	Europe	farmland	presence	47	squares
2857	France	Europe	farmland	presence	38	squares
2858	France	Europe	farmland	absence	33	squares
2859	France	Europe	farmland	absence	27	squares
2860	France	Europe	farmland	presence	39	squares
2861	France	Europe	farmland	absence	25	squares
2862	France	Europe	farmland	absence	23	squares
2863	France	Europe	farmland	presence	42	squares
2864	France	Europe	farmland	presence	49	squares
2865	France	Europe	farmland	presence	41	squares
2866	France	Europe	farmland	presence	22	squares
2867	France	Europe	farmland	presence	45	squares
2868	France	Europe	farmland	absence	31	squares
2869	France	Europe	farmland	absence	34	squares
2870	France	Europe	farmland	absence	33	squares
2871	France	Europe	farmland	absence	35	squares
2872	France	Europe	farmland	absence	45	squares
2873	France	Europe	farmland	presence	46	squares
2874	France	Europe	farmland	presence	36	squares
2875	France	Europe	farmland	presence	44	squares
2876	France	Europe	farmland	presence	42	squares
2877	France	Europe	farmland	presence	41	squares
2878	France	Europe	farmland	presence	41	squares
2879	France	Europe	farmland	presence	46	squares
2880	France	Europe	farmland	presence	40	squares
2881	France	Europe	farmland	presence	40	squares
2882	France	Europe	farmland	presence	54	squares
2883	France	Europe	farmland	presence	46	squares
2884	France	Europe	farmland	presence	44	squares
2885	France	Europe	farmland	absence	31	squares
2886	France	Europe	farmland	presence	41	squares
2887	France	Europe	farmland	presence	24	squares
2888	France	Europe	farmland	presence	38	squares
2889	France	Europe	farmland	presence	40	squares
2890	France	Europe	farmland	presence	40	squares
2891	France	Europe	farmland	presence	45	squares
2892	France	Europe	farmland	presence	45	squares
2893	France	Europe	farmland	absence	34	squares
2894	France	Europe	farmland	presence	33	squares
2895	France	Europe	farmland	presence	43	squares
2896	France	Europe	farmland	presence	44	squares
2897	France	Europe	farmland	presence	34	squares
2898	France	Europe	farmland	absence	43	squares
2899	France	Europe	farmland	absence	42	squares

2900	France	Europe	farmland	presence	35	squares
2901	France	Europe	farmland	presence	40	squares
2902	France	Europe	farmland	presence	51	squares
2903	France	Europe	farmland	presence	44	squares
2904	France	Europe	farmland	presence	48	squares
2905	France	Europe	farmland	presence	50	squares
2906	France	Europe	farmland	presence	42	squares
2907	France	Europe	farmland	presence	39	squares
2908	France	Europe	farmland	presence	44	squares
2909	France	Europe	farmland	presence	40	squares
2910	France	Europe	farmland	presence	41	squares
2911	France	Europe	farmland	presence	40	squares
2912	France	Europe	farmland	presence	37	squares
2913	France	Europe	farmland	presence	36	squares
2914	France	Europe	farmland	presence	42	squares
2915	France	Europe	farmland	presence	39	squares
2916	France	Europe	farmland	presence	49	squares
2917	France	Europe	farmland	presence	46	squares
2918	France	Europe	farmland	presence	48	squares
2919	France	Europe	farmland	presence	50	squares
2920	France	Europe	farmland	presence	54	squares
2921	France	Europe	farmland	presence	40	squares
2922	France	Europe	farmland	absence	42	squares
2923	France	Europe	farmland	presence	41	squares
2924	France	Europe	farmland	presence	39	squares
2925	France	Europe	farmland	presence	43	squares
2926	France	Europe	farmland	presence	38	squares
2927	France	Europe	farmland	presence	42	squares
2928	France	Europe	farmland	presence	35	squares
2929	France	Europe	farmland	presence	32	squares
2930	France	Europe	farmland	presence	28	squares
2931	France	Europe	farmland	absence	37	squares
2932	France	Europe	farmland	presence	36	squares
2933	France	Europe	farmland	presence	37	squares
2934	France	Europe	farmland	presence	35	squares
2935	France	Europe	farmland	presence	38	squares
2936	France	Europe	farmland	presence	31	squares
2937	France	Europe	farmland	presence	36	squares
2938	France	Europe	farmland	presence	31	squares
2939	France	Europe	farmland	presence	42	squares
2940	France	Europe	farmland	presence	31	squares
2941	France	Europe	farmland	presence	40	squares
2942	France	Europe	farmland	presence	37	squares
2943	France	Europe	farmland	absence	27	squares
2944	France	Europe	farmland	presence	39	squares
2945	France	Europe	farmland	presence	39	squares
2946	France	Europe	farmland	presence	22	squares
2947	France	Europe	farmland	presence	28	squares
2948	France	Europe	farmland	absence	25	squares
2949	France	Europe	farmland	presence	26	squares

2950	France	Europe	farmland	presence	32	squares
2951	France	Europe	farmland	presence	34	squares
2952	France	Europe	farmland	absence	37	squares
2953	France	Europe	farmland	absence	24	squares
2954	France	Europe	farmland	presence	29	squares
2955	France	Europe	farmland	presence	38	squares
2956	France	Europe	farmland	presence	34	squares
2957	France	Europe	farmland	presence	25	squares
2958	France	Europe	farmland	presence	43	squares
2959	France	Europe	farmland	presence	41	squares
2960	France	Europe	farmland	absence	36	squares
2961	France	Europe	farmland	presence	30	squares
2962	France	Europe	farmland	presence	38	squares
2963	France	Europe	farmland	presence	42	squares
2964	France	Europe	farmland	presence	30	squares
2965	France	Europe	farmland	presence	30	squares
2966	France	Europe	farmland	presence	28	squares
2967	France	Europe	farmland	absence	34	squares
2968	France	Europe	farmland	presence	34	squares
2969	France	Europe	farmland	absence	24	squares
2970	France	Europe	farmland	absence	24	squares
2971	France	Europe	farmland	presence	36	squares
2972	France	Europe	farmland	absence	34	squares
2973	France	Europe	farmland	presence	47	squares
2974	France	Europe	farmland	presence	40	squares
2975	France	Europe	farmland	presence	29	squares
2976	France	Europe	farmland	presence	30	squares
2977	France	Europe	farmland	absence	11	squares
2978	France	Europe	farmland	absence	11	squares
2979	France	Europe	farmland	absence	5	squares
2980	France	Europe	farmland	absence	6	squares
2981	France	Europe	farmland	absence	7	squares
2982	France	Europe	farmland	absence	35	squares
2983	France	Europe	farmland	absence	39	squares
2984	France	Europe	farmland	absence	24	squares
2985	France	Europe	farmland	presence	32	squares
2986	France	Europe	farmland	absence	42	squares
2987	France	Europe	farmland	presence	37	squares
2988	France	Europe	farmland	presence	45	squares
2989	France	Europe	farmland	presence	38	squares
2990	France	Europe	farmland	absence	17	squares
2991	France	Europe	farmland	presence	29	squares
2992	France	Europe	farmland	presence	39	squares
2993	France	Europe	farmland	presence	31	squares
2994	France	Europe	farmland	presence	31	squares
2995	France	Europe	farmland	presence	40	squares
2996	France	Europe	farmland	presence	40	squares
2997	France	Europe	farmland	presence	40	squares
2998	France	Europe	farmland	absence	23	squares
2999	France	Europe	farmland	absence	25	squares

3000	France	Europe	farmland	absence	34 squares
3001	France	Europe	farmland	presence	29 squares
3002	France	Europe	farmland	absence	38 squares
3003	France	Europe	farmland	presence	27 squares
3004	France	Europe	farmland	presence	30 squares
3005	France	Europe	farmland	presence	33 squares
3006	France	Europe	farmland	presence	15 squares
3007	France	Europe	farmland	presence	37 squares
3008	France	Europe	farmland	presence	30 squares
3009	France	Europe	farmland	absence	40 squares
3010	France	Europe	farmland	presence	28 squares
3011	France	Europe	farmland	presence	33 squares
3012	France	Europe	farmland	absence	38 squares
3013	France	Europe	farmland	presence	37 squares
3014	France	Europe	farmland	absence	20 squares
3015	France	Europe	farmland	absence	34 squares
3016	France	Europe	farmland	presence	32 squares
3017	France	Europe	farmland	absence	34 squares
3018	France	Europe	farmland	presence	40 squares
3019	France	Europe	farmland	presence	22 squares
3020	France	Europe	farmland	presence	42 squares
3021	France	Europe	farmland	presence	44 squares
3022	France	Europe	farmland	presence	42 squares
3023	France	Europe	farmland	absence	34 squares
3024	France	Europe	farmland	presence	43 squares
3025	France	Europe	farmland	presence	46 squares
3026	France	Europe	farmland	presence	28 squares
3027	France	Europe	farmland	presence	21 squares
3028	France	Europe	farmland	presence	41 squares
3029	France	Europe	farmland	absence	43 squares
3030	France	Europe	farmland	presence	22 squares
3031	France	Europe	farmland	presence	31 squares
3032	France	Europe	farmland	presence	25 squares
3033	France	Europe	farmland	absence	22 squares
3034	France	Europe	farmland	presence	40 squares
3035	France	Europe	farmland	presence	31 squares
3036	France	Europe	farmland	absence	21 squares
3037	France	Europe	farmland	presence	22 squares
3038	France	Europe	farmland	presence	21 squares
3039	France	Europe	farmland	absence	20 squares
3040	France	Europe	farmland	absence	19 squares
3041	France	Europe	farmland	absence	18 squares
3042	France	Europe	farmland	absence	18 squares
3043	France	Europe	farmland	presence	31 squares
3044	France	Europe	farmland	absence	32 squares
3045	France	Europe	farmland	absence	31 squares
3046	France	Europe	farmland	absence	33 squares
3047	France	Europe	farmland	absence	23 squares
3048	France	Europe	farmland	presence	30 squares
3049	France	Europe	farmland	presence	33 squares

3050	France	Europe	farmland	absence	14 squares
3051	France	Europe	farmland	presence	30 squares
3052	France	Europe	farmland	presence	34 squares
3053	France	Europe	farmland	presence	39 squares
3054	France	Europe	farmland	presence	33 squares
3055	France	Europe	farmland	presence	38 squares
3056	France	Europe	farmland	presence	32 squares
3057	France	Europe	farmland	presence	51 squares
3058	France	Europe	farmland	presence	26 squares
3059	France	Europe	farmland	presence	41 squares
3060	France	Europe	farmland	presence	41 squares
3061	France	Europe	farmland	presence	36 squares
3062	France	Europe	farmland	presence	34 squares
3063	France	Europe	farmland	presence	42 squares
3064	France	Europe	farmland	presence	35 squares
3065	France	Europe	farmland	presence	34 squares
3066	France	Europe	farmland	presence	21 squares
3067	France	Europe	farmland	absence	24 squares
3068	France	Europe	farmland	presence	33 squares
3069	France	Europe	farmland	presence	38 squares
3070	France	Europe	farmland	presence	39 squares
3071	France	Europe	farmland	presence	28 squares
3072	France	Europe	farmland	presence	40 squares
3073	France	Europe	farmland	presence	39 squares
3074	France	Europe	farmland	presence	34 squares
3075	France	Europe	farmland	presence	19 squares
3076	France	Europe	farmland	presence	34 squares
3077	France	Europe	farmland	presence	35 squares
3078	France	Europe	farmland	presence	41 squares
3079	France	Europe	farmland	presence	37 squares
3080	France	Europe	farmland	absence	15 squares
3081	France	Europe	farmland	presence	35 squares
3082	France	Europe	farmland	presence	35 squares
3083	France	Europe	farmland	absence	31 squares
3084	France	Europe	farmland	presence	30 squares
3085	France	Europe	farmland	presence	27 squares
3086	France	Europe	farmland	presence	40 squares
3087	France	Europe	farmland	presence	41 squares
3088	France	Europe	farmland	presence	46 squares
3089	France	Europe	farmland	presence	32 squares
3090	France	Europe	farmland	presence	38 squares
3091	France	Europe	farmland	presence	41 squares
3092	France	Europe	farmland	presence	36 squares
3093	France	Europe	farmland	presence	27 squares
3094	France	Europe	farmland	presence	34 squares
3095	France	Europe	farmland	presence	42 squares
3096	France	Europe	farmland	presence	35 squares
3097	France	Europe	farmland	presence	32 squares
3098	France	Europe	farmland	presence	41 squares
3099	France	Europe	farmland	presence	32 squares

3100	France	Europe	farmland	presence	36	squares
3101	France	Europe	farmland	presence	33	squares
3102	France	Europe	farmland	presence	32	squares
3103	France	Europe	farmland	presence	34	squares
3104	France	Europe	farmland	presence	39	squares
3105	France	Europe	farmland	presence	36	squares
3106	France	Europe	farmland	presence	38	squares
3107	France	Europe	farmland	presence	39	squares
3108	France	Europe	farmland	presence	41	squares
3109	France	Europe	farmland	presence	42	squares
3110	France	Europe	farmland	presence	41	squares
3111	France	Europe	farmland	presence	41	squares
3112	France	Europe	farmland	presence	52	squares
3113	France	Europe	farmland	presence	56	squares
3114	France	Europe	farmland	presence	39	squares
3115	France	Europe	farmland	presence	48	squares
3116	France	Europe	farmland	presence	46	squares
3117	France	Europe	farmland	presence	41	squares
3118	France	Europe	farmland	presence	41	squares
3119	France	Europe	farmland	absence	35	squares
3120	France	Europe	farmland	presence	41	squares
3121	France	Europe	farmland	presence	50	squares
3122	France	Europe	farmland	absence	37	squares
3123	France	Europe	farmland	presence	39	squares
3124	France	Europe	farmland	presence	40	squares
3125	France	Europe	farmland	presence	40	squares
3126	France	Europe	farmland	presence	56	squares
3127	France	Europe	farmland	presence	38	squares
3128	France	Europe	farmland	presence	48	squares
3129	France	Europe	farmland	presence	42	squares
3130	France	Europe	farmland	presence	32	squares
3131	France	Europe	farmland	presence	40	squares
3132	France	Europe	farmland	absence	37	squares
3133	France	Europe	farmland	presence	39	squares
3134	France	Europe	farmland	absence	37	squares
3135	France	Europe	farmland	presence	33	squares
3136	France	Europe	farmland	presence	38	squares
3137	France	Europe	farmland	absence	23	squares
3138	France	Europe	farmland	presence	51	squares
3139	France	Europe	farmland	absence	30	squares
3140	France	Europe	farmland	presence	39	squares
3141	France	Europe	farmland	absence	25	squares
3142	France	Europe	farmland	presence	39	squares
3143	France	Europe	farmland	presence	41	squares
3144	France	Europe	farmland	presence	40	squares
3145	France	Europe	farmland	presence	40	squares
3146	France	Europe	farmland	presence	48	squares
3147	France	Europe	farmland	presence	48	squares
3148	France	Europe	farmland	presence	34	squares
3149	France	Europe	farmland	absence	44	squares

3150	France	Europe	farmland	presence	55	squares
3151	France	Europe	farmland	presence	56	squares
3152	France	Europe	farmland	presence	37	squares
3153	France	Europe	farmland	absence	43	squares
3154	France	Europe	farmland	presence	38	squares
3155	France	Europe	farmland	presence	53	squares
3156	France	Europe	farmland	presence	38	squares
3157	France	Europe	farmland	absence	40	squares
3158	France	Europe	farmland	presence	49	squares
3159	France	Europe	farmland	presence	48	squares
3160	France	Europe	farmland	presence	49	squares
3161	France	Europe	farmland	presence	49	squares
3162	France	Europe	farmland	absence	47	squares
3163	France	Europe	farmland	presence	47	squares
3164	France	Europe	farmland	absence	41	squares
3165	France	Europe	farmland	presence	48	squares
3166	France	Europe	farmland	absence	48	squares
3167	France	Europe	farmland	presence	46	squares
3168	France	Europe	farmland	presence	49	squares
3169	France	Europe	farmland	presence	33	squares
3170	France	Europe	farmland	presence	37	squares
3171	France	Europe	farmland	presence	49	squares
3172	France	Europe	farmland	absence	27	squares
3173	France	Europe	farmland	absence	35	squares
3174	France	Europe	farmland	presence	43	squares
3175	France	Europe	farmland	presence	31	squares
3176	France	Europe	farmland	presence	42	squares
3177	France	Europe	farmland	absence	39	squares
3178	France	Europe	farmland	absence	29	squares
3179	France	Europe	farmland	absence	25	squares
3180	France	Europe	farmland	presence	28	squares
3181	France	Europe	farmland	presence	42	squares
3182	France	Europe	farmland	absence	27	squares
3183	France	Europe	farmland	presence	38	squares
3184	France	Europe	farmland	presence	30	squares
3185	France	Europe	farmland	absence	24	squares
3186	France	Europe	farmland	absence	29	squares
3187	France	Europe	farmland	absence	25	squares
3188	France	Europe	farmland	absence	22	squares
3189	France	Europe	farmland	absence	33	squares
3190	France	Europe	farmland	absence	21	squares
3191	France	Europe	farmland	absence	31	squares
3192	France	Europe	farmland	absence	28	squares
3193	France	Europe	farmland	presence	27	squares
3194	France	Europe	farmland	absence	25	squares
3195	France	Europe	farmland	absence	24	squares
3196	France	Europe	farmland	absence	24	squares
3197	France	Europe	farmland	absence	8	squares
3198	France	Europe	farmland	absence	6	squares
3199	France	Europe	farmland	absence	10	squares

3200	France	Europe	farmland	absence	16	squares
3201	France	Europe	farmland	presence	34	squares
3202	France	Europe	farmland	presence	41	squares
3203	France	Europe	farmland	absence	37	squares
3204	France	Europe	farmland	presence	33	squares
3205	France	Europe	farmland	presence	43	squares
3206	France	Europe	farmland	presence	39	squares
3207	France	Europe	farmland	presence	38	squares
3208	France	Europe	farmland	presence	45	squares
3209	France	Europe	farmland	presence	46	squares
3210	France	Europe	farmland	presence	40	squares
3211	France	Europe	farmland	presence	46	squares
3212	France	Europe	farmland	presence	44	squares
3213	France	Europe	farmland	presence	48	squares
3214	France	Europe	farmland	presence	46	squares
3215	France	Europe	farmland	absence	26	squares
3216	France	Europe	farmland	presence	25	squares
3217	France	Europe	farmland	presence	36	squares
3218	France	Europe	farmland	absence	19	squares
3219	France	Europe	farmland	presence	23	squares
3220	France	Europe	farmland	presence	46	squares
3221	France	Europe	farmland	absence	38	squares
3222	France	Europe	farmland	presence	26	squares
3223	France	Europe	farmland	presence	49	squares
3224	France	Europe	farmland	absence	37	squares
3225	France	Europe	farmland	presence	42	squares
3226	France	Europe	farmland	presence	39	squares
3227	France	Europe	farmland	presence	42	squares
3228	France	Europe	farmland	presence	50	squares
3229	France	Europe	farmland	presence	37	squares
3230	Finland	Europe	forest	presence	9	point count
3231	Finland	Europe	forest	presence	9	point count
3232	Finland	Europe	forest	presence	10	point count
3233	Finland	Europe	forest	presence	9	point count
3234	Finland	Europe	forest	presence	9	point count
3235	Finland	Europe	forest	presence	8	point count
3236	Finland	Europe	forest	presence	8	point count
3237	Finland	Europe	forest	presence	9	point count
3238	Finland	Europe	forest	presence	8	point count
3239	Finland	Europe	forest	presence	9	point count
3240	Finland	Europe	forest	absence	4	point count
3241	Finland	Europe	forest	absence	4	point count
3242	Finland	Europe	forest	absence	4	point count
3243	Finland	Europe	forest	absence	3	point count
3244	Finland	Europe	forest	presence	5	point count
3245	Finland	Europe	forest	absence	6	point count
3246	Finland	Europe	forest	presence	7	point count
3247	Finland	Europe	forest	presence	6	point count
3248	Finland	Europe	forest	presence	4	point count
3249	Finland	Europe	forest	absence	4	point count

3250	Finland	Europe	forest	absence	4	point count
3251	Finland	Europe	forest	absence	4	point count
3252	Finland	Europe	forest	absence	5	point count
3253	Finland	Europe	forest	absence	6	point count
3254	Finland	Europe	forest	absence	8	point count
3255	Finland	Europe	forest	absence	9	point count
3256	Finland	Europe	forest	presence	7	point count
3257	Finland	Europe	forest	absence	9	point count
3258	Finland	Europe	forest	presence	9	point count
3259	Finland	Europe	forest	presence	10	point count
3260	Finland	Europe	forest	absence	6	point count
3261	Finland	Europe	forest	presence	7	point count
3262	Finland	Europe	forest	presence	12	point count
3263	Finland	Europe	forest	presence	7	point count
3264	Finland	Europe	forest	presence	9	point count
3265	Finland	Europe	forest	presence	8	point count
3266	Finland	Europe	forest	absence	6	point count
3267	Finland	Europe	forest	absence	6	point count
3268	Finland	Europe	forest	presence	8	point count
3269	Finland	Europe	forest	absence	10	point count
3270	Finland	Europe	forest	presence	8	point count
3271	Finland	Europe	forest	absence	9	point count
3272	Finland	Europe	forest	absence	9	point count
3273	Finland	Europe	forest	absence	5	point count
3274	Finland	Europe	forest	absence	8	point count
3275	Finland	Europe	forest	presence	8	point count
3276	Finland	Europe	forest	presence	11	point count
3277	Finland	Europe	forest	presence	10	point count
3278	Finland	Europe	forest	presence	9	point count
3279	Finland	Europe	forest	presence	8	point count
3280	Finland	Europe	forest	presence	12	point count
3281	Finland	Europe	forest	presence	10	point count
3282	Finland	Europe	forest	presence	10	point count
3283	Finland	Europe	forest	presence	11	point count
3284	Finland	Europe	forest	absence	7	point count
3285	Finland	Europe	forest	absence	4	point count
3286	Finland	Europe	forest	presence	5	point count
3287	Finland	Europe	forest	presence	6	point count
3288	Finland	Europe	forest	absence	3	point count
3289	Finland	Europe	forest	absence	3	point count
3290	Finland	Europe	forest	absence	5	point count
3291	Finland	Europe	forest	presence	5	point count
3292	Finland	Europe	forest	absence	6	point count
3293	Finland	Europe	forest	absence	6	point count
3294	Finland	Europe	forest	presence	6	point count
3295	Finland	Europe	forest	absence	5	point count
3296	Finland	Europe	forest	absence	5	point count
3297	Finland	Europe	forest	absence	6	point count
3298	Finland	Europe	forest	absence	4	point count
3299	Finland	Europe	forest	presence	4	point count

3300	Finland	Europe	forest	presence	6	point count
3301	Finland	Europe	forest	presence	8	point count
3302	Finland	Europe	forest	presence	4	point count
3303	Finland	Europe	forest	presence	8	point count
3304	Finland	Europe	forest	presence	6	point count
3305	Finland	Europe	forest	presence	6	point count
3306	Finland	Europe	forest	absence	5	point count
3307	Finland	Europe	forest	presence	5	point count
3308	Finland	Europe	forest	absence	4	point count
3309	Finland	Europe	forest	presence	7	point count
3310	Finland	Europe	forest	absence	7	point count
3311	Finland	Europe	forest	absence	6	point count
3312	Finland	Europe	forest	absence	6	point count
3313	Finland	Europe	forest	presence	11	point count
3314	Finland	Europe	forest	presence	8	point count
3315	Finland	Europe	forest	presence	5	point count
3316	Finland	Europe	forest	absence	5	point count
3317	Finland	Europe	forest	presence	4	point count
3318	Finland	Europe	forest	presence	4	point count
3319	Finland	Europe	forest	absence	4	point count
3320	Finland	Europe	forest	presence	8	point count
3321	Finland	Europe	forest	presence	6	point count
3322	Finland	Europe	forest	absence	5	point count
3323	Finland	Europe	forest	presence	7	point count
3324	Finland	Europe	forest	presence	5	point count
3325	Finland	Europe	forest	presence	7	point count
3326	Finland	Europe	forest	presence	9	point count
3327	Finland	Europe	forest	presence	8	point count
3328	Finland	Europe	forest	presence	6	point count
3329	Finland	Europe	forest	presence	8	point count
3330	Finland	Europe	forest	presence	8	point count
3331	Finland	Europe	forest	presence	8	point count
3332	Finland	Europe	forest	presence	9	point count
3333	Finland	Europe	forest	presence	9	point count
3334	Finland	Europe	forest	presence	8	point count
3335	Finland	Europe	forest	presence	5	point count
3336	Finland	Europe	forest	presence	9	point count
3337	Finland	Europe	forest	presence	8	point count
3338	Finland	Europe	forest	presence	7	point count
3339	Finland	Europe	forest	presence	8	point count
3340	Finland	Europe	forest	presence	5	point count
3341	Finland	Europe	forest	presence	7	point count
3342	Finland	Europe	forest	presence	8	point count
3343	Finland	Europe	forest	presence	8	point count
3344	Finland	Europe	forest	presence	7	point count
3345	Finland	Europe	forest	presence	7	point count
3346	Finland	Europe	forest	presence	6	point count
3347	Finland	Europe	forest	presence	7	point count
3348	Finland	Europe	forest	presence	6	point count
3349	Finland	Europe	forest	presence	5	point count

3350	Finland	Europe	forest	absence	11	point count
3351	Finland	Europe	forest	absence	7	point count
3352	Finland	Europe	forest	absence	5	point count
3353	Finland	Europe	forest	absence	6	point count
3354	Finland	Europe	forest	absence	7	point count
3355	Finland	Europe	forest	presence	7	point count
3356	Finland	Europe	forest	absence	5	point count
3357	Finland	Europe	forest	absence	4	point count
3358	Finland	Europe	forest	presence	8	point count
3359	Finland	Europe	forest	presence	5	point count
3360	Finland	Europe	forest	presence	5	point count
3361	Finland	Europe	forest	absence	5	point count
3362	Finland	Europe	forest	presence	4	point count
3363	Finland	Europe	forest	absence	7	point count
3364	Finland	Europe	forest	presence	10	point count
3365	Finland	Europe	forest	absence	5	point count
3366	Finland	Europe	forest	presence	8	point count
3367	Finland	Europe	forest	presence	8	point count
3368	Finland	Europe	forest	absence	8	point count
3369	Finland	Europe	forest	absence	8	point count
3370	Finland	Europe	forest	absence	10	point count
3371	Finland	Europe	forest	presence	8	point count
3372	Finland	Europe	forest	absence	5	point count
3373	Finland	Europe	forest	presence	7	point count
3374	Finland	Europe	forest	presence	7	point count
3375	Finland	Europe	forest	absence	6	point count
3376	Finland	Europe	forest	absence	8	point count
3377	Finland	Europe	forest	presence	10	point count
3378	Finland	Europe	forest	presence	5	point count
3379	Finland	Europe	forest	presence	8	point count
3380	Finland	Europe	forest	presence	5	point count
3381	Finland	Europe	forest	presence	4	point count
3382	Finland	Europe	forest	presence	7	point count
3383	Finland	Europe	forest	presence	8	point count
3384	Finland	Europe	forest	presence	7	point count
3385	Finland	Europe	forest	presence	6	point count
3386	Finland	Europe	forest	absence	4	point count
3387	Finland	Europe	forest	presence	5	point count
3388	China	Asia	open area with house (forest edge)	presence	11	point count
3389	China	Asia	open area with house (forest edge)	absence	8	point count
3390	China	Asia	open area with house (forest edge)	absence	5	point count
3391	China	Asia	open area with house (forest edge)	absence	8	point count
3392	China	Asia	tea garden	presence	13	point count
3393	China	Asia	tea garden	absence	6	point count
3394	China	Asia	tea garden	absence	10	point count
3395	China	Asia	tea garden	absence	4	point count
3396	China	Asia	tea garden	absence	5	point count
3397	China	Asia	tea garden	absence	9	point count
3398	China	Asia	tea garden	absence	8	point count
3399	China	Asia	regenerating forest	absence	5	point count

3400	China	Asia	regenerating forest	absence	8	point count
3401	China	Asia	regenerating forest	absence	5	point count
3402	China	Asia	regenerating forest	absence	6	point count
3403	China	Asia	regenerating forest	absence	4	point count
3404	China	Asia	regenerating forest	absence	5	point count
3405	China	Asia	regenerating forest	absence	2	point count
3406	China	Asia	regenerating forest	absence	5	point count
3407	China	Asia	regenerating forest	absence	3	point count
3408	China	Asia	regenerating forest	absence	5	point count
3409	China	Asia	regenerating forest	absence	3	point count
3410	China	Asia	regenerating forest	absence	4	point count
3411	China	Asia	regenerating forest	absence	10	point count
3412	China	Asia	open area with house (forest edge)	absence	4	point count
3413	China	Asia	open area with house (forest edge)	absence	9	point count
3414	China	Asia	open area with house (forest edge)	absence	3	point count
3415	China	Asia	open area with house (forest edge)	absence	5	point count
3416	China	Asia	open area with house (forest edge)	absence	3	point count
3417	China	Asia	open area with house (forest edge)	absence	3	point count
3418	China	Asia	open area with house (forest edge)	absence	4	point count
3419	China	Asia	tea garden	absence	7	point count
3420	China	Asia	tea garden	absence	9	point count
3421	China	Asia	tea garden	absence	3	point count
3422	China	Asia	regenerating forest	absence	7	point count
3423	China	Asia	regenerating forest	absence	6	point count
3424	China	Asia	regenerating forest	absence	5	point count
3425	China	Asia	regenerating forest	absence	4	point count
3426	China	Asia	regenerating forest	absence	4	point count
3427	China	Asia	regenerating forest	absence	5	point count
3428	China	Asia	regenerating forest	absence	4	point count
3429	China	Asia	regenerating forest	absence	4	point count
3430	China	Asia	regenerating forest	absence	4	point count
3431	China	Asia	mature forest	absence	6	point count
3432	China	Asia	mature forest	absence	3	point count
3433	China	Asia	mature forest	absence	5	point count
3434	China	Asia	mature forest	absence	4	point count
3435	China	Asia	mature forest	absence	4	point count
3436	China	Asia	mature forest	absence	3	point count
3437	China	Asia	mature forest	absence	6	point count
3438	China	Asia	mature forest	absence	3	point count
3439	China	Asia	mature forest	absence	5	point count
3440	China	Asia	mature forest	absence	3	point count
3441	China	Asia	mature forest	absence	3	point count
3442	China	Asia	mature forest	absence	4	point count
3443	China	Asia	mature forest	absence	2	point count
3444	China	Asia	mature forest	absence	3	point count
3445	China	Asia	mature forest	absence	2	point count
3446	China	Asia	mature forest	absence	5	point count
3447	China	Asia	mature forest	absence	3	point count
3448	China	Asia	mature forest	absence	5	point count
3449	China	Asia	shrub	absence	4	point count

3450	China	Asia	shrub	absence	3	point count
3451	China	Asia	shrub	absence	5	point count
3452	China	Asia	shrub	absence	4	point count
3453	China	Asia	shrub	absence	7	point count
3454	China	Asia	shrub	absence	9	point count
3455	China	Asia	shrub	absence	6	point count
3456	China	Asia	shrub	absence	4	point count
3457	China	Asia	shrub	absence	7	point count
3458	China	Asia	shrub	absence	4	point count
3459	China	Asia	open area with house (forest edge)	absence	17	point count
3460	China	Asia	open area with house (forest edge)	presence	13	point count
3461	China	Asia	open area with house (forest edge)	absence	10	point count
3462	China	Asia	open area with house (forest edge)	absence	4	point count
3463	China	Asia	open area with house (forest edge)	absence	7	point count
3464	China	Asia	open area with house (forest edge)	absence	6	point count
3465	China	Asia	open area with house (forest edge)	absence	5	point count
3466	China	Asia	open area with house (forest edge)	absence	4	point count
3467	China	Asia	open area with house (forest edge)	absence	5	point count
3468	China	Asia	open area with house (forest edge)	absence	3	point count
3469	China	Asia	open area with house (forest edge)	absence	15	point count
3470	China	Asia	open area with house (forest edge)	absence	9	point count
3471	China	Asia	open area with house (forest edge)	absence	10	point count
3472	China	Asia	mature forest	absence	15	point count
3473	China	Asia	mature forest	absence	5	point count
3474	China	Asia	mature forest	absence	4	point count
3475	China	Asia	mature forest	absence	4	point count
3476	China	Asia	mature forest	absence	2	point count
3477	China	Asia	mature forest	absence	5	point count
3478	China	Asia	mature forest	absence	4	point count
3479	China	Asia	mature forest	absence	5	point count
3480	China	Asia	mature forest	absence	5	point count
3481	China	Asia	mature forest	absence	4	point count
3482	China	Asia	mature forest	absence	4	point count
3483	China	Asia	mature forest	absence	4	point count
3484	China	Asia	mature forest	absence	6	point count
3485	China	Asia	mature forest	absence	5	point count
3486	China	Asia	mature forest	absence	3	point count
3487	China	Asia	mature forest	absence	5	point count
3488	China	Asia	mature forest	absence	7	point count
3489	China	Asia	mature forest	absence	4	point count
3490	China	Asia	mature forest	absence	9	point count
3491	China	Asia	mature forest	absence	6	point count
3492	China	Asia	mature forest	absence	2	point count
3493	China	Asia	mature forest	absence	5	point count
3494	China	Asia	mature forest	absence	5	point count
3495	China	Asia	mature forest	absence	8	point count
3496	China	Asia	mature forest	absence	2	point count
3497	China	Asia	mature forest	absence	2	point count
3498	China	Asia	mature forest	absence	6	point count
3499	China	Asia	mature forest	absence	1	point count

3500	China	Asia	mature forest	absence	1	point count
3501	China	Asia	mature forest	absence	2	point count
3502	China	Asia	mature forest	absence	2	point count
3503	China	Asia	mature forest	absence	3	point count
3504	China	Asia	mature forest	absence	1	point count
3505	China	Asia	mature forest	absence	3	point count
3506	China	Asia	mature forest	absence	7	point count
3507	China	Asia	mature forest	absence	2	point count
3508	China	Asia	mature forest	absence	2	point count
3509	China	Asia	regenerating forest	absence	3	point count
3510	China	Asia	regenerating forest	absence	1	point count
3511	China	Asia	regenerating forest	absence	0	point count
3512	China	Asia	regenerating forest	absence	0	point count
3513	China	Asia	regenerating forest	absence	3	point count
3514	China	Asia	regenerating forest	absence	2	point count
3515	China	Asia	regenerating forest	absence	3	point count
3516	China	Asia	regenerating forest	absence	1	point count
3517	China	Asia	regenerating forest	absence	3	point count
3518	China	Asia	regenerating forest	absence	0	point count
3519	China	Asia	regenerating forest	absence	0	point count
3520	China	Asia	regenerating forest	absence	1	point count
3521	China	Asia	regenerating forest	absence	1	point count
3522	China	Asia	regenerating forest	absence	1	point count
3523	China	Asia	regenerating forest	absence	1	point count
3524	China	Asia	regenerating forest	absence	3	point count
3525	China	Asia	regenerating forest	absence	3	point count
3526	China	Asia	regenerating forest	absence	1	point count
3527	China	Asia	regenerating forest	absence	2	point count
3528	China	Asia	regenerating forest	absence	2	point count
3529	China	Asia	regenerating forest	absence	2	point count
3530	China	Asia	regenerating forest	absence	1	point count
3531	China	Asia	regenerating forest	absence	3	point count
3532	China	Asia	regenerating forest	absence	2	point count
3533	China	Asia	regenerating forest	absence	3	point count
3534	China	Asia	regenerating forest	absence	5	point count
3535	China	Asia	regenerating forest	absence	2	point count
3536	China	Asia	regenerating forest	absence	1	point count
3537	China	Asia	regenerating forest	absence	4	point count
3538	China	Asia	regenerating forest	absence	2	point count
3539	China	Asia	regenerating forest	absence	2	point count
3540	China	Asia	regenerating forest	absence	1	point count
3541	China	Asia	regenerating forest	absence	2	point count
3542	China	Asia	regenerating forest	absence	3	point count
3543	China	Asia	regenerating forest	absence	2	point count
3544	China	Asia	regenerating forest	absence	3	point count
3545	China	Asia	regenerating forest	absence	2	point count
3546	China	Asia	regenerating forest	absence	3	point count
3547	China	Asia	regenerating forest	absence	3	point count
3548	China	Asia	regenerating forest	absence	2	point count
3549	China	Asia	open area with house (forest edge)	absence	3	point count

3550	China	Asia	open area with house (forest edge)	absence	1	point count
3551	China	Asia	regenerating forest	absence	3	point count
3552	China	Asia	regenerating forest	absence	3	point count
3553	China	Asia	regenerating forest	absence	4	point count
3554	China	Asia	regenerating forest	absence	9	point count
3555	China	Asia	regenerating forest	absence	4	point count
3556	China	Asia	regenerating forest	absence	3	point count
3557	China	Asia	regenerating forest	absence	4	point count
3558	China	Asia	regenerating forest	absence	3	point count
3559	China	Asia	regenerating forest	absence	3	point count
3560	China	Asia	regenerating forest	absence	4	point count
3561	China	Asia	regenerating forest	absence	2	point count
3562	China	Asia	regenerating forest	absence	3	point count
3563	China	Asia	regenerating forest	absence	2	point count
3564	China	Asia	regenerating forest	absence	1	point count
3565	China	Asia	regenerating forest	absence	7	point count
3566	China	Asia	regenerating forest	absence	3	point count
3567	China	Asia	regenerating forest	absence	3	point count
3568	China	Asia	regenerating forest	absence	2	point count
3569	China	Asia	regenerating forest	absence	3	point count
3570	China	Asia	regenerating forest	absence	4	point count
3571	China	Asia	regenerating forest	absence	3	point count
3572	China	Asia	regenerating forest	absence	5	point count
3573	China	Asia	regenerating forest	absence	2	point count
3574	China	Asia	regenerating forest	absence	3	point count
3575	China	Asia	regenerating forest	absence	2	point count
3576	China	Asia	regenerating forest	absence	2	point count
3577	China	Asia	regenerating forest	absence	6	point count
3578	China	Asia	regenerating forest	absence	8	point count
3579	China	Asia	shrub	absence	6	point count
3580	China	Asia	shrub	absence	7	point count
3581	China	Asia	shrub	presence	11	point count
3582	China	Asia	shrub	absence	6	point count
3583	China	Asia	shrub	absence	4	point count
3584	China	Asia	shrub	absence	6	point count
3585	China	Asia	shrub	absence	4	point count
3586	China	Asia	shrub	absence	3	point count
3587	China	Asia	shrub	absence	1	point count
3588	China	Asia	shrub	absence	2	point count
3589	China	Asia	shrub	absence	9	point count
3590	China	Asia	shrub	absence	8	point count
3591	China	Asia	shrub	absence	4	point count
3592	China	Asia	shrub	absence	3	point count
